# Chemical and enzymatic modifications of 5-methylcytosine at the intersection of DNA damage, repair, and epigenetic reprogramming

**DOI:** 10.1371/journal.pone.0273509

**Published:** 2022-08-29

**Authors:** Tuvshintugs Baljinnyam, Mark L. Sowers, Chia Wei Hsu, James W. Conrad, Jason L. Herring, Linda C. Hackfeld, Lawrence C. Sowers

**Affiliations:** 1 Department of Pharmacology and Toxicology, University of Texas Medical Branch, Galveston, Texas, United States of America; 2 MD-PhD Combined Degree Program, University of Texas Medical Branch, Galveston, Texas, United States of America; 3 Department of Internal Medicine, University of Texas Medical Branch, Galveston, Texas, United States of America; University of South Alabama Mitchell Cancer Institute, UNITED STATES

## Abstract

The DNA of all living organisms is persistently damaged by endogenous reactions including deamination and oxidation. Such damage, if not repaired correctly, can result in mutations that drive tumor development. In addition to chemical damage, recent studies have established that DNA bases can be enzymatically modified, generating many of the same modified bases. Irrespective of the mechanism of formation, modified bases can alter DNA-protein interactions and therefore modulate epigenetic control of gene transcription. The simultaneous presence of both chemically and enzymatically modified bases in DNA suggests a potential intersection, or collision, between DNA repair and epigenetic reprogramming. In this paper, we have prepared defined sequence oligonucleotides containing the complete set of oxidized and deaminated bases that could arise from 5-methylcytosine. We have probed these substrates with human glycosylases implicated in DNA repair and epigenetic reprogramming. New observations reported here include: SMUG1 excises 5-carboxyuracil (5caU) when paired with A or G. Both TDG and MBD4 cleave 5-formyluracil and 5caU when mispaired with G. Further, TDG not only removes 5-formylcytosine and 5-carboxycytosine when paired with G, but also when mispaired with A. Surprisingly, 5caU is one of the best substrates for human TDG, SMUG1 and MBD4, and a much better substrate than T. The data presented here introduces some unexpected findings that pose new questions on the interactions between endogenous DNA damage, repair, and epigenetic reprogramming pathways.

## Introduction

Double-stranded DNA within cells is the repository for genetic information. DNA can be replicated and transcribed, but otherwise was initially considered to be metabolically inactive. In the 1970s studies by Lindahl and coworkers revealed that DNA is chemically reactive and can undergo hydrolysis reactions, including the deamination of bases, and the loss of purines and pyrimidines from DNA by hydrolytic cleavage of the glycosidic bond [1–4]. Other studies revealed that DNA bases can also undergo oxidative damage [5–7]. Observations of the endogenous damage of DNA led to the discovery of DNA repair enzymes and pathways by which this damage could be repaired [8–11]. Today, it is well established that the DNA of living cells undergoes hundreds, or thousands, of endogenous DNA damage events every day [12–14] and that multiple, partially overlapping, DNA repair pathways exist to repair most of this damage.

In addition to the canonical bases A, T, G and C, a fifth base, 5-methylcytosine (5mC), was identified in 1948 [15]. Subsequent studies revealed an inverse association between 5mC in gene promoter regions and gene transcription [16]. It is now known that 5mC is an epigenetic marker that can significantly increase the binding affinity of DNA-binding proteins which contain a methyl-binding domain (MBD) [17–20]. The initial binding of these proteins to methylated sites leads to the recruitment of histone-modifying enzymes that create a compact chromatin structure that inhibits transcription [21, 22]. Usually, cytosines in both strands of a symmetrical CpG dinucleotide are methylated. Following DNA replication, a 5mC base in the parental strand can direct the maintenance methyltransferase, DNMT1, to methylate cytosine in the progeny strand re-establishing symmetrical methylation at that CpG dinucleotide [23, 24]. This mechanism for the heritability of methylation patterns suggested an important role for DNA methylation in cellular development and differentiation, and it was initially thought that methylation patterns, once established, would not be reversible.

Alterations in cytosine methylation patterns are tied to cancer etiology in two distinct ways. First, methylation patterns are perturbed in most cancer cells [25, 26]. Aberrant methylation can lead to the transcriptional silencing of tumor suppressor genes, while loss of methylation can lead to the inappropriate expression of transforming genes. Mechanisms by which methylation patterns can be altered are poorly understood. Second, C to T (C>T) transition mutations dominate the mutational landscape of human tumors and these transitions occur with hotspot frequency at methylated CpG dinucleotides [27–30]. The hydrolytic deamination of 5mC generates thymine resulting in the formation of a T:G mispair. T:G mispairs are poorly repaired in mammalian cells which may explain, in part, recurrent C>T mutations found in human tumors [31]. The thymine methyl group can also undergo oxidation, generating 5-hydroxymethyluracil (5hmU), 5-formyluracil (5foU) and 5-carboxyuracil (5caU) [32–35]. In addition to hydrolytic deamination of 5mC to T, the 5-methyl group of 5mC can undergo nonenzymatic oxidation to form 5-hydroxymethylcytosine (5hmC), 5-formylcytosine (5foC) and 5-carboxycytosine (5caC) [36–38].

**Fig 1:**
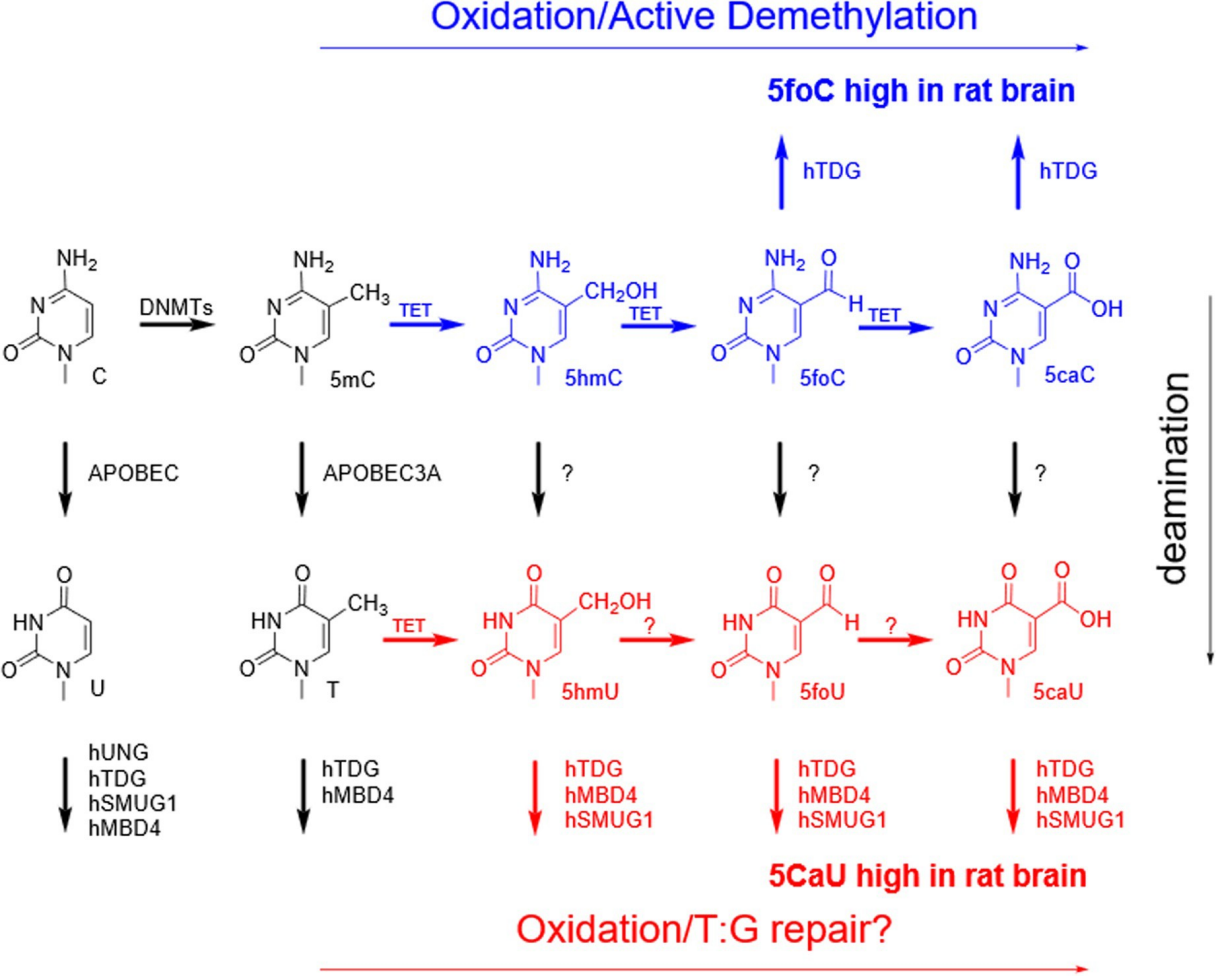
Possible active demethylation pathways resulting from the modification of 5mC. The oxidation of 5mC to 5foC and 5caC, and the removal of 5foC and 5caC by hTDG (blue) have been established. The glycosylase removal of 5hmU, 5foU and 5caU is established here (red). However, the enzymatic conversion of T to 5hmU, 5foU and 5caU (red) remains controversial.

In 2009, the Rao group identified an a-ketoglutarate dependent dioxygenase that could enzymatically convert 5mC to 5hmC in DNA [39]. The discovery of the TET enzymes led to a resurgence in studies on epigenetic reprogramming pathways [40–42]. The measurement of high levels of 5hmC in mammalian cells, particularly in neurons [43], established 5hmC as an important player in epigenetic reprogramming. Subsequent studies with the Tet enzymes revealed that 5mC could be converted not only to 5hmC, but to 5foC and 5caC as well (Fig 1) [44, 45]. The latter two products were then discovered to be substrates for Thymine DNA glycosylase [46]. This pathway has been proposed as the active DNA demethylation pathway.

Mechanistic studies of these DNA modification and repair pathways often rely upon synthetic oligonucleotides containing site-specific modified DNA bases. Our group developed methods to place both 5hmU [47] and 5hmC [48] into synthetic oligonucleotides. Subsequently, we showed that both modifications could interfere with DNA-protein interactions [49]. The replacement of 5mC by 5hmC not only inhibits the binding of MBD-containing proteins [20], but also blocks maintenance methylation by DNMT1 [24], providing mechanistic insight into how enzymatic modification of 5mC in DNA could alter epigenetics, even without further oxidation and or base excision repair.

Furthermore, additional DNA demethylation pathways utilizing the base excision repair (BER) pathway have also been suggested by invoking the deamination products of the cytosine analogs as shown in Fig 1 [50–53]. Following deamination, the corresponding uracil analogs could also be removed by members of the uracil DNA glycosylase superfamily, constituting a complete demethylation pathway. A family of DNA cytosine deaminases is known in human cells [50, 54, 55]. These deaminases account for hypermutation in variable regions of antibody genes in immune cells as well as the destruction of invading viral genomes. While some labs have presented evidence supporting a role for AID/APOBEC deamination of 5mC, and its oxidized analogs, other labs have presented contradictory evidence [50, 55].

An array of pyrimidine analogs arising from both oxidation and deamination are now known to occur in DNA by both enzymatic modification and nonenzymatic spontaneous damage. Currently, it remains unclear which of these modifications of 5mC represent DNA damage, bonafide epigenetic markers, or intermediates in either pathway. The simultaneous presence of modified bases from potentially multiple pathways reveals an intersection of DNA damage, repair, and epigenetic reprogramming.

To elucidate details of potential demethylation and DNA repair pathways, we have prepared a series of synthetic oligonucleotides containing the five cytosine and five uracil analogs shown in Fig 1. We have probed oligonucleotides containing these analogs when paired with A or G with recombinantly expressed human uracil DNA glycosylases including Uracil-DNA Glycosylase 2 (hUNG2), Single-strand selective Monofunctional Uracil DNA Glycosylase 1 (hSMUG1), Thymine DNA Glycosylase (hTDG) and Methyl-CpG-Binding Domain protein 4 (hMBD4). This is the first systematic study of all possible analogs with all relevant members of the BER pathway. The results obtained should shed new light on epigenetic remodeling and repair pathways as well as their possible intersection.

## Materials and methods

### Oligonucleotide synthesis and characterization

**Fig 2:**
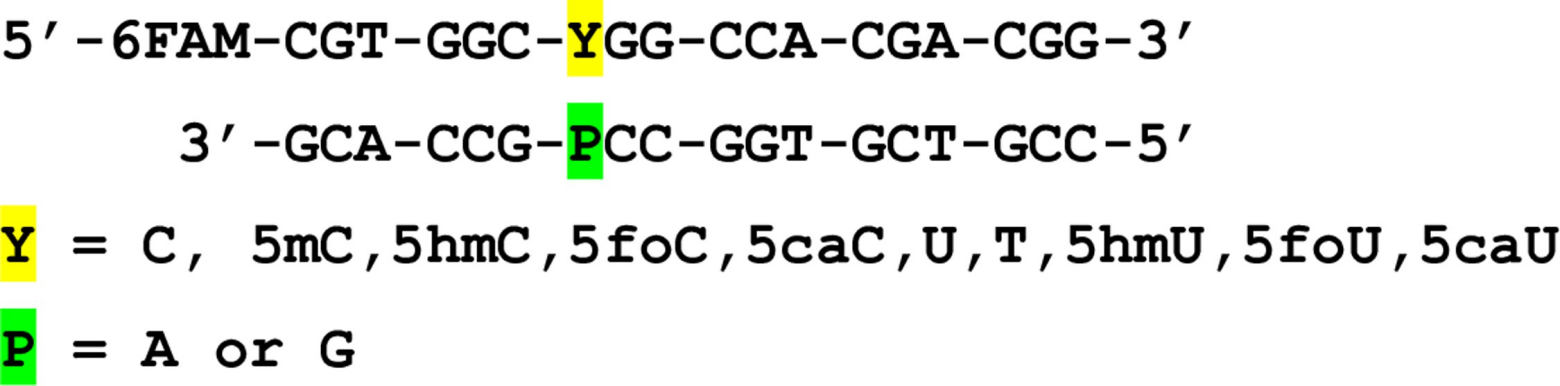
Sequence of the oligonucleotide duplex used in this study. See Fig 1 for structures of the pyrimidine analogs, ‘Y’.

Oligonucleotides were synthesized by standard phosphoramidite methods with an ABI Expedite 8909 nucleic acid synthesis system. Reagents for oligonucleotides synthesis were purchased from Glen Research, Sterling VA, including phosphoramidites of normal bases as well as for 5hmU, 5hmC, 5foC and 5caC. Oligonucleotides containing 5foU were made with unprotected 5-formyl-2’-deoxyuridine as described previously [56, 57]. Oligonucleotides containing 5caU were prepared according to the method previously reported [58, 59]. Sequences of oligonucleotides are shown in Fig 2.

Oligonucleotides were purified DMT-on with a PRP column and on a reverse phase column following detritylation. Oligonucleotides were characterized by gel electrophoresis and MALDI-TOF-MS. Oligonucleotide composition was verified by GC-MS analysis following formic acid hydrolysis and by HPLC following enzymatic hydrolysis as reported previously.

### Protein expression and characterization

Human hUNG2 (UNG-1547H) was obtained from Creative BioMart (Shirley, NY).

Plasmid DNA (pET28c(+)) encoding full length hTDG (NM_003211.6) [60] with an N-terminal his-tag was obtained from Addgene (Watertown, MA, USA). Coding sequences for hSMUG1 (NM_001243788.2) [61] and hMBD4 (NM_003925.3) [62] also containing an N-terminal his-tag, were inserted into pET28a(+) vectors by Genscript (Piscataway, NJ).

Plasmid DNAs were transformed into *E*. *coli* strain BL21 (DE3) and transformants were selected on LB + 1.4% agar plates containing kanamycin (50 mg/mL). After confirmation of target protein expression, cells were grown in 100 mL of LB broth supplemented with kanamycin (50 mg/mL) at 37°C until the OD_600_ increased to 0.6. Cells were then induced with IPTG (0.5 mM) and grown at 15°C for 22 h (hTDG) or 30°C for 6 h and then 15°C overnight (hSMUG1), or 30°C for 6 h (hMBD4). Cells were harvested by centrifugation at 4,100 rpm for 5 min and stored at -20°C until proteins were isolated.

The cell pellets were thawed and suspended in 4 mL of lysis buffer and sonicated for 8 cycles for 30 s with 30 s breaks on ice. Lysis buffer consisted of 50 mM potassium phosphate pH 8, 300 mM NaCl, 10 mM b-mercaptoethanol (bME), 20 mM imidazole, 1% Triton-X100, and 1 mM PMSF. After centrifugation at 12,000 rpm for 10 min, supernatants were loaded on previously equilibrated HisPur Ni-NTA resin and incubated for 1.5 hours at 4°C on a see-saw shaker. The suspension of HisPur Ni-NTA Resin beads and cell lysate was centrifuged at 1,000 g, 4°C for 5 min. Beads were washed sequentially with 3 mL of wash buffers A (x2), wash buffer B (x2), and wash buffer C (x6). Wash buffers contained 50 mM potassium phosphate pH 8, 300 mM NaCl, 10 mM bME, and progressively higher concentrations of imidazole: A, 5 mM; B, 10 mM; and C, 20 mM. His-tagged proteins were eluted from beads in 1 mL of elution buffer containing 300 mM NaCl, 10 mM bME and 100 mM imidazole. Protein concentrations were quantified with a Bradford protein assay using bovine serum albumin as a standard. Proteins were electrophoresed on a 12% tris-glycine SDS gel which was stained with Coomassie brilliant blue. Stained gels were scanned with ImageJ software and protein purity determined by densitometry.

### Glycosylase assays measured by gel electrophoresis

In the studies presented here, glycosylase excision of a target base generates an abasic site. Subsequent cleavage of the phosphodiester backbone at the abasic site generates a shorter, labelled oligonucleotide that can be resolved by polyacrylamide gel electrophoresis. Gels bands can be visualized and quantified to determine quantitatively the magnitude of glycosylase excision. Among the modified bases examined here, additional challenges exist with 5foU and 5foC. Our previous studies [57] have revealed that oligonucleotides containing 5foU can spontaneously cleave, especially when heated. Therefore, in the studies reported here, cleavage bands observed in the absence of a glycosylase were used as a control and subtracted from the glycosylase generated band.

To examine the substrate specificity of each glycosylase, 2.5 pmol of a FAM-labelled duplex containing a target pyrimidine pair opposite A or G was incubated with hUNG2 (0.07 mg, MW 40.0 kDa, 1.75 pmol), hTDG (1.5 mg, MW 48.4 kDa, 31 pmol), hSMUG1 (0.5 mg, 30.7 kDa, 16.3 pmol) in buffer containing 10 mM K_2_HPO_4_, 30mM NaCl, 40mM KCl, pH 7.9 or hMBD4 (0.5 mg, 66.9 kDa, 7.5 pmol) in buffer containing 20 mM tris-HCI, 1 mM DTT and 1mM EDTA at pH 8.0 and incubated for 1 h at 37°C. Recombinant hyTDG(Y163K) (0.5 mg, MW 29.7 kDa, 16.8 pmol) with no glycosylase activity, but efficient AP endonuclease activity [63, 64], was added and incubated at 65°C for 1 h to cleave the phosphodiester backbone at the abasic site. An equal volume of formamide was added and samples were loaded onto a 20% polyacrylamide gel containing 6M urea and 1xTBE buffer and electrophoresed at 180 V for 40 min using a Bio-Rad Mini-PROTEAN Tetra Cell (Hercules, CA). Gels were visualized with a Storm 860 gel imager.

Following target bases excision, oligonucleotide cleavage can be accomplished with an AP lyase or NaOH. In this study, when analogs were paired with G, the phosphodiester backbone were cleaved with a Y163K mutant of the hyTDG we recently described [63], which has lost glycosylase activity but gained AP lyase activity [64]. The advantage of the AP lyase is that neutral conditions employed do not result in chemical damage to modified bases. The disadvantage is that the AP lyase (Y163K) retains its selectivity for cleavage opposite G, underestimating glycosylase cleavage when the target pyrimidine is paired with A. Alternatively, oligonucleotides containing abasic sites can be cleaved in alkali, 0.1 M NaOH and heated to 96°C for 10 min. However, 0.1 M NaOH causes chemical cleavage of oligonucleotides containing 5foC. Therefore, we measured the cleavage of oligonucleotides containing target pyrimidines paired with A in the presence and absence of glycosylase and treated with NaOH. Cleavage in the absence of a glycosylase was then subtracted from any observed glycosylase cleavage.

### Glycosylase activity measured by GC-MS

Isotope standards for 5hmU, 5foU, 5caU, 5hmC, 5foC and 5caC were prepared as previously described [65]. Free bases released by glycosylases were measured using GC-MS. Briefly, 110 pmol of each oligonucleotide containing a target pyrimidine was combined with 2 equivalents of a complementary oligonucleotide. To that, a mixture of isotope-enriched pyrimidine standards containing 55 pmol of each standard described above, was mixed in a 10 mM potassium phosphate buffer, pH 7.7, 40 mM NaCl and 50 mM KCl. 100 pmol of each glycosylase was then added and samples were incubated at 37°C for 2 h in a total volume of 100 μL.

300 mL of de-ionized water was then added to each sample and vortexed. Released free bases were separated from oligonucleotides and proteins by spin filtration using a 3 kDa Amicon filter at 14,000 g for 40 min. The flow through was dried in a GC vial under reduced pressure. Pyrimidines were derivatized in 20 mL acetonitrile and 20 mL of N-(t-butyldimethylsilyl)-N-methyltrifluoroacetamide (MTBSTFA) with 1% tert-butyldimethylchlorosilane (TBDMCS) at 140°C for 40 min. Retention times and expected ions of free bases released from oligonucleotides as well as isotope-labeled standards are listed in S1 Table in S1 File.

0.5 mL of each sample was injected onto an Agilent 7890A GC equipped with an Agilent J&W DB-5MS + DG column (30 m × 0.25 mm id, film thickness 0.25 μm) using helium carrier gas at 1 mL/min constant flow. The GC oven temperature was held at 100°C for 2 min, ramped to 260°C at 30°C /min then held for 10 min. The GC was directly coupled to an Agilent 5975C Mass Selective Detector. Data was collected in the selected ion mode using ions appropriate for each analyte. Pyrimidines were quantified by comparing the peak area of the released pyrimidine to the peak area of the corresponding isotope standard.

## Results

### Preparation and validation of oligonucleotides and DNA uracil glycosylases

Oligonucleotides containing modified bases require special protection and deprotection strategies. Oligonucleotides containing the bases of interest (Fig 2) were synthesized using previously published methods, including methods for the synthesis of oligonucleotides containing 5hmU [47], 5foU [56, 57], 5caU [58, 59], 5hmC [48], 5foC [66–68] and 5caC [66, 67].

Oligonucleotides containing modified bases must be carefully characterized because deprotection may not occur as anticipated, unintended side reactions can occur, and the modified bases themselves can undergo further reactions. In this study, oligonucleotides were characterized by gel electrophoresis and MALDI-TOF mass spectrometry. The composition of each oligonucleotide was confirmed by HPLC analysis following enzymatic hydrolysis and by GC-MS analysis following formic acid hydrolysis as described previously.

Four members of the uracil DNA glycosylase superfamily are thought to be involved in the repair of deamination and methyl group oxidation and shown in Fig 1. Of these, hUNG2 was obtained commercially and three were prepared in our laboratory. Each enzyme was examined by gel electrophoresis and their sequence confirmed by LC-MS/MS following trypsin digestion.

### Glycosylase assay substrate selectivity

Oligonucleotide duplexes were prepared by mixing fluorescently tagged oligonucleotides containing target pyrimidines with complementary strands placing either A or G opposite the target pyrimidine (Fig 2). Oligonucleotide duplexes were then incubated with a molar excess of each glycosylase, followed by incubation with an AP-endonuclease to cleave the phosphodiester bond at abasic sites generated by glycosylase excision. Oligonucleotides were then separated by gel electrophoresis and visualized. Bands corresponding to intact and cleaved oligonucleotides were quantified. This was repeated a total of three times for each substrate.

**Fig 3:**
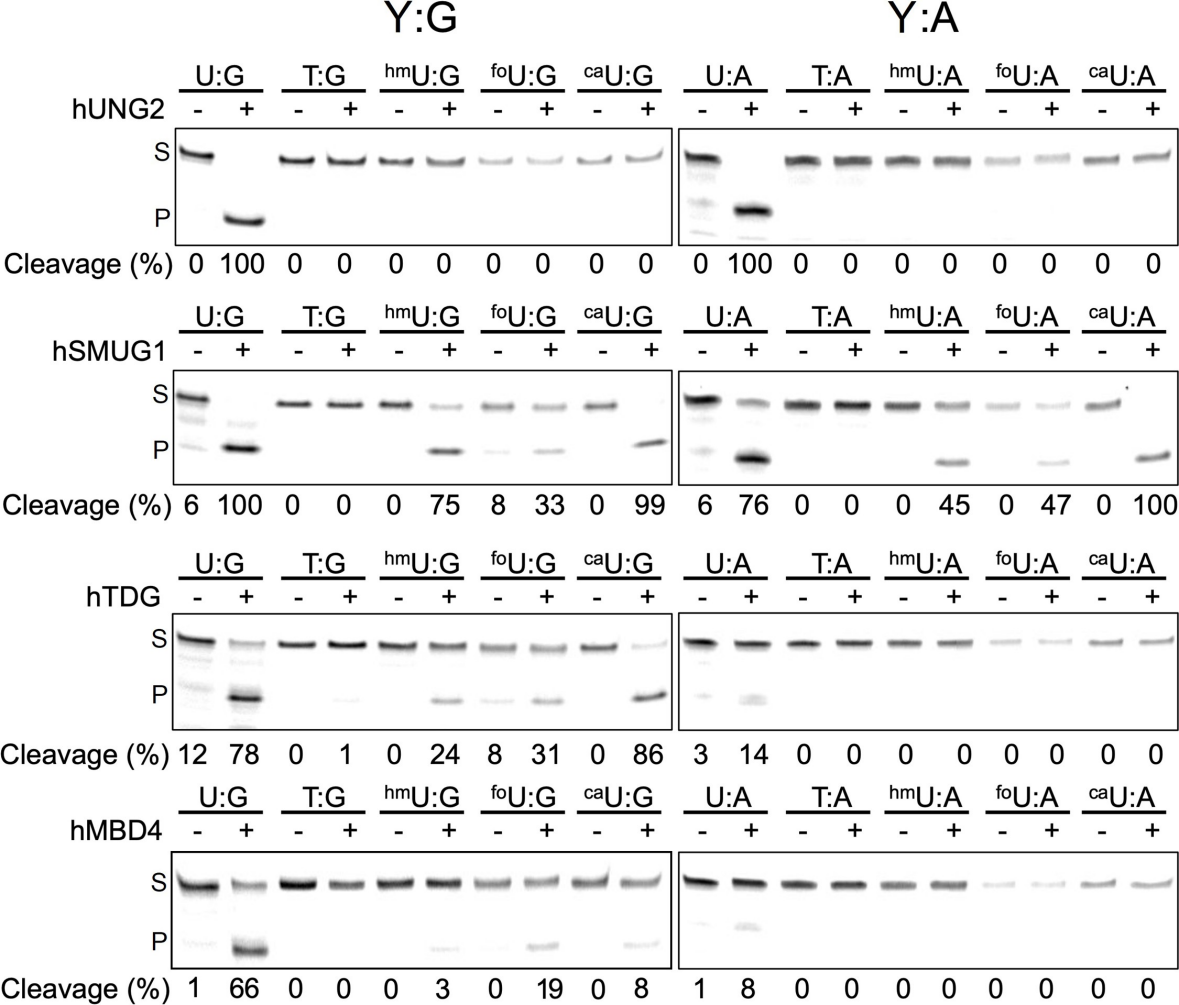
The excision specificity of human uracil family glycosylases for a series of 5-substituted uracil analogs (Y) paired with G (left) or A (right). FAM-labelled oligonucleotide duplexes (2.5 pmol) in buffer appropriate for each enzyme were incubated with a glycosylase at 37°C for 1 h. Duplexes paired with G were then incubated with hyTDG-lyase (Y163K) (65°C, 1 h) and those paired with A incubated with NaOH solution (96°C, 10 min) to cleave the abasic site. Oligonucleotides were resolved on a 20% polyacrylamide/urea gel, visualized on a STORM imager, and quantified with “Image quant” software. The amount of cleavage is shown below the lanes in each gel (S = substrate, P = product). Some artifact cleavage of 5foU-containing oligonucleotides is observed due to its thermal lability. Replicate gel images are shown in S1 and S2 Figs in S1 File.

**Fig 4:**
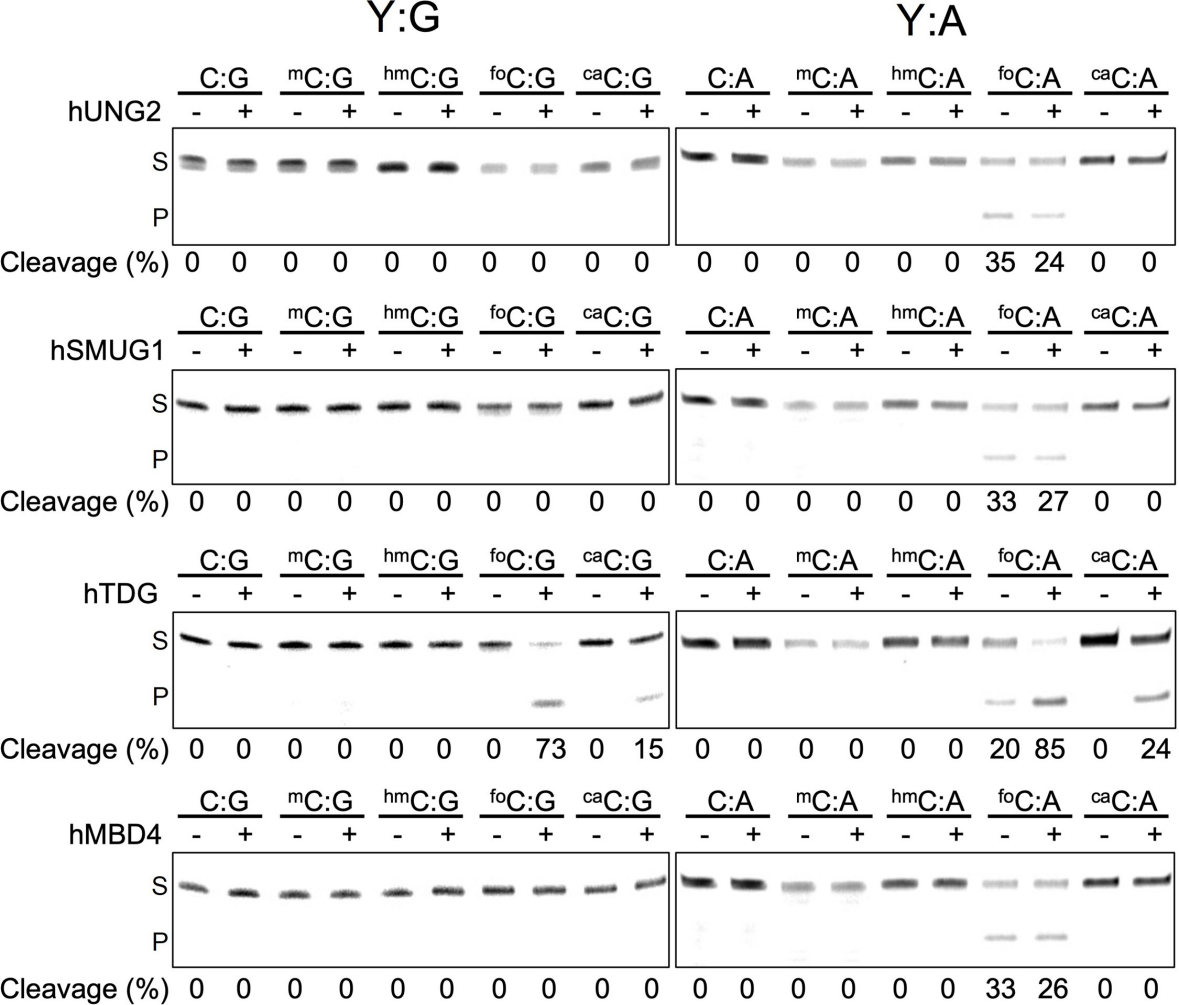
The excision specificity of human uracil family glycosylases for a series of 5-substituted cytosine analogs (Y) paired with G (left) or A (right). FAM-labelled oligonucleotide duplexes (2.5 pmol) in buffer appropriate for each enzyme were incubated with a glycosylase at 37°C for 1 h. Duplexes paired with G were then incubated with hyTDG-lyase (Y163K) (65°C, 1 h) and those paired with A incubated with NaOH solution (96°C, 10 min) to cleave the abasic site. Oligonucleotides were resolved on a 20% polyacrylamide/urea gel, visualized on a STORM imager, and quantified with “Image quant” software. The amount of cleavage is shown below the lanes in each gel (S = substrate, P = product). Some artifact cleavage of 5foC-containing oligonucleotides is observed due to its alkaline lability. Replicate gel images are shown in S3 and S4 Fig in S1 File.

Results of glycosylase excision for the oxidized uracil analogs paired with G or A are shown in Fig 3. For the oxidized cytosine analogs, the results are shown in Fig 4. As expected, hUNG2 had the narrowest substrate range. Among the substrates examined, hUNG2 cleaved only U and when present in a U:G mispair or U:A base pair. On the other hand, hSMUG1 had an increased substrate range that included U, 5hmU, 5foU and 5caU, but not T. As with hUNG2, target pyrimidines were excised when paired with G or with A. The overall substrate preference under these conditions for hSMUG1 was U ~ 5caU> 5hmU > 5foU when mispaired with G. When paired with A the order was 5caU > U > 5hmU ~ 5foU.

hTDG also had the broadest substrate range and cleaved all the oxidized uracil analogs, as well as T. However, target pyrimidines were excised much more efficiently when paired opposite G. The rank order for cleavage opposite G was 5caU > U > 5hmU ~ 5foU >> T. There was modest cleavage of U:A but other uracil analogs when paired with A were not detectable. Among the glycosylases examined here, hTDG was the only to cleave any of the cytosine analogs. The cytosine analogs 5foC and 5caC were excised when paired opposite either G or A.

The last glycosylase examined, hMBD4, had a narrower substrate range than hSMUG1 or hTDG. hMBD4 excised U, > 5foU > 5caU > hmU ~ T. Similar to hTDG, hMBD4 only removed U when paired with A, but not the other uracil analogs.

In this study as well as some previous studies, substantial cleavage of oligonucleotides containing 5foU could occur in the absence of a glycosylase, due to thermal hydrolysis of the glycosidic bond (65). Data presented in Table 1 was adjusted to account for observed spontaneous cleavage of 5foU in the negative control lane where no enzyme was added. Alkaline hydrolysis of 5foC paired with A similarly caused artifactual cleavage (Fig 4). Data presented in Table 1 was also adjusted to account for alkaline hydrolysis of 5foC-containing oligonucleotides in the absence of a glycosylase.

**Table 1:** Extent of cleavage for indicated bases in duplex oligonucleotides by members of the human uracil glycosylase family. Excision studies for analogs paired with G used a hyTDG-lyase (Y163K) to cleave the phosphodiester backbone whereas those paired with A were cleaved with NaOH. Values shown in the table represent the averages and standard deviations from three independent experiments. Cleavage of 5foU-containing oligonucleotides were adjusted for thermal hydrolysis by subtraction of cleavage observed in no enzyme controls. Cleavage of 5foC:A base pairs was adjusted for alkaline hydrolysis by subtraction of cleavage in no enzyme controls. Glycosylase excision of 5caC paired with A by hTDG was also measured using hyTDG-lyase (Y163K) and a value of 50.3±3.9% cleavage was observed. Phosphodiester backbone cleavage using the hyTDG-lyase (Y163K) underestimates glycosylase cleavage when the target pyrimidine is paired with A rather than G.

Substrate		hUNG2 (n = 3)	hSMUG1 (n = 3)	hTDG (n = 3)	hMBD4 (n = 3)
1	U:G	100±0	94±1.5	61±6.2	60±6.4
2	T:G	0	0	1±1	2±2.0
3	5hmU:G	0	76±3.2	19±8.9	2±0.6
4	5foU:G	0	28±5.5	29±11	23±13
5	5caU:G	0	98±3.2	81±6.4	9±4.0
6	U:A	100±0	67±4.9	12±4.9	9±4.0
7	T:A	0	0	0	0
8	5hmU:A	0	44±1.0	0	0
9	5foU:A	0	45±2.0	0	0
10	5caU:A	0	100±0	0	0
11	C:G	0	0	0	0
12	mC:G	0	0	0	0
13	5hmC:G	0	0	0	0
14	5foC:G	0	0	79±6.5	0
15	5caC:G	0	0	28±11	0
16	C:A	0	0	0	0
17	mC:A	0	0	0	0
18	5hmC:A	0	0	0	0
19	5foC:A	0	0	65±3.5	0
20	5caC:A	0	0	23±1.7	0

### Stable-isotope dilution gas-chromatography glycosylase validation

**Fig 5:**
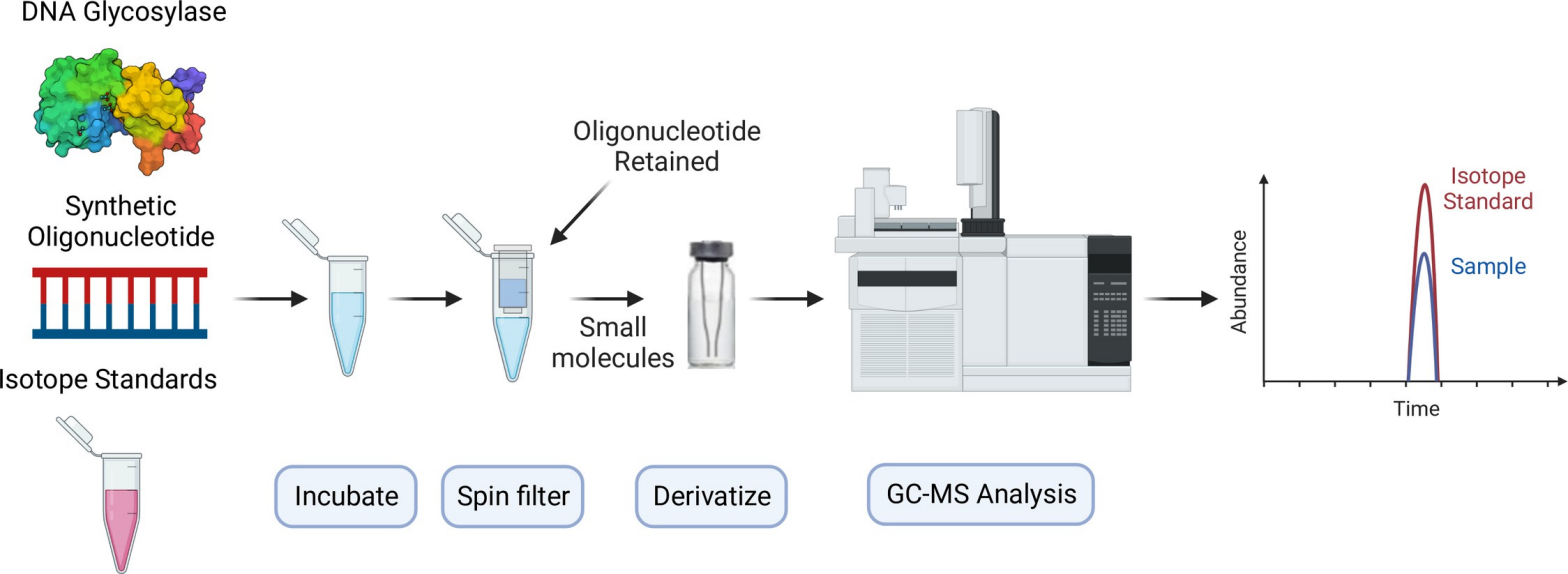
GC-MS scheme. This workflow illustrates the GC-MS based glycosylase assay to determine excision and base release from a synthetic oligonucleotide using stable-isotope standards.

Glycosylase reactions were repeated using a GC-MS based method (Fig 5). This was done to verify that cleavage of target pyrimidines indicated by the fluorescent gel-based assay was due to excision of the modified free base by breaking the N-glycosidic bond, consistent with monofunctional glycosylase activity. Oligonucleotide duplexes containing target pyrimidines were incubated with a glycosylase, and released free bases were separated with a spin filter and analyzed by GC-MS using stable-isotope-enriched standards.

**Fig 6:**
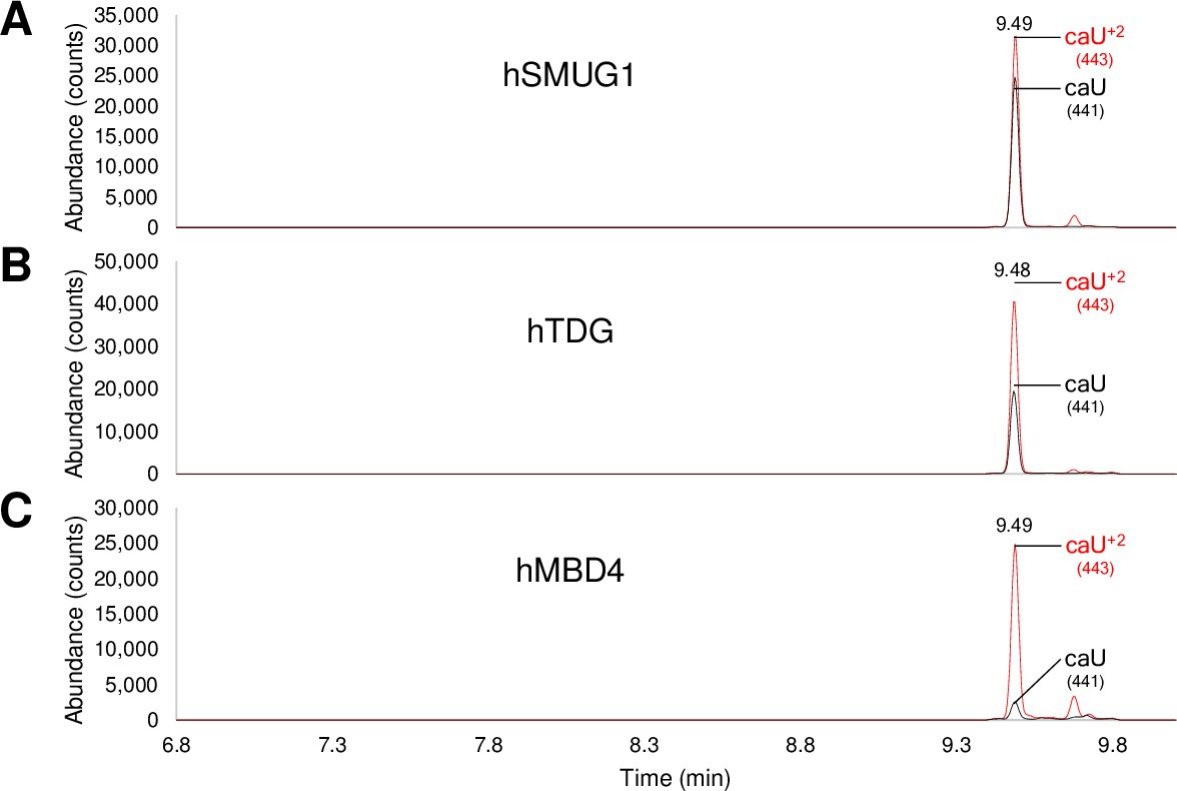
Confirmation of 5caU removal measured by GC-MS. A) 5caU:G treated with hSMUG1, B) 5caU:G treated with hTDG, and C) 5caU:G treated with hMBD4. A mixture of duplex oligo, containing a 5caU:G mispair (110 pmol) with a 2-fold excess of the complementary strand, and a 5caU+2 isotope labeled standard (55 pmol) was prepared in buffer: 10 mM potassium phosphate pH 7.7, 30 mM sodium chloride and 40 mM potassium chloride. The oligonucleotides and free base standard were incubated with approximately 100 pmol of either hSMUG1, hTDG or hMBD4 at 37°C for 2 h in a 110 μL reaction volume. Free bases were separated from enzymes and oligonucleotides by spin filtration, 90% of the sample was dried and analyzed by GC-MS. Derivatized free bases were separated by GC and identified by MS with selected ion monitoring. The ions for 5caU released by glycosylases (441 m/*z*, black) and 5caU+2 isotope enriched standard (443 m/*z*, red) were monitored, and the relative sizes of the integrated peak areas were used to calculate the amount of 5caU released. There was approximately 39, 24 and, 5 pmoles 5caU released by hSMUG1, hTDG and hMBD4, respectively. The overall removal of 5caU when paired opposite G by human glycosylases was hSMUG1 > hTDG > hMBD4, consistent with the trend seen by gel-based analysis of excision.

A total of 24 confirmatory GC-MS analyses were conducted, and all confirmed the findings from the gel-based assay (S5 to S28 Figs in S1 File). As an example, the release of 5caU when paired with G was verified for hTDG, hSMUG1 and hMBD4 (Fig 6). The expected retention time was confirmed by coelution with the authentic isotope standard, and the GC peak was collected when monitoring for the appropriate ions. In this case 441 amu was monitored for 5CaU released from the oligonucleotide and 443 amu for the isotope standard, 5CaU^+2^.

## Discussion

### Role of 5-methylcytosine in cancer and epigenetic regulation

The modified base, 5mC, is the primary epigenetic mark in the DNA of mammals. It has long been implicated as a contributor to cancer development. Transition mutations at methylated CpG sites are one of the most abundant single base changes observed in human tumors [27–30]. Additionally, cytosine methylation patterns are perturbed in most, if not all cancer cells. This results in inappropriate expression of transforming genes or silencing of tumor suppressor genes [25, 26].

Understanding mechanisms by which methylation patterns are established, and reprogrammed, may reveal potential defects in these pathways that could result in human disease. Our prior studies suggested that 5hmC might be an important intermediate in epigenetic reprogramming [20, 24, 51]; however, this pathway was not firmly established until the discovery of the TET family of oxidases. Subsequent studies have revealed additional 5mC analogs that may serve as epigenetic marks. The discovery of the enzymatic removal of 5foC and 5caC by TDG [46, 52, 69–73] provided a complete enzymatic pathway for active DNA demethylation. The participation of the BER pathway in epigenetic reprogramming, by excising analogs that could also be endogenous DNA damage products, introduces many intriguing questions.

### Systematic study of potential 5-methylcytosine analogs and DNA glycosylase activity

**Fig 7:**
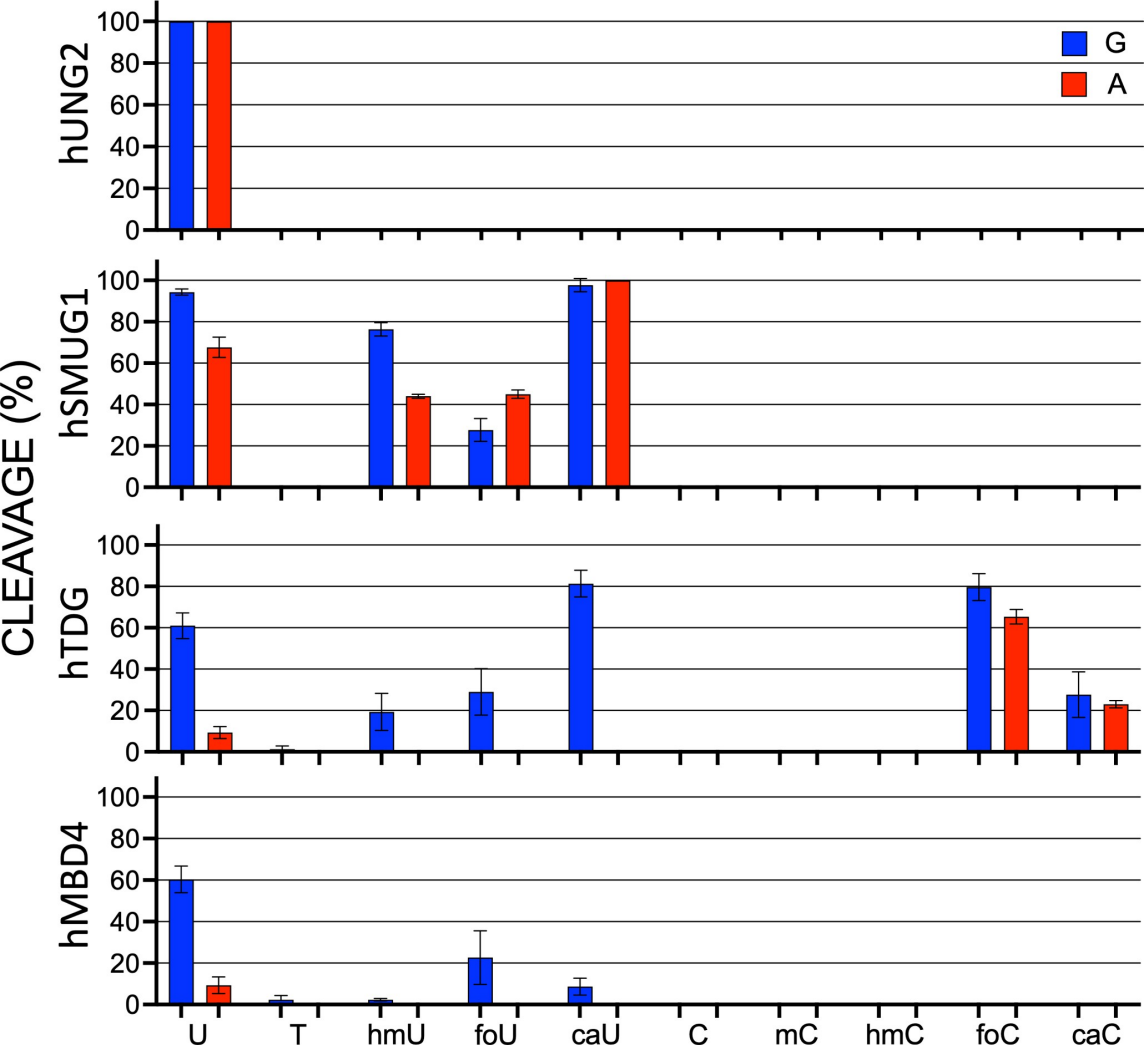
Summary for human glycosylase specificity for substrate. U, T, 5hmU, 5foU, 5caU, C, 5mC, 5hmC, 5foC and 5caC (mispaired with G (blue) or A (red)) oligonucleotides were treated with indicated enzymes for 1 hour at 37°C, and subsequently treated with hyTDG-lyase (Y163K) or NaOH to break the DNA backbone at the abasic site, mixed with an equal volume of formamide, and separated in 20% polyacrylamide denaturing gel. Gels were visualized using a STORM gel imager. Data presented as average value ± SD (n = 3).

We have for the first time performed a systematic study of the activities of the four human glycosylases in uracil DNA glycosylase superfamily on the complete set of pyrimidines that can arise from oxidation and deamination of 5mC. Data are presented graphically in Fig 7. Studies with synthetic oligonucleotides containing modified bases allow direct observation of the activities of glycosylases. While the synthesis of oligonucleotides containing normal DNA bases is routine, the inclusion of modified bases that require additional selective protection, or are chemically labile, requires more challenging chemistry.

Our laboratory has previously developed a synthetic method to incorporate 5hmU into DNA in 1993 [47], and subsequently 5hmC in 1997 [48]. The availability of these analogs allowed studies of DNA-protein interactions and glycosylase activities. The discovery of the TET enzymes published in 2009 [39] drove a resurgence in synthetic activity and currently, phosphoramidites for the synthesis of 5hmC, 5foC, 5caC and 5hmU are commercially available. Phosphoramidites for 5foU and 5caU [56–59] have been developed but are not currently available commercially and were synthesized herein.

Among the glycosylases examined here, hUNG2 has the most limited substrate range. Uracil is efficiently removed from DNA by hUNG2, when paired with either A or G, and even in single-stranded DNA [74–76]. hUNG2 functions to remove uracil resulting from deamination of cytosine (U:G). It also removes uracil in a U:A base pair resulting from the misincorporation of dUMP during DNA replication or repair synthesis. While U:A is not mutagenic, uracil in DNA paired with A can interfere with sequence-specific DNA protein interactions [77], possibly explaining the role for repair of U:A. Prior structural studies have shown that a tyrosine residue in the pyrimidine binding pocket prevents hUNG2 from excising pyrimidines with 5-substituents larger than hydrogen or fluorine atoms [76]. Additionally, a strategically placed asparagine residue, locked in place by a network of water molecules, distinguishes U from C analogs [75]. We confirm here that hUNG2 does not remove any of the U or C analogs with oxidized 5-methyl groups. While hUNG2 has an important role in protecting DNA from endogenous DNA damage, it is unlikely involved in epigenetic reprogramming.

hSMUG1 [61, 78–81] was initially identified as a single-strand selective uracil glycosylase [61]. However, subsequent studies examining buffer conditions revealed it to be active on duplex DNA [78]. hSMUG1 does not remove T from a T:G mispair. Previously, hSMUG1 was shown to remove 5hmU, 5foU and 5caU when mispaired with G and 5hmU and 5foU when paired with A [79–81]. We show here that hSMUG1 also removes 5caU when paired with A. Surprisingly, 5caU is removed as efficiently as U when paired with A or G. Previous structural studies have suggested the presence of a water molecule within the catalytic pocket of hSMUG1 that would block entry of the T-methyl group [78–81]. Alternatively, the water molecule could be displaced by polar substituents such as 5hmU, 5foU and 5caU which could hydrogen bond with amides previously holding the water molecule. Collectively, these results suggest that an important endogenous function of hSMUG1 is the removal of oxidation damage products derived from T. As hSMUG1 also repairs these damaged bases when paired with G, it may also play a role in epigenetic reprogramming.

**Fig 8:**
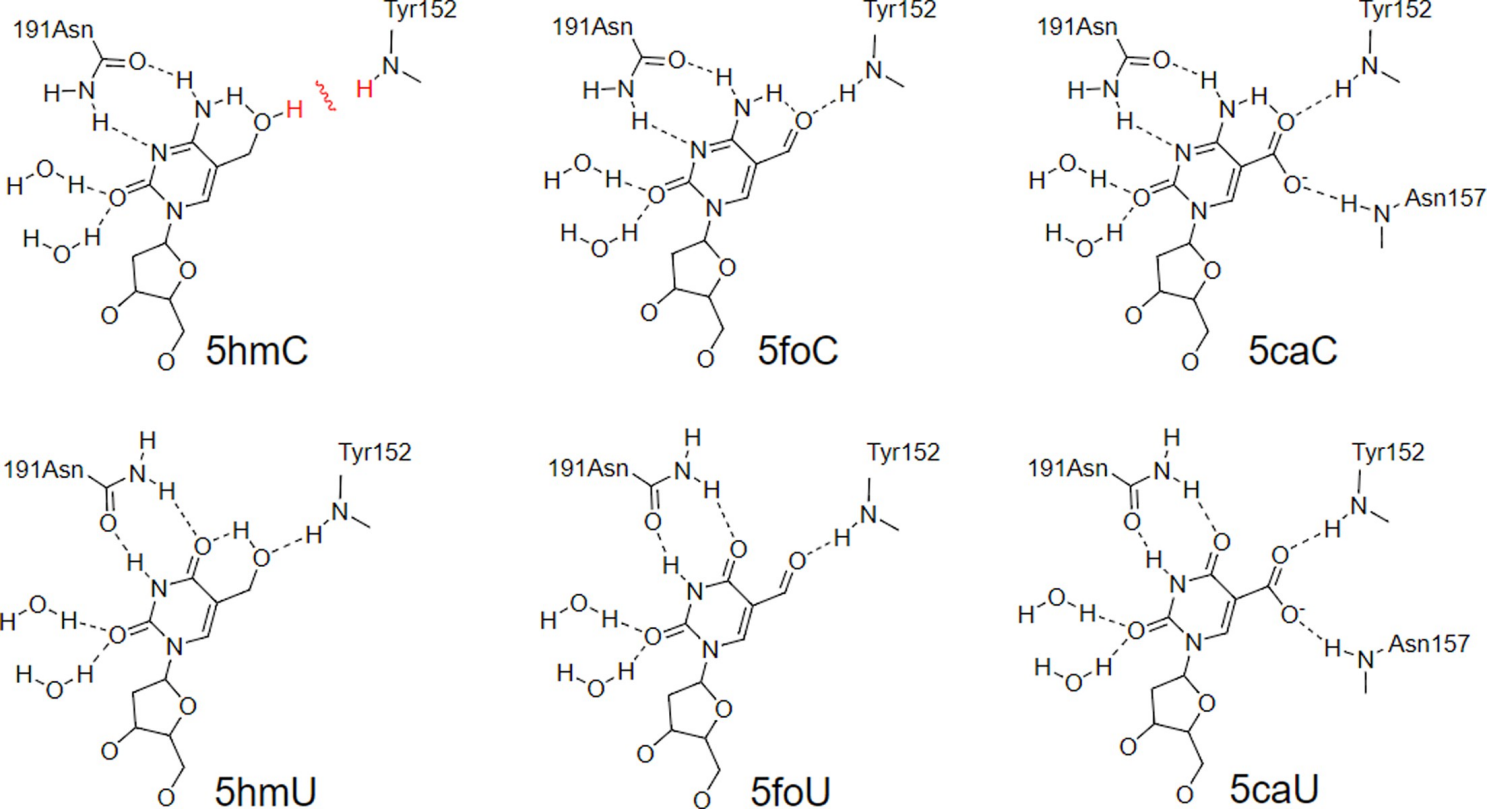
Potential hydrogen bonding interactions in the catalytic pocket of hTDG that allows recognition of 5foC, 5caC, 5hmU, 5foU, and 5caU, but not 5hmC.

hTDG has been shown to excise both 5foC and 5caC, but not 5hmC, when paired with G as previously described [46, 69, 72, 82–84]. It is surprising that hTDG shows activity against all the thymine oxidation analogs, including 5hmU, the cytosine oxidation analogs 5foC and 5caC, but not 5hmC. High resolution crystal structures reveal that the active site of hTDG with a substrate bound demonstrate various specific DNA-protein interactions (Fig 8) [69]. Importantly an asparagine (N191) can hydrogen bond with the pyrimidine ring and the amide backbone of tyrosine 152 (Y152) can also potentially hydrogen bond with the 5-substituent. However, 5hmC due to an internal hydrogen bond could potentially clash with the hydrogen on Y152. This may explain the preference for 5hmU rather than 5hmC.

Prior studies have also shown that hTDG preferentially cleaves analogs opposite G. It was therefore surprising to see that hTDG cleaves 5foC and 5caC opposite A with similar efficiency. The excision of 5foC and 5caC by hTDG is in accord with its participation in epigenetic reprogramming.

In accord with our own results, hTDG has been shown to remove mispaired uracil efficiently; however, its activity on mispaired thymine is remarkably weak. hTDG has also been reported to remove mispaired 5hmU as well as mispaired 5foU, but not as efficiently as mispaired U. Unlike the other U analogs, 5foU is more chemically labile. A distinct cleaved band is frequently observed in the control lane, especially when alkali and heat are used to cleave the DNA phosphate backbone. The chemical lability of 5foU would make it a poor epigenetic signal.

The methyl binding domain-4 protein (hMBD4) has two domains. A methyl binding domain on the N-terminus and a glycosylase activity on the C-terminus [85–90]. hMBD4 is the second of two glycosylases with T:G activity; however, like TDG, the T:G activity is relatively weak. Also like hTDG, hMBD4 can excise the oxidized T analogs when paired with G. The presence of the methyl binding domain has been suggested to localize hMBD4 to methylated CpG dinucleotides to promote repair of 5mC adducts.

The most surprising finding from this study is that 5caU is a very good substrate for three of the glycosylases examined here. It is the best substrate for hSMUG1 and hTDG, and third best for hMBD4. Mispaired T is excised by hTDG and hMBD4, but it is the worst substrate of the uracil analogs examined. Upon the basis of these findings, the primary function of hTDG and hMBD4 appears to be the removal of the oxidized T analogs, and that T removal seems almost to be an incidental activity.

### 5-Carboxyuracil is found *in vivo*

Recently we examined the free pyrimidines in extracts from rat brain. We found high levels of 5caU, which is not an intermediate in pyrimidine synthetic pathways, that were comparable to levels of 5foC measured [65]. Furthermore, Guerniou et al. have shown repair activities for 5hmU:G, 5foU:G and 5caU:G in Hela cell extracts [91]. Coupled with the glycosylase results presented here, good evidence exists for the formation of 5caU:G in DNA and its subsequent removal by the BER pathway. However, this poses additional questions of what role it plays in the genome.

### 5mC deamination: The Achilles heel of the genome

The T:G mispair constitutes an Achilles heel for the human genome. The deamination of 5mC:G to T:G is responsible for a disproportionate number of transition mutations found in human tumors. Why then is T:G so poorly repaired? The discovery of uracil glycosylase by Lindahl and coworkers was rationalized by the need to remove U:G, which would result in a mutation if not repaired. The deamination of 5mC in DNA is only slightly faster than C, but generates a slowly repaired T:G mispair [92]. Although the T:G mispair could also be repaired by the mismatch repair pathway [93], mutations at methylated CpG dinucleotides appear as mutational hotspots in human tumors, which has been explained by the poor repair of T:G [31]. Our results demonstrate that the capacity of enzymes attributed to T:G repair *in vivo*, have instead been better optimized to repair analogs with polar 5-substituents, which would diminish the repair of T:G.

The formation of 5hmU:G, 5foU:G and 5caU:G mispairs would require the oxidation of 5mC and deamination, or deamination followed by oxidation (Fig 1). The formation of these analogs by endogenous chemical pathways is unlikely as we have discussed previously [51]. Alternatively, enzymatic pathways have been proposed. The enzymatic deamination of cytosine and cytosine analogs in DNA has been demonstrated. However, the known deaminases do not function efficiently on the oxidized 5mC analogs [55]. The TET enzymes can oxidize 5mC to 5caC in DNA, but they appear to act predominantly on 5mC and not T. Although enzyme activities that oxidize T are known [94], only weak T oxidation has been demonstrated for TET family enzymes or in mammalian cells [95, 96].

Pathways for the formation of 5caU:G in DNA remain an enigma although the glycosylases proposed to be involved in epigenetic reprogramming have robust 5caU:G activity, Hela cells extracts have abundant 5caU:G excision activity, and the resulting glycosylase product, 5caU, has been measured in rat brain at significant levels. The oxidation of 5mC to 5foC and 5caC, followed by hTDG removal represents an active demethylation pathway supported by results from enzymatic assays on TET oxidases and hTDG. Perhaps the capacity of hTDG to remove 5foC and 5caC when paired with G, as well as 5foU and 5caU when paired with G, provides some backup to inadvertent deamination of intermediates in epigenetic reprogramming.

### Intersection of DNA damage, repair and epigenetic reprogramming

The results of our studies generate many additional questions on mechanisms of T:G repair and the intersection of DNA damage, repair and epigenetic reprogramming. For example, it is intriguing to hypothesize that the role of enzymatic repair of deaminated and oxidized pyrimidines *in vivo* could be to reduce the incidence of transition mutations by converting a poorly repaired substrate, a T:G mispair, into several significantly better substrates. However, it is unclear if the formation of the oxidized uracil analogs occurs by either spontaneous and or enzymatic pathways. In addition, while in replicating cells a T:G mispair could lead to a C>T transition mutation, this would not be the case in post-mitotic cells, such as neurons. Interestingly, MBD proteins do not appear to discriminate between 5mC:G or T:G mispair [20]. Therefore, it begs the question if repair of the oxidation products of 5mC deamination could also serve to preserve DNA-protein interactions that could be critical to proper cellular function or serve as epigenetic markers in their own right.

## Supporting information

S1 File (DOCX)

S1 Raw images (PDF)
