# Supplementary material for: Chemical and enzymatic modifications of 5-methylcytosine at the intersection of DNA damage, repair, and epigenetic reprogramming
Source: PLoS One. 2022 Aug 29;17(8):e0273509. doi: 10.1371/journal.pone.0273509 (PMC9423628; doi:10.1371/journal.pone.0273509)
Supplement: S1 File — (DOCX) [file pone.0273509.s001.docx]

**Table of Contents**

**Table S1. Pyrimidine GC-MS retention times and masses.**

**Figure S1. The excision specificity of human uracil family glycosylases for a series of 5-substituted uracil analogs paired with G (left) or A (right) (repeat 2)**.

**Figure S2. The excision specificity of human uracil family glycosylases for a series of 5-substituted uracil analogs paired with G (left) or A (right)** **(repeat 3).**

**Figure S3. The excision specificity of human uracil family glycosylases for a series of 5-substituted cytosine analogs paired with G (left) or A (right) (repeat 2)**.

**Figure S4. The excision specificity of human uracil family glycosylases for a series of 5-substituted cytosine analogs paired with G (left) or A (right) (repeat 3)**.

**Figure S5. hSMUG1 excises U from a U:G-containing oligonucleotide as verified by GC-MS.**

**Figure S6. hSMUG1 excises 5hmU from a 5hmU:G-containing oligonucleotide as verified by GC-MS.**

**Figure S7. hSMUG1 excises 5foU from a 5foU:G-containing oligonucleotide as verified by GC-MS.**

**Figure S8. hSMUG1 excises 5caU from a 5caU:G-containing oligonucleotide as verified by GC-MS.**

**Figure S9. hSMUG1 excises U from a U:A-containing oligonucleotide as verified by GC-MS.**

**Figure S10. hSMUG1 excises 5hmU from a 5hmU:A-containing oligonucleotide as verified by GC-MS.**

**Figure S11. hSMUG1 excises 5foU from a 5foU:A-containing oligonucleotide as verified by GC-MS.**

**Figure S12. hSMUG1 excises 5caU from a 5caU:A-containing oligonucleotide as verified by GC-MS.**

**Figure S13. hMBD4 excises U from a U:G-containing oligonucleotide as verified by GC-MS.**

**Figure S14. hMBD4 excises T from a T:G-containing oligonucleotide as verified by GC-MS.**

**Figure S15. hMBD4 excises 5hmU from a 5hmU:G-containing oligonucleotide as verified by GC-MS.**

**Figure S16. hMBD4 excises 5foU from a 5foU:G-containing oligonucleotide as verified by GC-MS.**

**Figure S17. hMBD4 excises 5caU from a 5caU:G-containing oligonucleotide as verified by GC-MS.**

**Figure S18. hMBD4 excises U from a U:A-containing oligonucleotide as verified by GC-MS.**

**Figure S19. hTDG excises U from a U:G-containing oligonucleotide as verified by GC-MS.**

**Figure S20. hTDG excises T from a T:G-containing oligonucleotide as verified by GC-MS.**

**Figure S21. hTDG excises 5hmU from a 5hmU:G-containing oligonucleotide as verified by GC-MS.**

**Figure S22. hTDG excises 5foU from a 5foU:G-containing oligonucleotide as verified by GC-MS.**

**Figure S23. hTDG excises 5caU from a 5caU:G-containing oligonucleotide as verified by GC-MS.**

**Figure S24. hTDG excises 5foC from a 5foC:G-containing oligonucleotide as verified by GC-MS.**

**Figure S25. hTDG excises 5caC from a 5caC:G-containing oligonucleotide as verified by GC-MS.**

**Figure S26. hTDG excises U from a U:A-containing oligonucleotide as verified by GC-MS.**

**Figure S27. hTDG excises 5foC from a 5foC:A-containing oligonucleotide as verified by GC-MS.**

**Figure S28. hTDG excises 5caC from a 5caC:A-containing oligonucleotide as verified by GC-MS.**

**
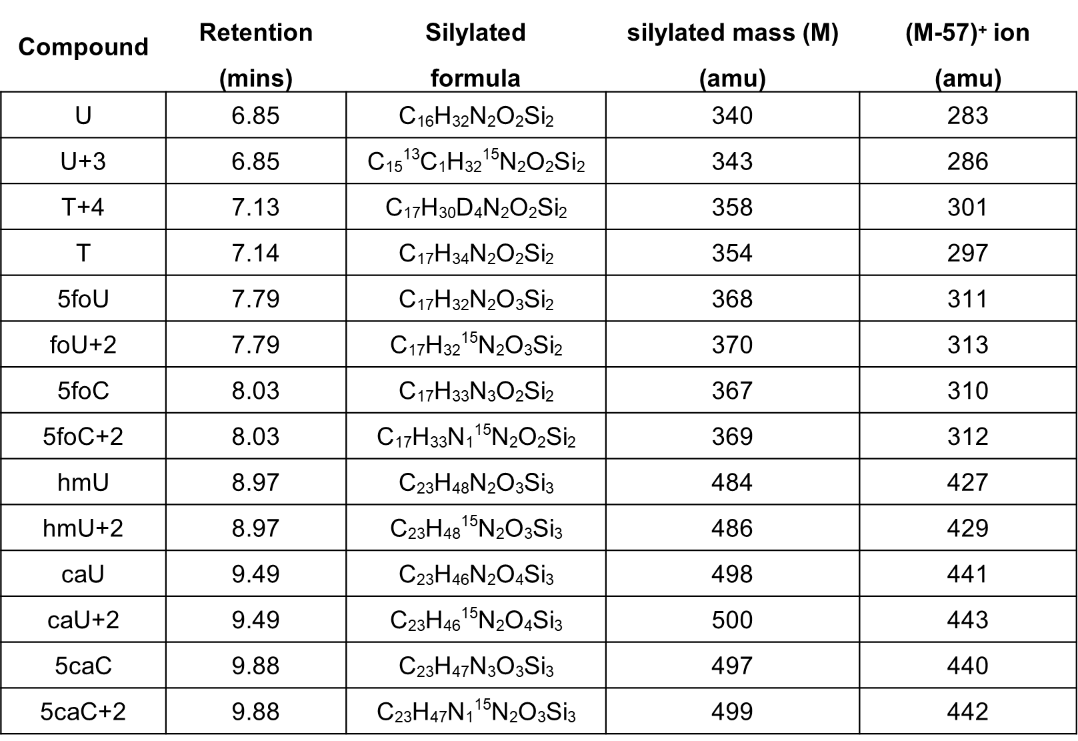
**

**Table S1. GC-MS retention times and masses of silylated pyrimidine derivative ions.**


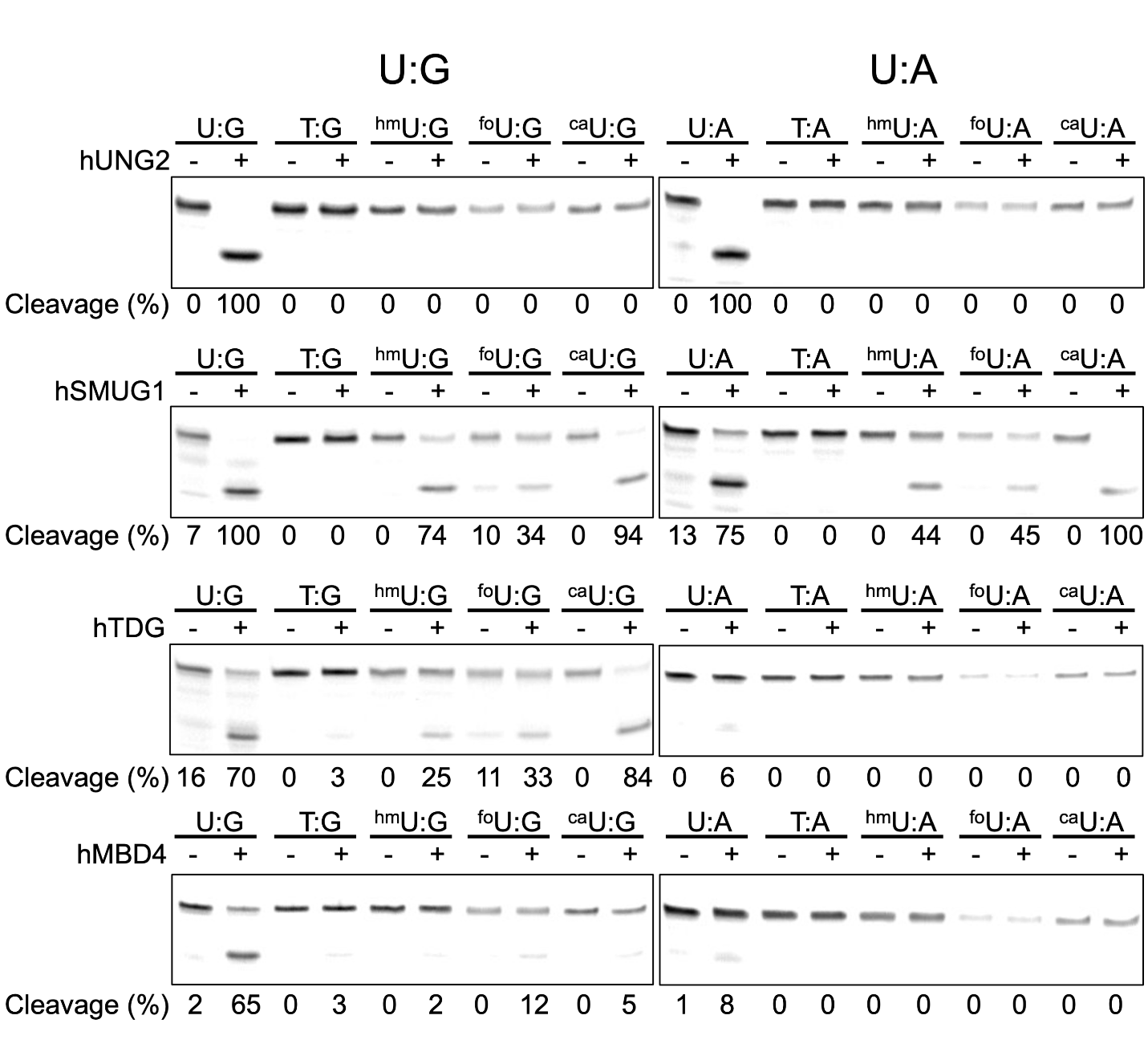


**Figure S1. The excision specificity of human uracil family glycosylases for a series of 5-substituted uracil analogs paired with G (left) or A (right)**. FAM-labelled oligonucleotide duplexes (2.5 pmol) in buffer appropriate for each enzyme were incubated with a glycosylase at 37 ^o^C for 1 h. Duplexes paired with G were then incubated with hyTDG lyase (Y163K) (65^o^C, 1 h) and those paired with A incubated with NaOH solution (96^o^C, 10 min) to cleave the abasic site. Oligonucleotides were resolved on a 20% polyacrylamide/urea gel, visualized on a STORM imager, and quantified with “ImageJ” software. The amount of cleavage is shown below the lanes in each gel. Some artifact cleavage of 5foU-containing oligonucleotides is observed due to its thermal lability (repeat 2).


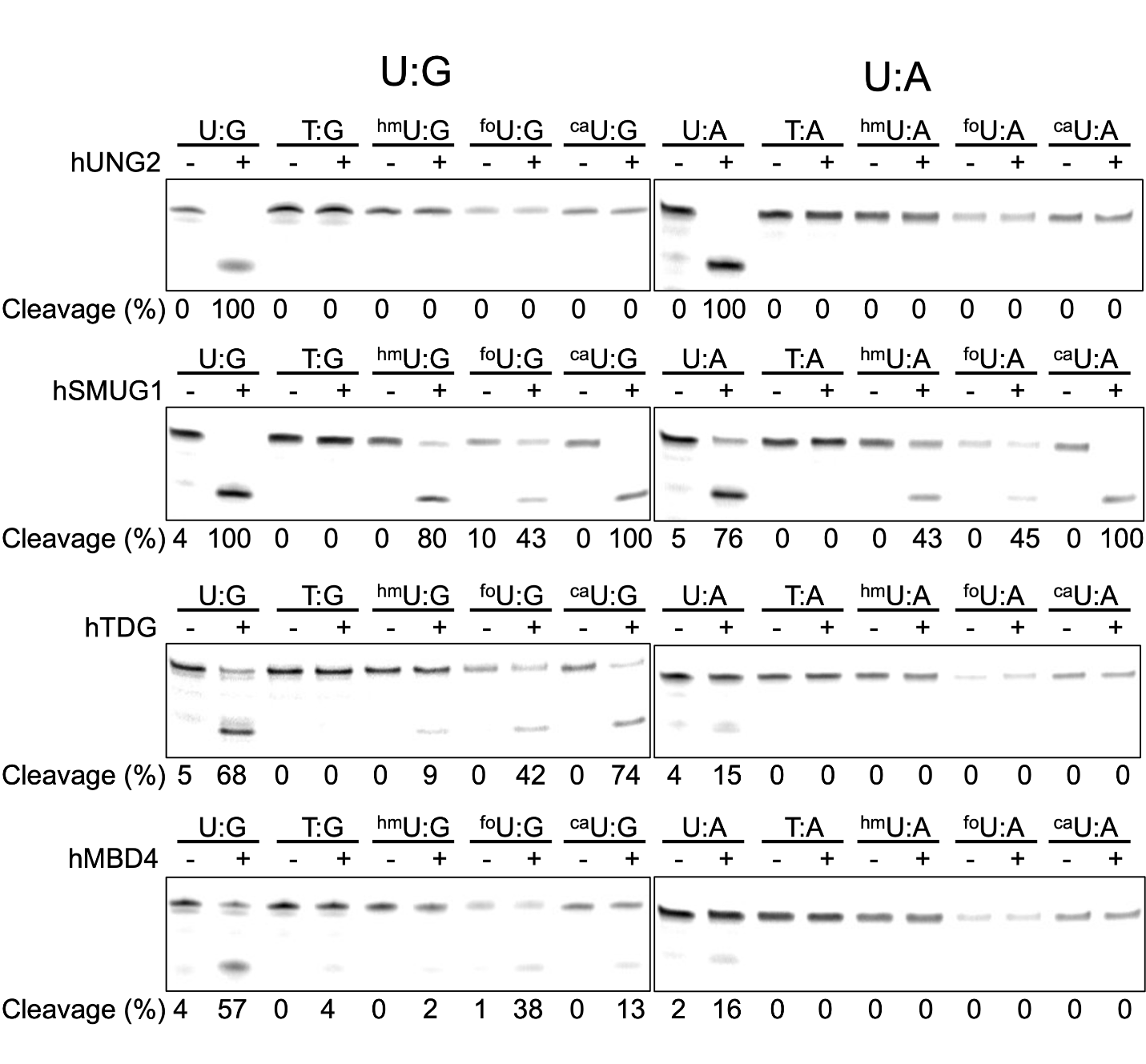


**Figure. S2. The excision specificity of human uracil family glycosylases for a series of 5-substituted uracil analogs paired with G (left) or A (right)**. FAM-labelled oligonucleotide duplexes (2.5 pmol) in buffer appropriate for each enzyme were incubated with a glycosylase at 37 ^o^C for 1 h. Duplexes paired with G were then incubated with hyTDG lyase (Y163K) (65^o^C, 1 h) and those paired with A incubated with NaOH solution (96^o^C, 10 min) to cleave the abasic site. Oligonucleotides were resolved on a 20% polyacrylamide/urea gel, visualized on a STORM imager, and quantified with “ImageJ” software. The amount of cleavage is shown below the lanes in each gel. Some artifact cleavage of 5foU-containing oligonucleotides is observed due to its thermal lability (repeat 3).


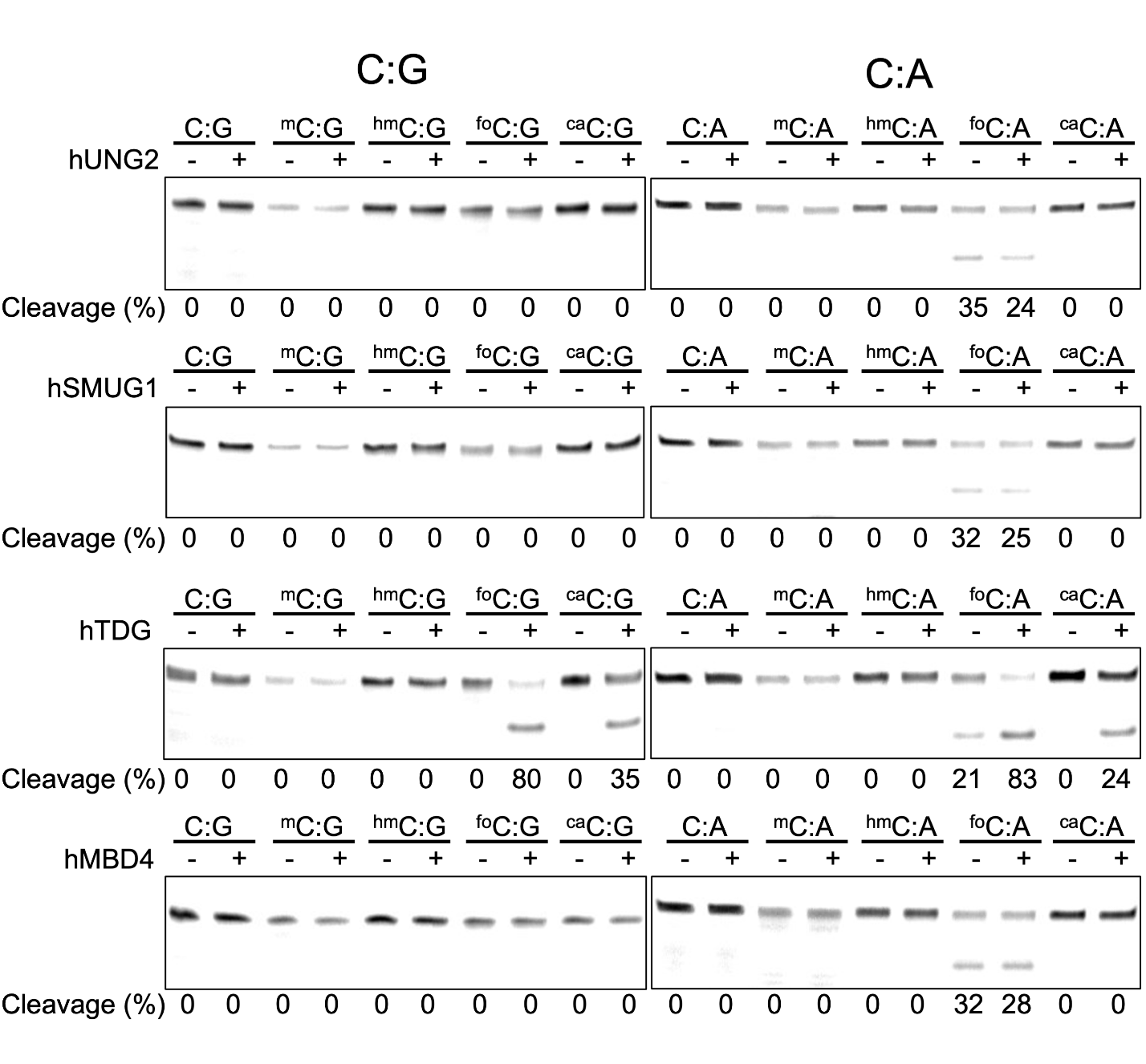


**Figure S3. The excision specificity of human uracil family glycosylases for a series of 5-substituted cytosine analogs paired with G (left) or A (right)**. FAM-labelled oligonucleotide duplexes (2.5 pmol) in buffer appropriate for each enzyme were incubated with a glycosylase at 37 ^o^C for 1 h. Duplexes paired with G were then incubated with hyTDG lyase (Y163K) (65^o^C, 1 h) and those paired with A incubated with NaOH solution (96^o^C, 10 min) to cleave the abasic site. Oligonucleotides were resolved on a 20% polyacrylamide/urea gel, visualized on a STORM imager, and quantified with “ImageJ” software. The amount of cleavage is shown below the lanes in each gel. Some artifact cleavage of 5foC-containing oligonucleotides is observed due to its alkaline lability (repeat 2).


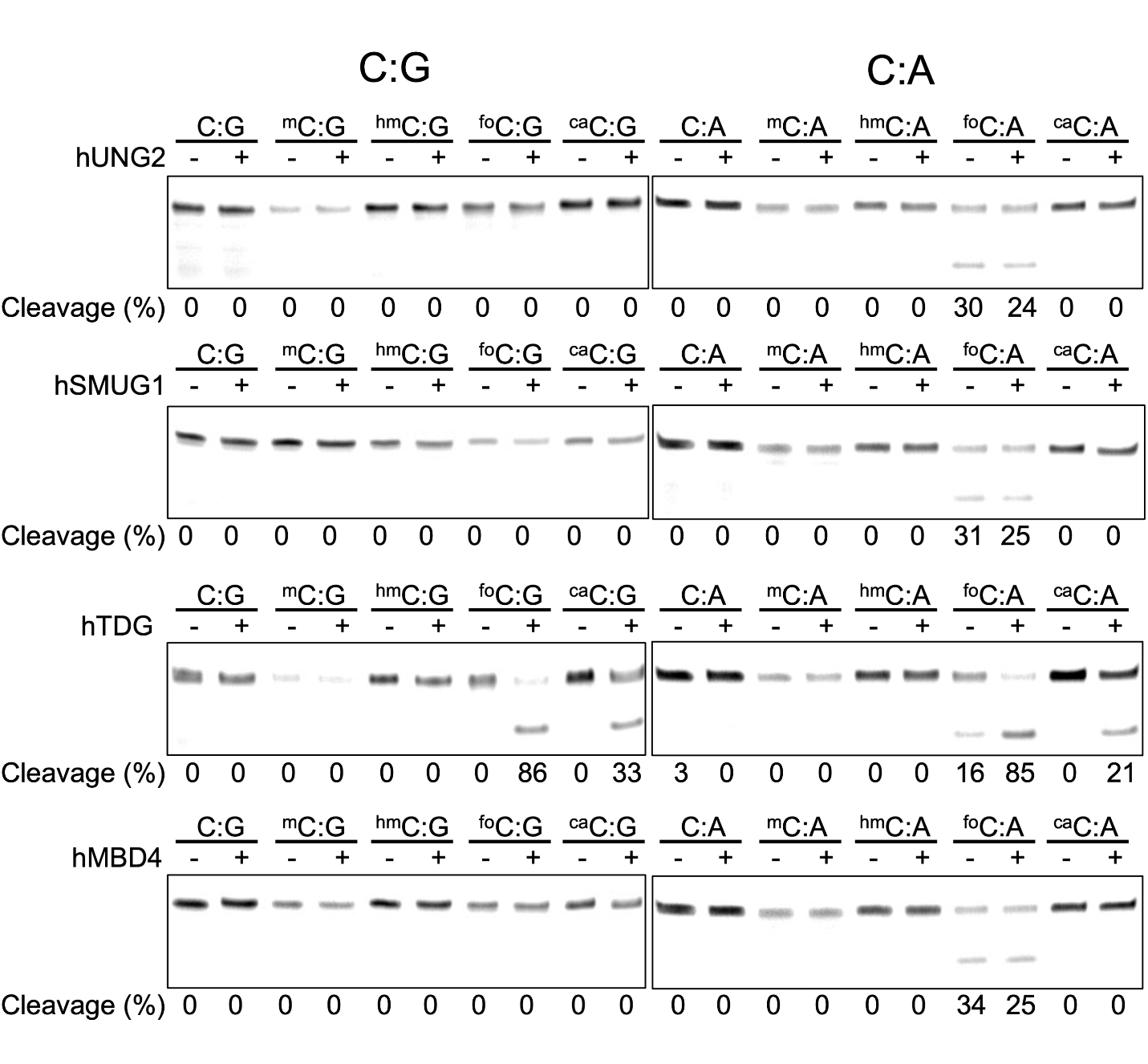
**Figure. S4. The excision specificity of human uracil family glycosylases for a series of 5-substituted cytosine analogs paired with G (left) or A (right)**. FAM-labelled oligonucleotide duplexes (2.5 pmol) in buffer appropriate for each enzyme were incubated with a glycosylase at 37 ^o^C for 1 h. Duplexes paired with G were then incubated with hyTDG lyase (Y163K) (65^o^C, 1 h) and those paired with A incubated with NaOH solution (96^o^C, 10 min) to cleave the abasic site. Oligonucleotides were resolved on a 20% polyacrylamide/urea gel, visualized on a STORM imager, and quantified with “ImageJ” software. The amount of cleavage is shown below the lanes in each gel. Some artifact cleavage of 5foC-containing oligonucleotides is observed due to its alkaline lability (repeat 3).


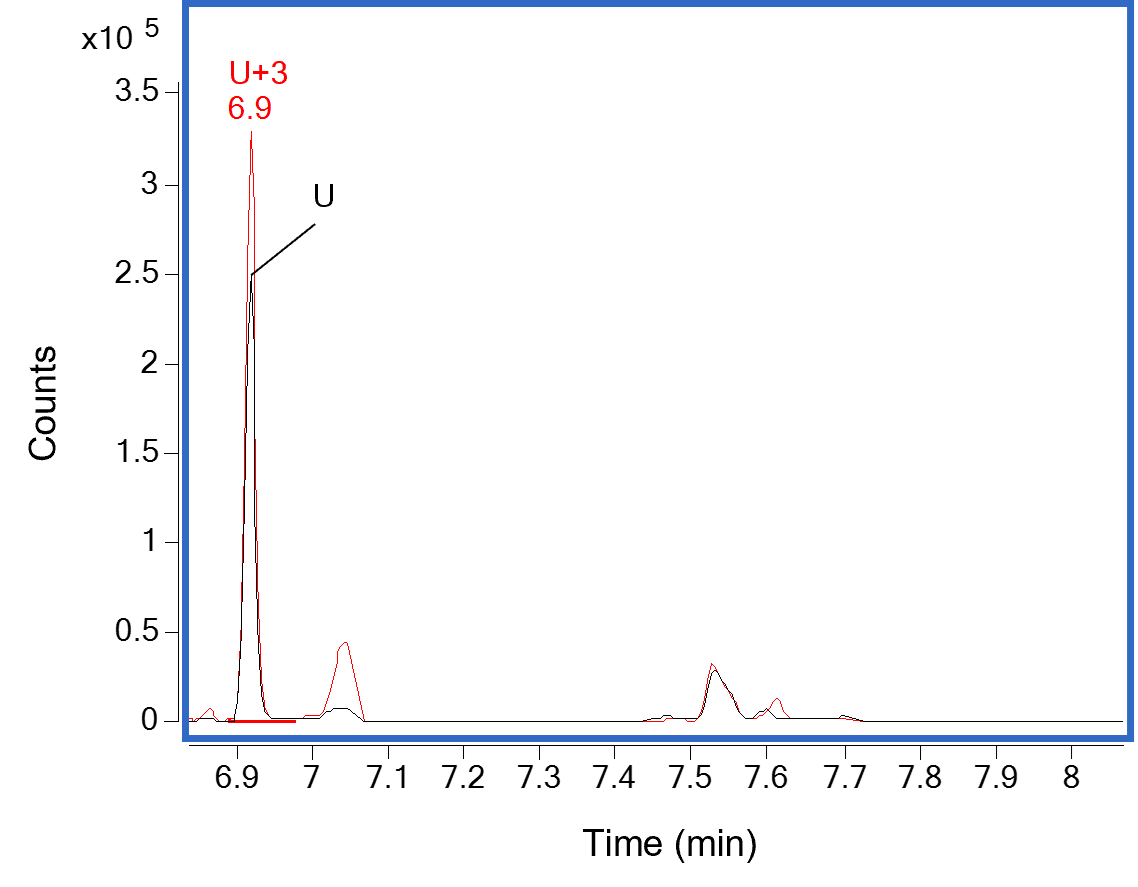


**Figure S5. hSMUG1 excises U from a U:G-containing oligonucleotide as verified by GC-MS.** A duplex oligonucleotide containing a U:G mispair (100 pmol) was prepared in buffer containing 10 mM potassium phosphate pH 7.7, 30 mM sodium chloride and 40 mM potassium chloride and 100 pmol of a U+3 stable isotope internal standard. The oligonucleotides and free base standard were incubated with hSMUG1 (81 pmoles) at 37 ^o^C for 2 h in a 100 mL reaction volume. Free bases were separated from enzymes and oligonucleotides by spin filtration, dried under reduced pressure and derivatized to TBDMS derivatives. Derivatized free bases were separated by GC and identified by MS with selected ion monitoring. The black chromatogram above (*m/z* 283) corresponds to the U released by hSMUG1 from the U:G-containing oligonucleotide duplex and the red chromatogram (*m/z* 286) corresponds to the isotope enriched internal standard (U+3). Identification of U release is confirmed by coelution of U and U+3 detected at the appropriates values of *m/z*.


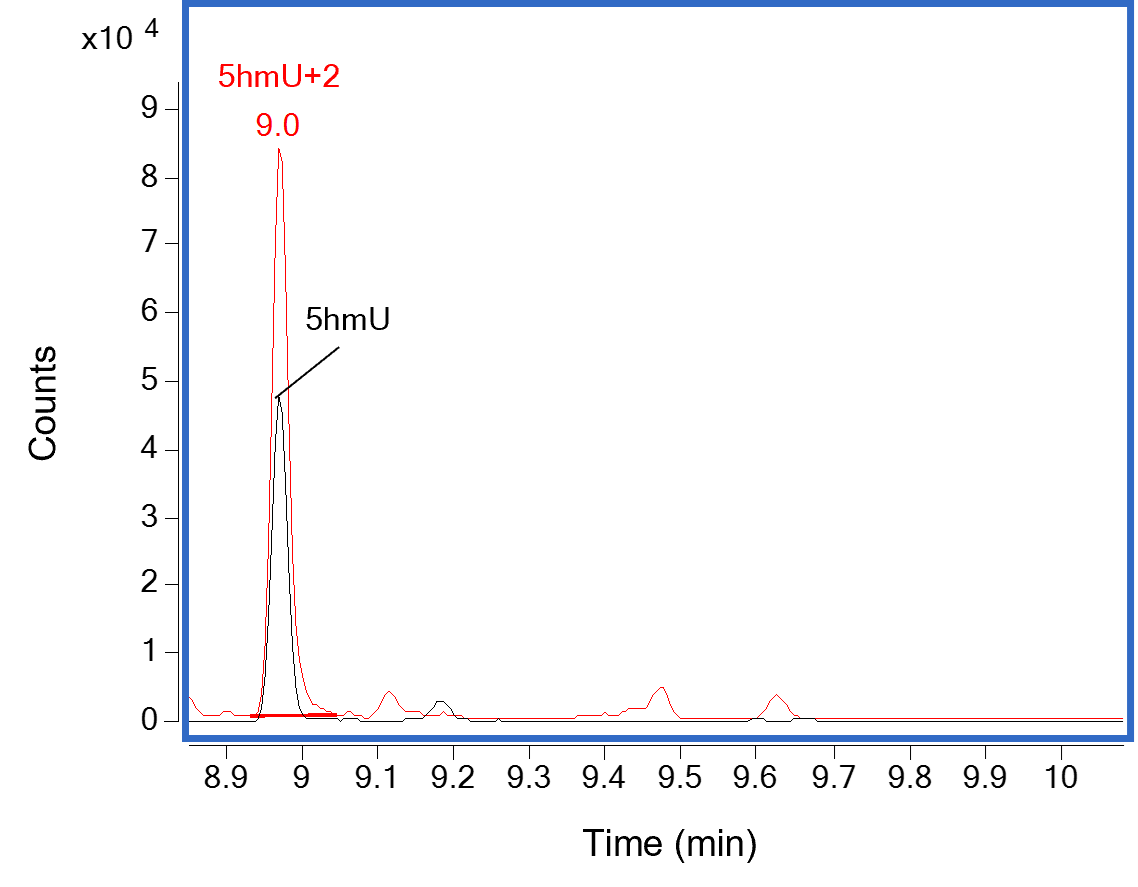


**Figure S6. hSMUG1 excises 5hmU from a 5hmU:G-containing oligonucleotide as verified by GC-MS.** A duplex oligonucleotide containing a 5hmU:G mispair (100 pmol) was prepared in buffer containing 10 mM potassium phosphate pH 7.7, 30 mM sodium chloride and 40 mM potassium chloride and 100 pmol of a 5hmU+2 stable isotope internal standard. The oligonucleotides and free base standard were incubated with hSMUG1 (81 pmoles) at 37 ^o^C for 2 h in a 100 mL reaction volume. Free bases were separated from enzymes and oligonucleotides by spin filtration, dried under reduced pressure and derivatized to TBDMS derivatives. Derivatized free bases were separated by GC and identified by MS with selected ion monitoring. The black chromatogram above (*m/z* 427) corresponds to the 5hmuU released by hSMUG1 from the 5hmU:G-containing oligonucleotide duplex and the red chromatogram (*m/z* 429) corresponds to the isotope enriched internal standard (5hmU+2). Identification of 5hmU release is confirmed by coelution of 5hmU and 5hmU+2 detected at the appropriates values of *m/z*.

**
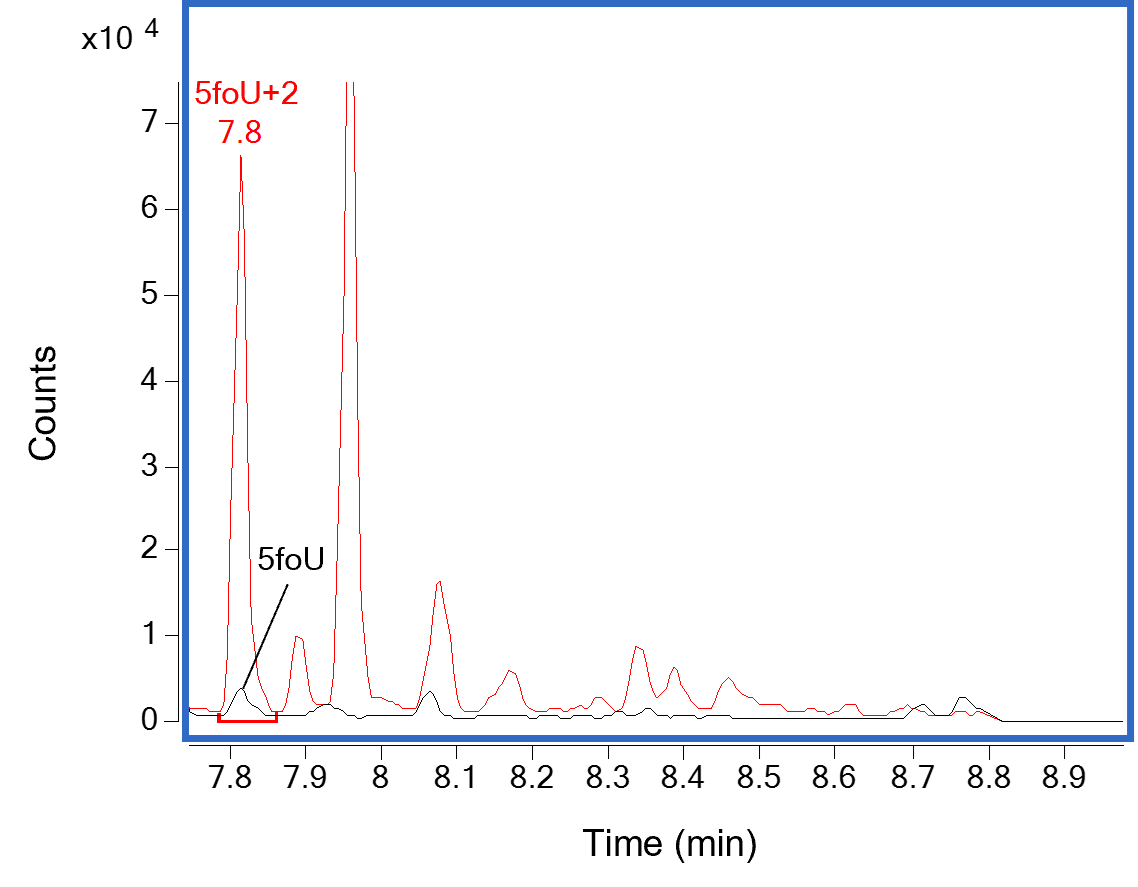
**

**Figure S7. hSMUG1 excises 5foU from a 5foU:G-containing oligonucleotide as verified by GC-MS.** A duplex oligonucleotide containing a 5foU:G mispair (100 pmol) was prepared in buffer containing 10 mM potassium phosphate pH 7.7, 30 mM sodium chloride and 40 mM potassium chloride and 100 pmol of a 5foU+2 stable isotope internal standard. The oligonucleotides and free base standard were incubated with hSMUG1 (81 pmoles) at 37 ^o^C for 2 h in a 100 mL reaction volume. Free bases were separated from enzymes and oligonucleotides by spin filtration, dried under reduced pressure and derivatized to TBDMS derivatives. Derivatized free bases were separated by GC and identified by MS with selected ion monitoring. The black chromatogram above (*m/z* 311) corresponds to the 5foU released by hSMUG1 from the 5foU:G-containing oligonucleotide duplex and the red chromatogram (*m/z* 313) corresponds to the isotope enriched internal standard (5foU+2). Identification of 5foU release is confirmed by coelution of 5foU and 5foU+2 detected at the appropriates values of *m/z*.


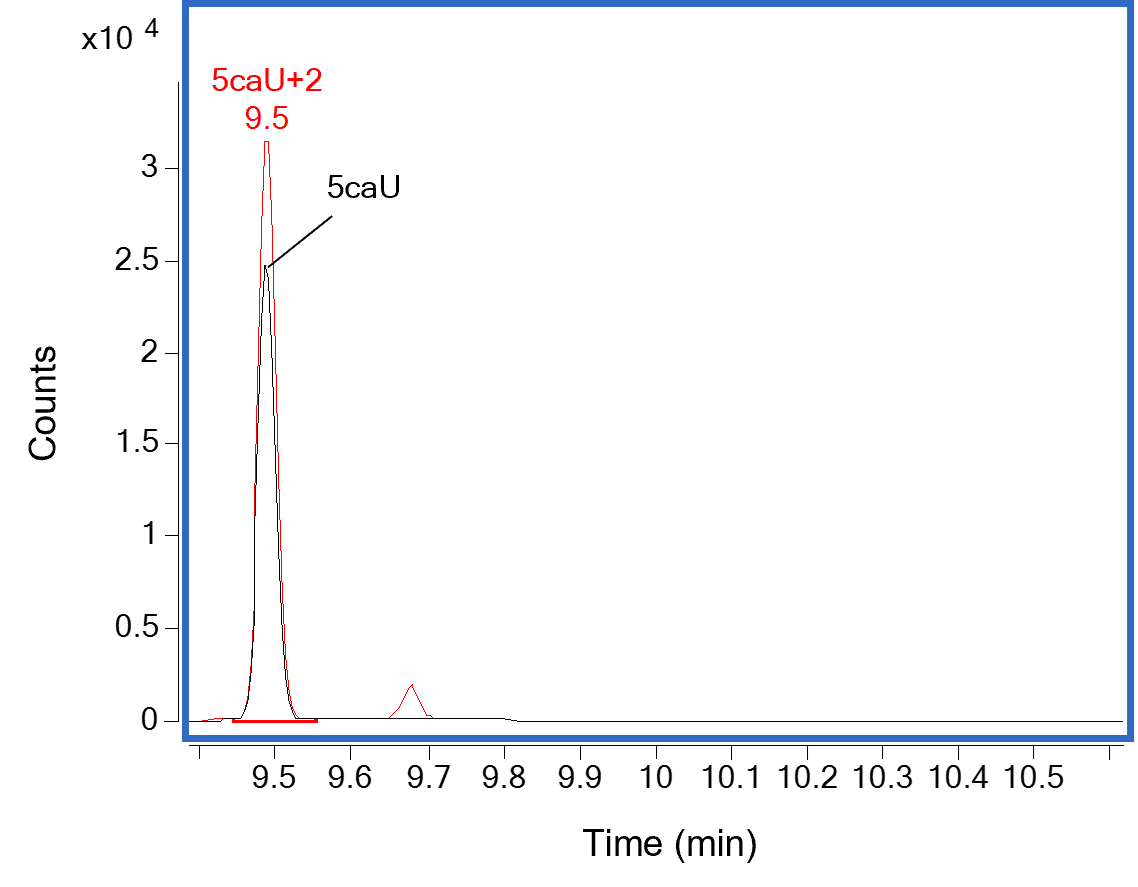


**Figure S8. hSMUG1 excises 5caU from a 5caU:G-containing oligonucleotide as verified by GC-MS.** A duplex oligonucleotide containing a 5caU:G mispair (110 pmol) was prepared in buffer containing 10 mM potassium phosphate pH 7.7, 30 mM sodium chloride and 40 mM potassium chloride and 55 pmol of a 5caU+2 stable isotope internal standard. The oligonucleotides and free base standard were incubated with hSMUG1 (89 pmoles) at 37 ^o^C for 2 h in a 110 mL reaction volume. Free bases were separated from enzymes and oligonucleotides by spin filtration, 90% of sample was dried under reduced pressure and derivatized to TBDMS derivatives. Derivatized free bases were separated by GC and identified by MS with selected ion monitoring. The black chromatogram above (*m/z* 441) corresponds to the 5caU released by hSMUG1 from the 5caU:G-containing oligonucleotide duplex and the red chromatogram (*m/z* 443) corresponds to the isotope enriched internal standard (5caU+2). Identification of 5caU release is confirmed by coelution of 5caU and 5caU+2 detected at the appropriates values of *m/z*.


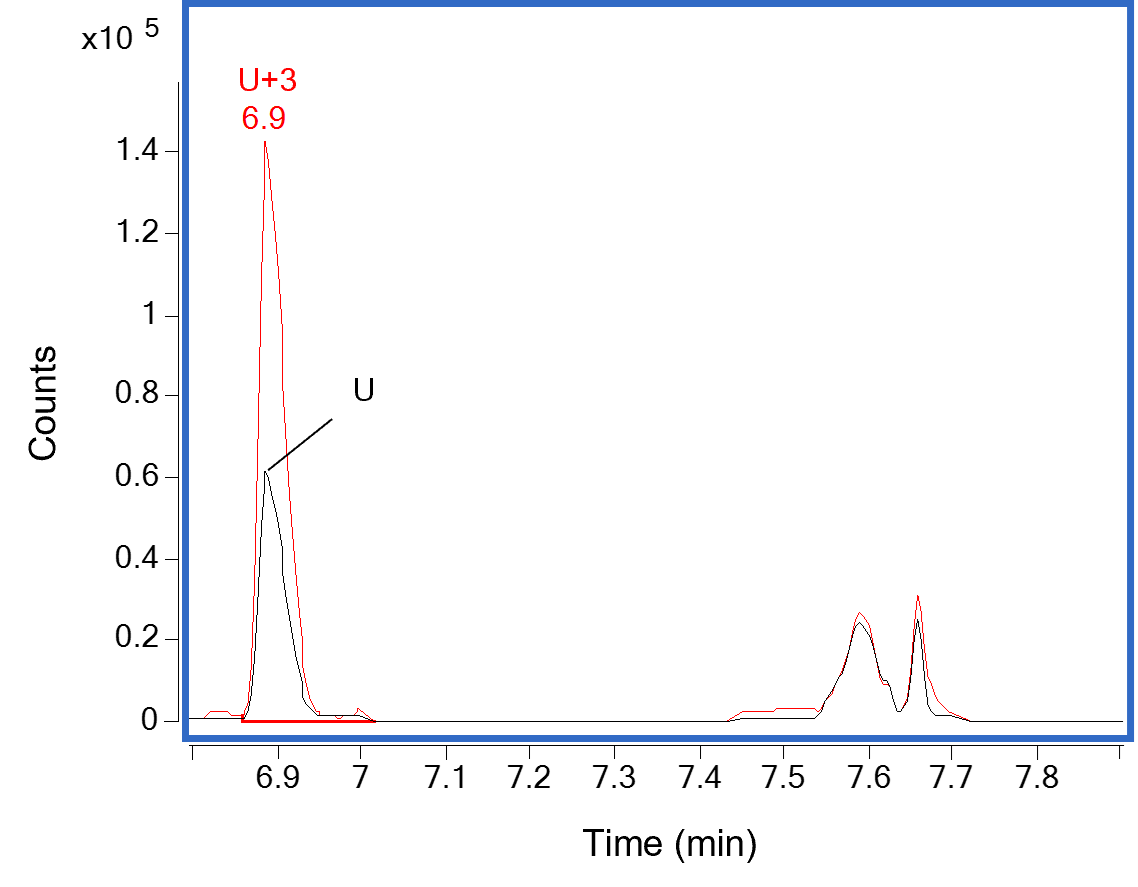


**Figure S9. hSMUG1 excises U from a U:A-containing oligonucleotide as verified by GC-MS.** A duplex oligonucleotide containing a U:A mispair (110 pmol) was prepared in buffer containing 10 mM potassium phosphate pH 7.7, 30 mM sodium chloride and 40 mM potassium chloride and 110 pmol of a U+3 stable isotope internal standard. The oligonucleotides and free base standard were incubated with hSMUG1 (89 pmoles) at 37 ^o^C for 2 h in a 110 mL reaction volume. Free bases were separated from enzymes and oligonucleotides by spin filtration, 90% of sample was dried under reduced pressure and derivatized to TBDMS derivatives. Derivatized free bases were separated by GC and identified by MS with selected ion monitoring. The black chromatogram above (*m/z* 283) corresponds to the U released by hSMUG1 from the U:A-containing oligonucleotide duplex and the red chromatogram (*m/z* 286) corresponds to the isotope enriched internal standard (U+3). Identification of U release is confirmed by coelution of U and U+3 detected at the appropriates values of *m/z*.


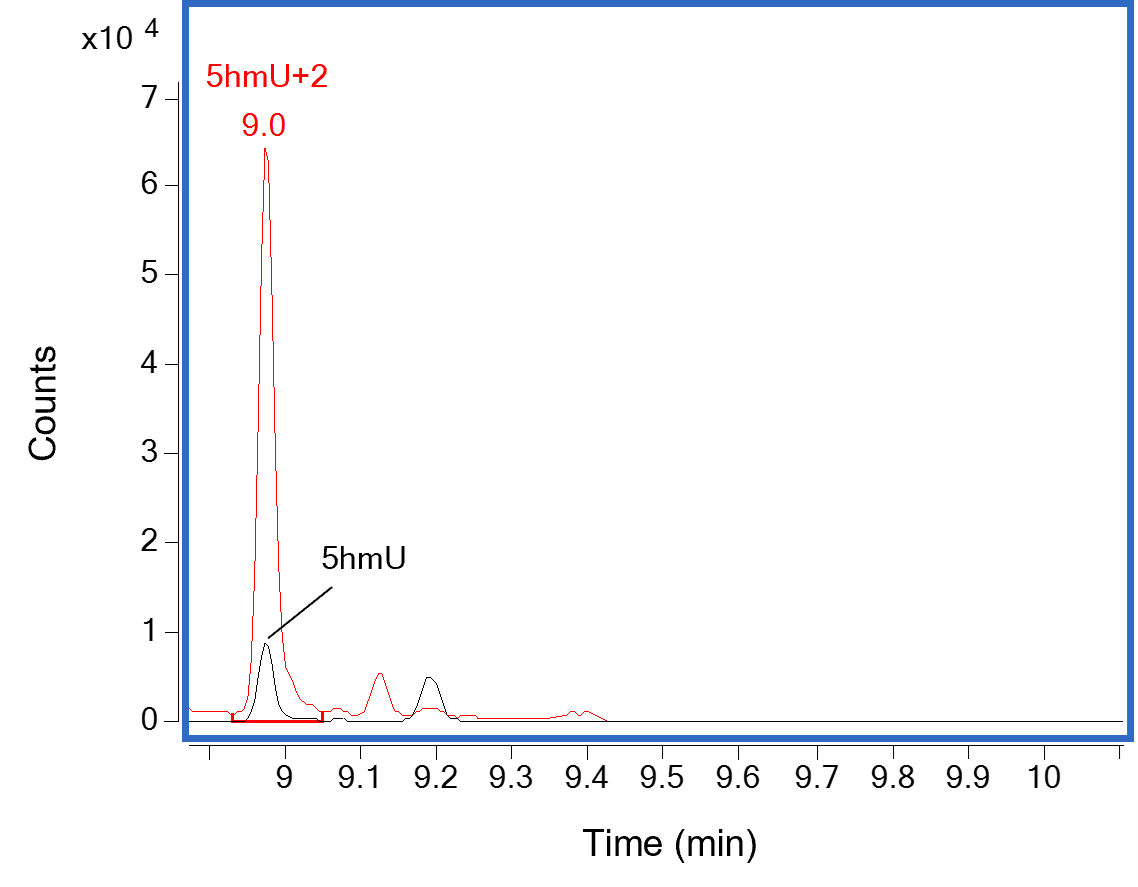


**Figure S10. hSMUG1 excises 5hmU from a 5hmU:A-containing oligonucleotide as verified by GC-MS.** A duplex oligonucleotide containing a 5hmU:A mispair (110 pmol) was prepared in buffer containing 10 mM potassium phosphate pH 7.7, 30 mM sodium chloride and 40 mM potassium chloride and 110 pmol of a 5hmU+2 stable isotope internal standard. The oligonucleotides and free base standard were incubated with hSMUG1 (89 pmoles) at 37 ^o^C for 2 h in a 110 mL reaction volume. Free bases were separated from enzymes and oligonucleotides by spin filtration, 90% of sample was dried under reduced pressure and derivatized to TBDMS derivatives. Derivatized free bases were separated by GC and identified by MS with selected ion monitoring. The black chromatogram above (*m/z* 427) corresponds to the 5hmU released by hSMUG1 from the 5hmU:A-containing oligonucleotide duplex and the red chromatogram (*m/z* 429) corresponds to the isotope enriched internal standard (5hmU+2). Identification of 5hmU release is confirmed by coelution of 5hmU and 5hmU+2 detected at the appropriates values of *m/z*.


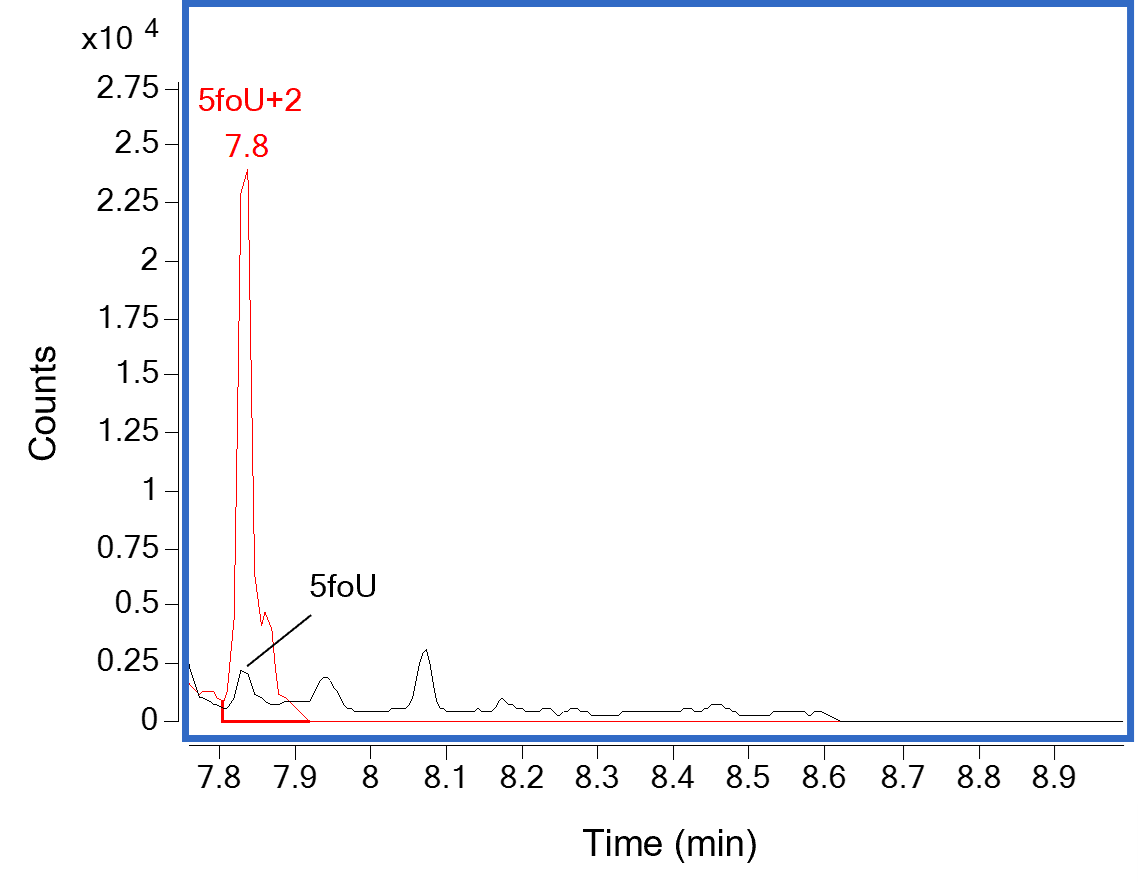


**Figure S11. hSMUG1 excises 5foU from a 5foU:A-containing oligonucleotide as verified by GC-MS.** A duplex oligonucleotide containing a 5foU:A mispair (110 pmol) was prepared in buffer containing 10 mM potassium phosphate pH 7.7, 30 mM sodium chloride and 40 mM potassium chloride and 110 pmol of a 5foU+2 stable isotope internal standard. The oligonucleotides and free base standard were incubated with hSMUG1 (89 pmoles) at 37 ^o^C for 2 h in a 110 mL reaction volume. Free bases were separated from enzymes and oligonucleotides by spin filtration, 90% of sample was dried under reduced pressure and derivatized to TBDMS derivatives. Derivatized free bases were separated by GC and identified by MS with selected ion monitoring. The black chromatogram above (*m/z* 311) corresponds to the 5foU released by hSMUG1 from the 5foU:A-containing oligonucleotide duplex and the red chromatogram (*m/z* 313) corresponds to the isotope enriched internal standard (5foU+2). Identification of 5foU release is confirmed by coelution of 5hmU and 5foU+2 detected at the appropriates values of *m/z*.


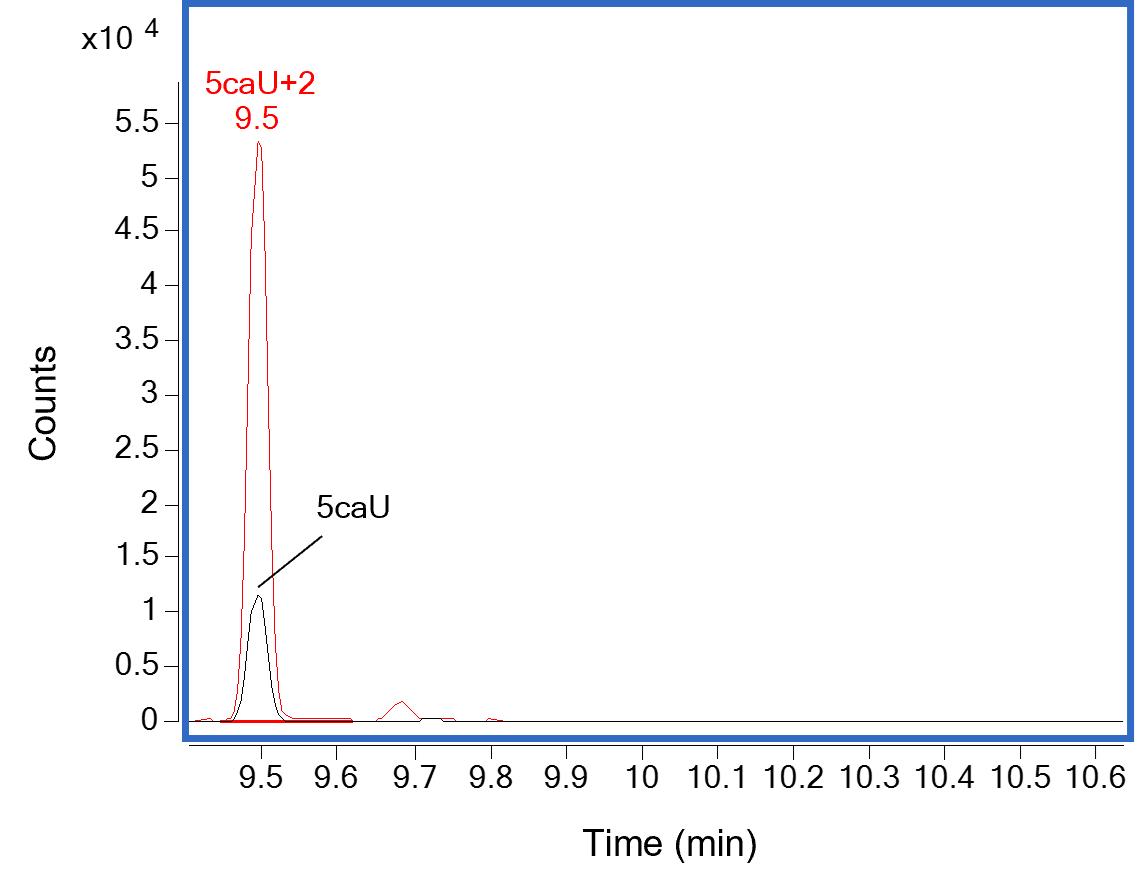


**Figure S12. hSMUG1 excises 5caU from a 5caU:A-containing oligonucleotide as verified by GC-MS.** A duplex oligonucleotide containing a 5caU:A mispair (110 pmol) was prepared in buffer containing 10 mM potassium phosphate pH 7.7, 30 mM sodium chloride and 40 mM potassium chloride and 110 pmol of a 5caU+2 stable isotope internal standard. The oligonucleotides and free base standard were incubated with hSMUG1 (89 pmoles) at 37 ^o^C for 2 h in a 110 mL reaction volume. Free bases were separated from enzymes and oligonucleotides by spin filtration, 90% of sample was dried under reduced pressure and derivatized to TBDMS derivatives. Derivatized free bases were separated by GC and identified by MS with selected ion monitoring. The black chromatogram above (*m/z* 441) corresponds to the 5CaU released by hSMUG1 from the 5caU:A-containing oligonucleotide duplex and the red chromatogram (*m/z* 443) corresponds to the isotope enriched internal standard (5caU+2). Identification of 5caU release is confirmed by coelution of 5caU and 5caU+2 detected at the appropriates values of *m/z*.


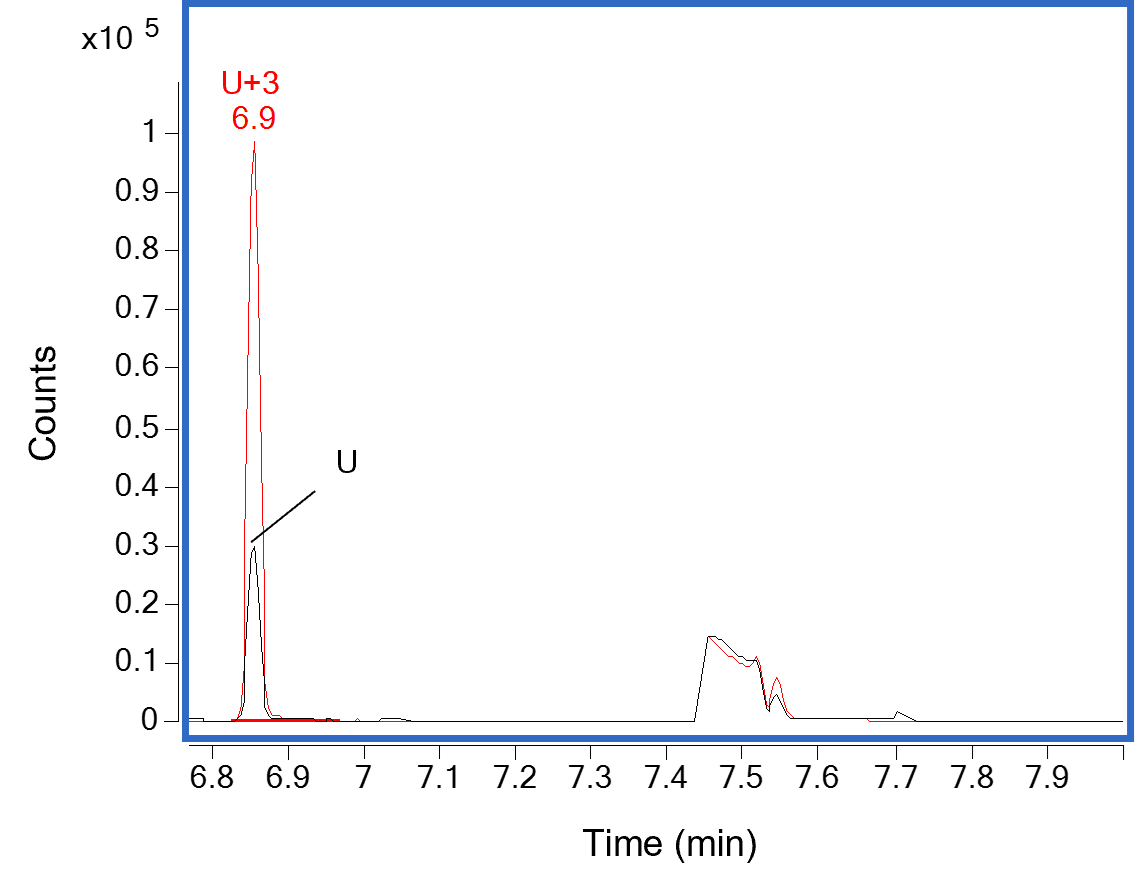


**Figure S13. hMBD4 excises U from a U:G-containing oligonucleotide as verified by GC-MS.** A duplex oligonucleotide containing a U:G mispair (110 pmol) was prepared in buffer containing 10 mM potassium phosphate pH 7.7, 30 mM sodium chloride and 40 mM potassium chloride and 110 pmol of a U+3 stable isotope internal standard. The oligonucleotides and free base standard were incubated with hMBD4 (112 pmoles) at 37 ^o^C for 2 h in a 110 mL reaction volume. Free bases were separated from enzymes and oligonucleotides by spin filtration, 90% of sample was dried under reduced pressure and derivatized to TBDMS derivatives. Derivatized free bases were separated by GC and identified by MS with selected ion monitoring. The black chromatogram above (*m/z* 283) corresponds to the U released by hMBD4 from the U:G-containing oligonucleotide duplex and the red chromatogram (*m/z* 286) corresponds to the isotope enriched internal standard (U+3). Identification of U release is confirmed by coelution of U and U+3 detected at the appropriates values of *m/z*.


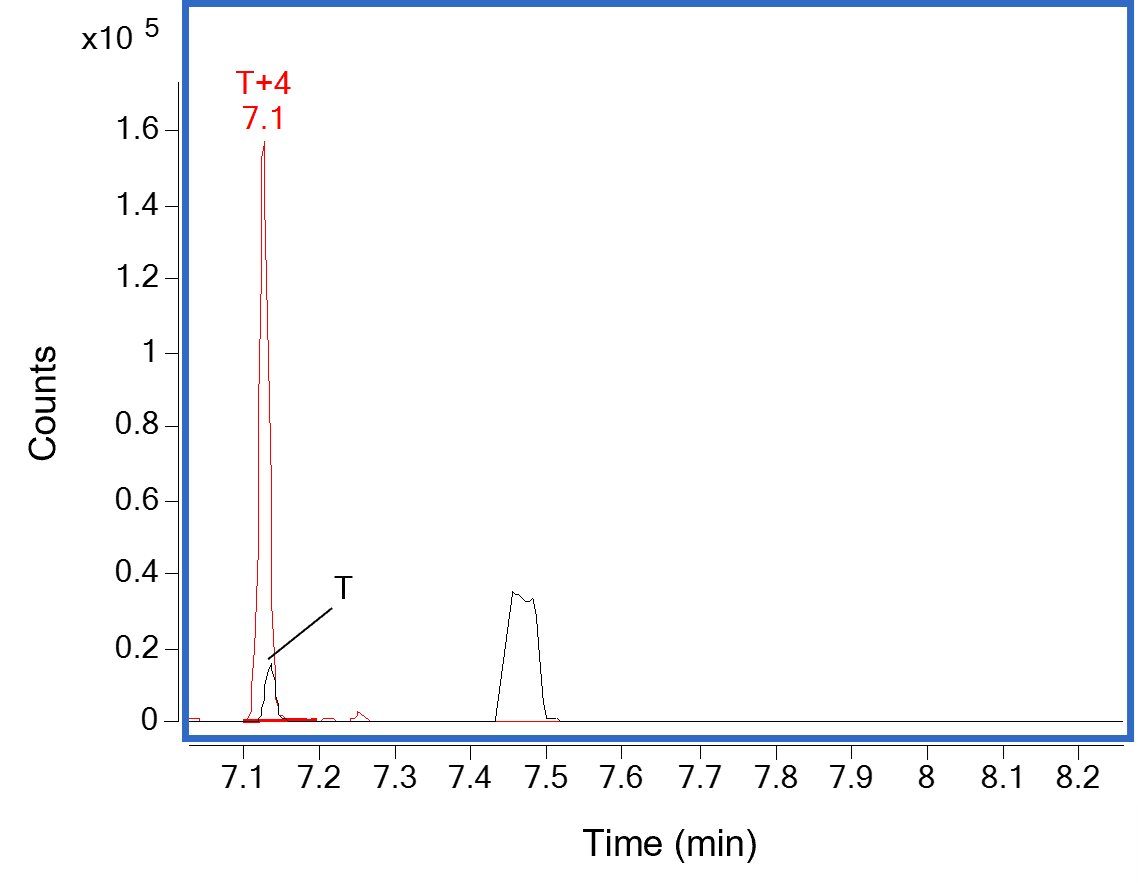


**Figure S14. hMBD4 excises T from a T:G-containing oligonucleotide as verified by GC-MS.** A duplex oligonucleotide containing a T:G mispair (110 pmol) was prepared in buffer containing 10 mM potassium phosphate pH 7.7, 30 mM sodium chloride and 40 mM potassium chloride and 110 pmol of a T+4 stable isotope internal standard. The oligonucleotides and free base standard were incubated with hMBD4 (112 pmoles) at 37 ^o^C for 2 h in a 110 mL reaction volume. Free bases were separated from enzymes and oligonucleotides by spin filtration, 90% of sample was dried under reduced pressure and derivatized to TBDMS derivatives. Derivatized free bases were separated by GC and identified by MS with selected ion monitoring. The black chromatogram above (*m/z* 297) corresponds to the T released by hMBD4 from the T:G-containing oligonucleotide duplex and the red chromatogram (*m/z* 301) corresponds to the isotope enriched internal standard (T+4). Identification of T release is confirmed by coelution of T and T+4 detected at the appropriates values of *m/z*.

**
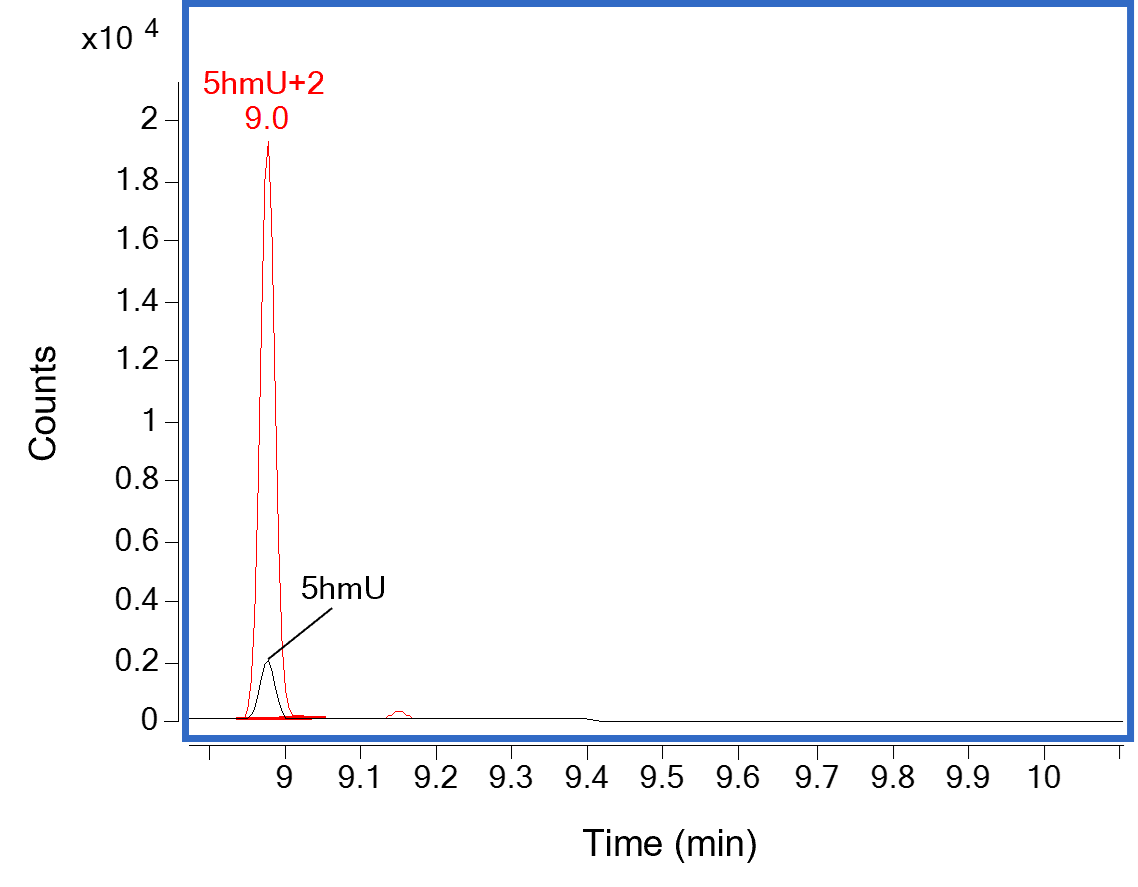
**

**Figure S15. hMBD4 excises 5hmU from a 5hmU:G-containing oligonucleotide as verified by GC-MS.** A duplex oligonucleotide containing a 5hmU:G mispair (110 pmol) was prepared in buffer containing 10 mM potassium phosphate pH 7.7, 30 mM sodium chloride and 40 mM potassium chloride and 110 pmol of a 5hmU+2 stable isotope internal standard. The oligonucleotides and free base standard were incubated with hMBD4 (112 pmoles) at 37 ^o^C for 2 h in a 110 mL reaction volume. Free bases were separated from enzymes and oligonucleotides by spin filtration, 90% of sample was dried under reduced pressure and derivatized to TBDMS derivatives. Derivatized free bases were separated by GC and identified by MS with selected ion monitoring. The black chromatogram above (*m/z* 427) corresponds to the 5hmU released by hMBD4 from the 5hmU:G-containing oligonucleotide duplex and the red chromatogram (*m/z* 429) corresponds to the isotope enriched internal standard (5hmU+2). Identification of 5hmU release is confirmed by coelution of 5hmU and 5hmU+2 detected at the appropriates values of *m/z*.


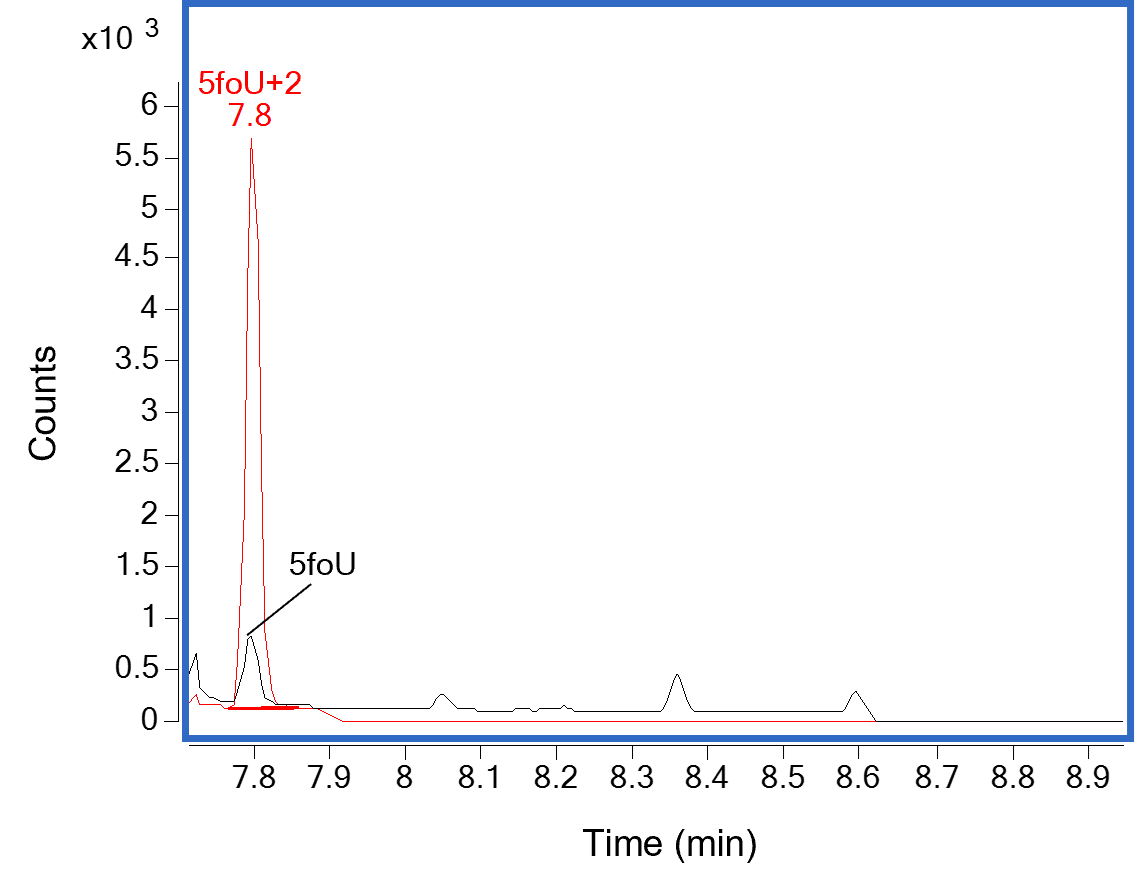


**Figure S16. hMBD4 excises 5foU from a 5foU:G-containing oligonucleotide as verified by GC-MS.** A duplex oligonucleotide containing a 5foU:G mispair (110 pmol) was prepared in buffer containing 10 mM potassium phosphate pH 7.7, 30 mM sodium chloride and 40 mM potassium chloride and 110 pmol of a 5foU+2 stable isotope internal standard. The oligonucleotides and free base standard were incubated with hMBD4 (112 pmoles) at 37 ^o^C for 2 h in a 110 mL reaction volume. Free bases were separated from enzymes and oligonucleotides by spin filtration, 90% of sample was dried under reduced pressure and derivatized to TBDMS derivatives. Derivatized free bases were separated by GC and identified by MS with selected ion monitoring. The black chromatogram above (*m/z* 311) corresponds to the 5foU released by hMBD4 from the 5foU:G-containing oligonucleotide duplex and the red chromatogram (*m/z* 313) corresponds to the isotope enriched internal standard (5foU+2). Identification of 5foU release is confirmed by coelution of 5foU and 5foU+2 detected at the appropriates values of *m/z*.


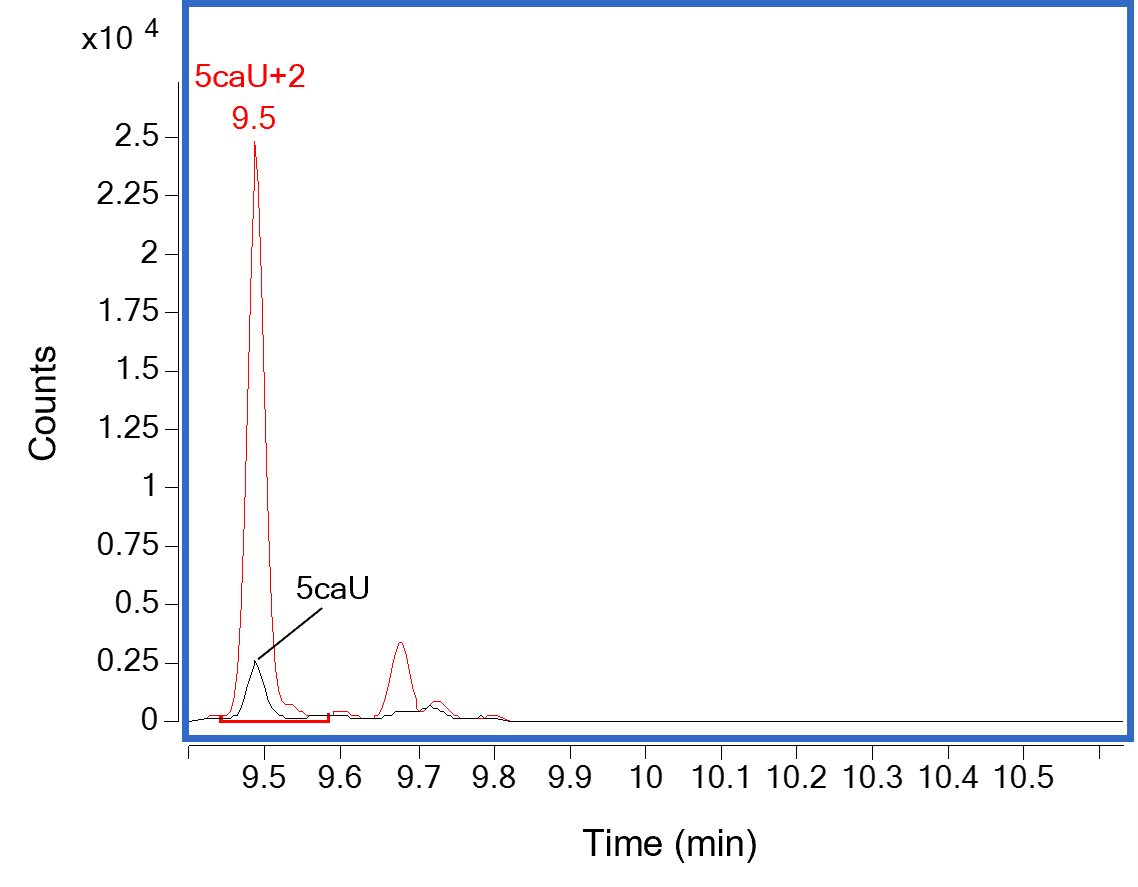


**Figure S17. hMBD4 excises 5caU from a 5caU:G-containing oligonucleotide as verified by GC-MS.** A duplex oligonucleotide containing a 5caU:G mispair (110 pmol) was prepared in buffer containing 10 mM potassium phosphate pH 7.7, 30 mM sodium chloride and 40 mM potassium chloride and 55 pmol of a 5caU+2 stable isotope internal standard. The oligonucleotides and free base standard were incubated with hMBD4 (112 pmoles) at 37 ^o^C for 2 h in a 110 mL reaction volume. Free bases were separated from enzymes and oligonucleotides by spin filtration, 90% of sample was dried under reduced pressure and derivatized to TBDMS derivatives. Derivatized free bases were separated by GC and identified by MS with selected ion monitoring. The black chromatogram above (*m/z* 441) corresponds to the 5caU released by hMBD4 from the 5caU:G-containing oligonucleotide duplex and the red chromatogram (*m/z* 443) corresponds to the isotope enriched internal standard (5caU+2). Identification of 5caU release is confirmed by coelution of 5caU and 5caU+2 detected at the appropriates values of *m/z*.


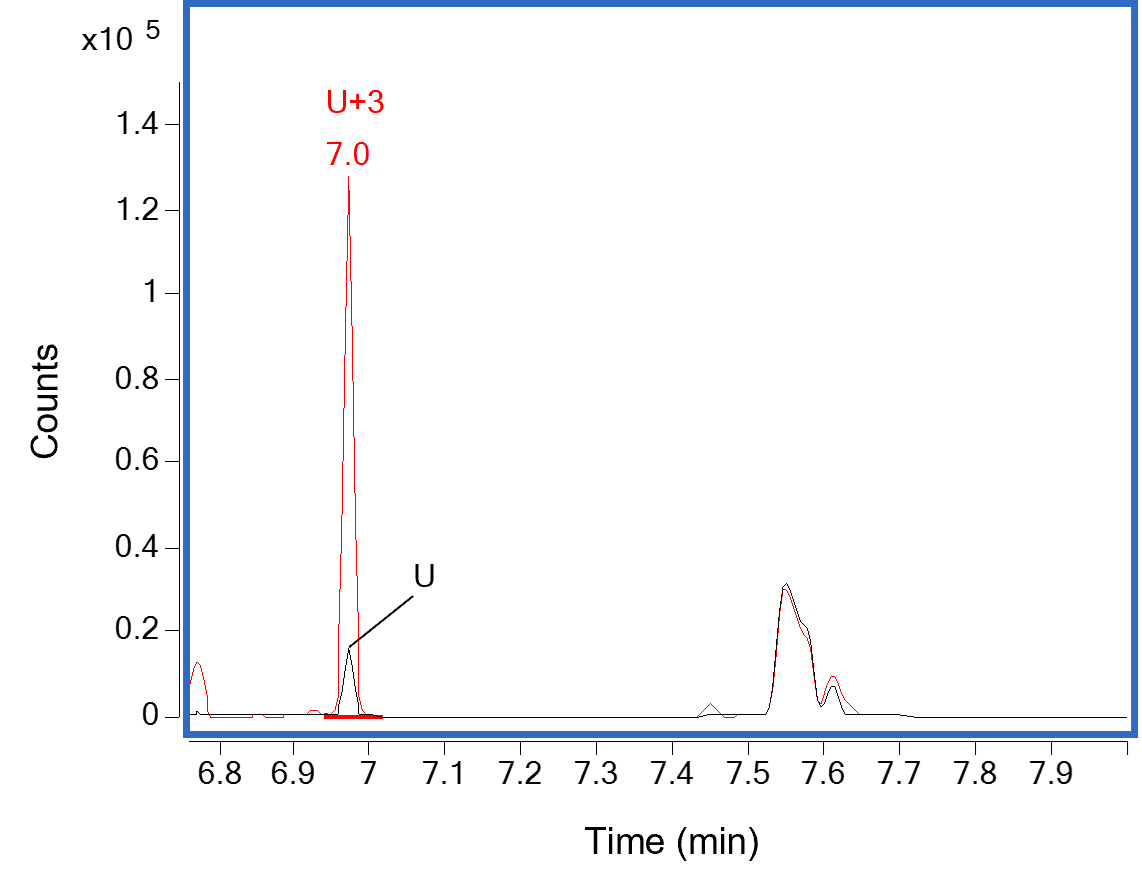


**Figure S18. hMBD4 excises U from a U:A-containing oligonucleotide as verified by GC-MS.** A duplex oligonucleotide containing a U:A mispair (110 pmol) was prepared in buffer containing 10 mM potassium phosphate pH 7.7, 30 mM sodium chloride and 40 mM potassium chloride and 110 pmol of a U+3 stable isotope internal standard. The oligonucleotides and free base standard were incubated with hMBD4 (112 pmoles) at 37 ^o^C for 2 h in a 110 mL reaction volume. Free bases were separated from enzymes and oligonucleotides by spin filtration, 90% of sample was dried under reduced pressure and derivatized to TBDMS derivatives. Derivatized free bases were separated by GC and identified by MS with selected ion monitoring. The black chromatogram above (*m/z* 283) corresponds to the U released by hMBD4 from the U:A-containing oligonucleotide duplex and the red chromatogram (*m/z* 286) corresponds to the isotope enriched internal standard (U+3). Identification of U release is confirmed by coelution of U and U+3 detected at the appropriates values of *m/z*.

**
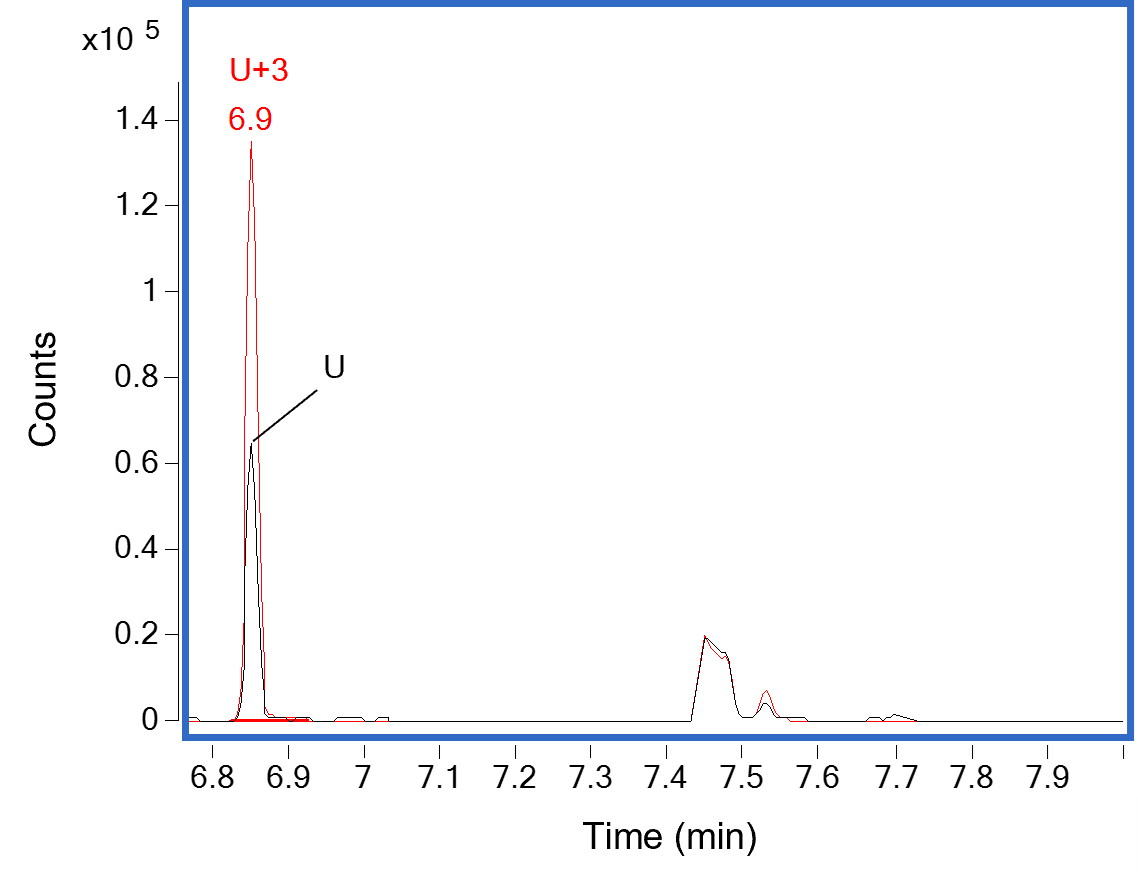
**

**Figure S19. hTDG excises U from a U:G-containing oligonucleotide as verified by GC-MS.** A duplex oligonucleotide containing a U:G mispair (110 pmol) was prepared in buffer containing 10 mM potassium phosphate pH 7.7, 30 mM sodium chloride and 40 mM potassium chloride and 110 pmol of a U+3 stable isotope internal standard. The oligonucleotides and free base standard were incubated with hTDG (110 pmoles) at 37 ^o^C for 2 h in a 110 mL reaction volume. Free bases were separated from enzymes and oligonucleotides by spin filtration, 90% of sample was dried under reduced pressure and derivatized to TBDMS derivatives. Derivatized free bases were separated by GC and identified by MS with selected ion monitoring. The black chromatogram above (*m/z* 283) corresponds to the U released by hTDG from the U:G-containing oligonucleotide duplex and the red chromatogram (*m/z* 286) corresponds to the isotope enriched internal standard (U+3). Identification of U release is confirmed by coelution of U and U+3 detected at the appropriates values of *m/z*.


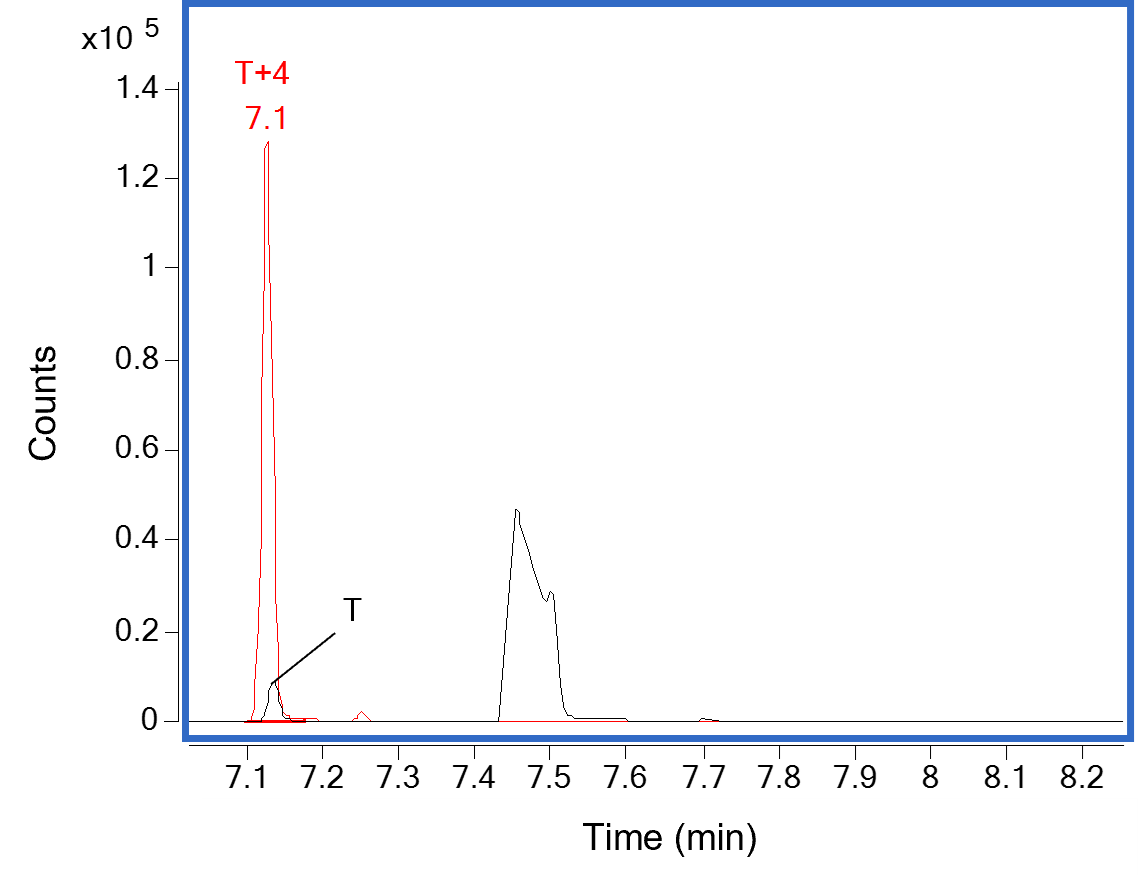


**Figure S20. hTDG excises T from a T:G-containing oligonucleotide as verified by GC-MS.** A duplex oligonucleotide containing a T:G mispair (110 pmol) was prepared in buffer containing 10 mM potassium phosphate pH 7.7, 30 mM sodium chloride and 40 mM potassium chloride and 110 pmol of a T+4 stable isotope internal standard. The oligonucleotides and free base standard were incubated with hTDG (110 pmoles) at 37 ^o^C for 2 h in a 110 mL reaction volume. Free bases were separated from enzymes and oligonucleotides by spin filtration, 90% of sample was dried under reduced pressure and derivatized to TBDMS derivatives. Derivatized free bases were separated by GC and identified by MS with selected ion monitoring. The black chromatogram above (*m/z* 297) corresponds to the T released by hTDG from the T:G-containing oligonucleotide duplex and the red chromatogram (*m/z* 301) corresponds to the isotope enriched internal standard (T+4). Identification of T release is confirmed by coelution of T and T+4 detected at the appropriates values of *m/z*.


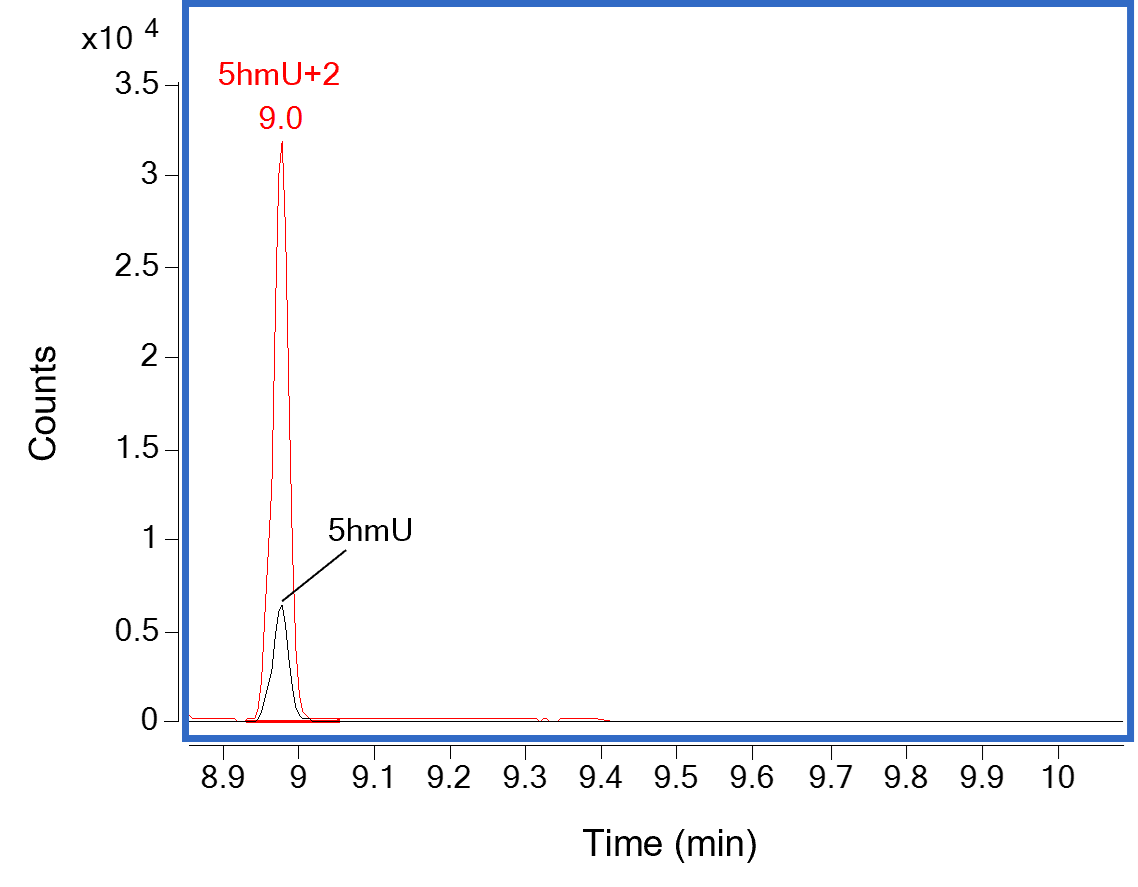


**Figure S21. hTDG excises 5hmU from a 5hmU:G-containing oligonucleotide as verified by GC-MS.** A duplex oligonucleotide containing a 5hmU:G mispair (110 pmol) was prepared in buffer containing 10 mM potassium phosphate pH 7.7, 30 mM sodium chloride and 40 mM potassium chloride and 110 pmol of a 5hmU+2 stable isotope internal standard. The oligonucleotides and free base standard were incubated with hTDG (110 pmoles) at 37 ^o^C for 2 h in a 110 mL reaction volume. Free bases were separated from enzymes and oligonucleotides by spin filtration, 90% of sample was dried under reduced pressure and derivatized to TBDMS derivatives. Derivatized free bases were separated by GC and identified by MS with selected ion monitoring. The black chromatogram above (*m/z* 427) corresponds to the 5hmU released by hTDG from the 5hmU:G-containing oligonucleotide duplex and the red chromatogram (*m/z* 429) corresponds to the isotope enriched internal standard (5hmU+2). Identification of 5hmU release is confirmed by coelution of 5hmU and 5hmU+2 detected at the appropriates values of *m/z*.


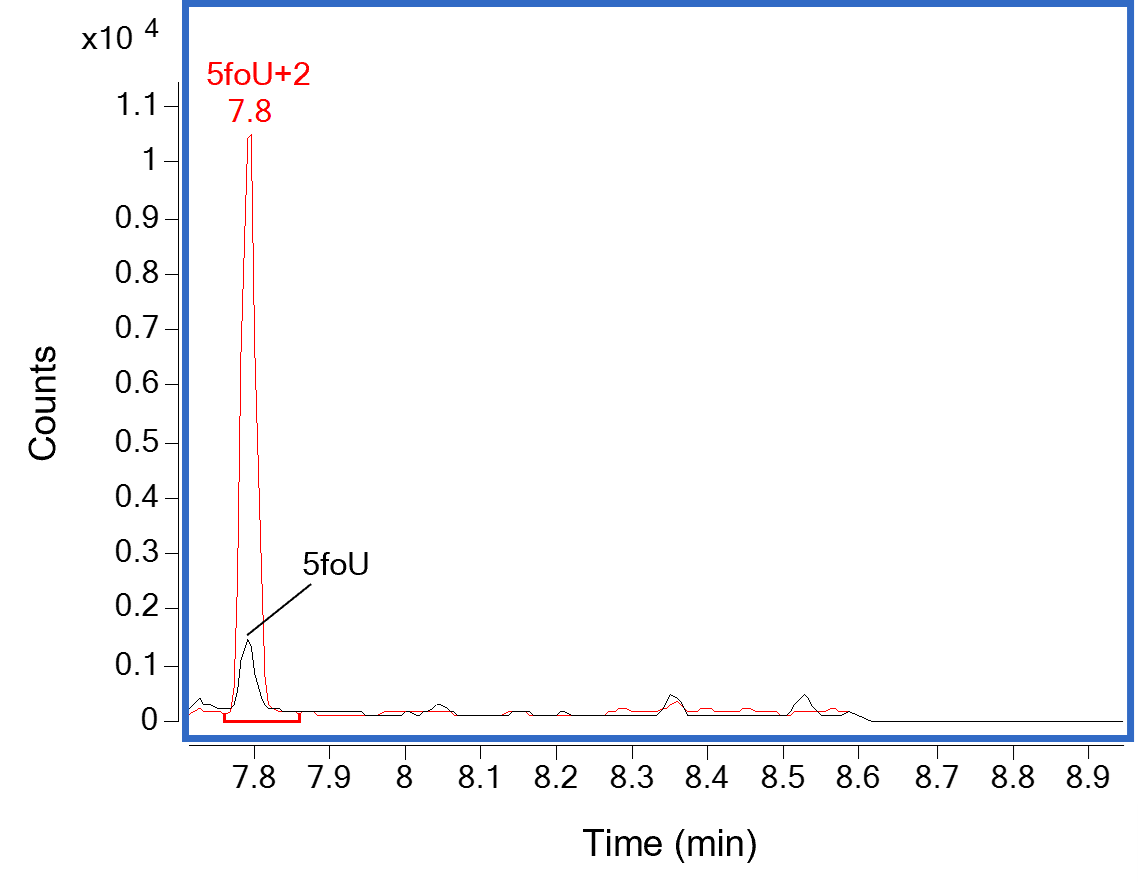


**Figure S22. hTDG excises 5foU from a 5foU:G-containing oligonucleotide as verified by GC-MS.** A duplex oligonucleotide containing a 5foU:G mispair (110 pmol) was prepared in buffer containing 10 mM potassium phosphate pH 7.7, 30 mM sodium chloride and 40 mM potassium chloride and 110 pmol of a 5foU+2 stable isotope internal standard. The oligonucleotides and free base standard were incubated with hTDG (110 pmoles) at 37 ^o^C for 2 h in a 110 mL reaction volume. Free bases were separated from enzymes and oligonucleotides by spin filtration, 90% of sample was dried under reduced pressure and derivatized to TBDMS derivatives. Derivatized free bases were separated by GC and identified by MS with selected ion monitoring. The black chromatogram above (*m/z* 311) corresponds to the 5foU released by hTDG from the 5foU:G-containing oligonucleotide duplex and the red chromatogram (*m/z* 313) corresponds to the isotope enriched internal standard (5foU+2). Identification of 5foU release is confirmed by coelution of 5foU and 5foU+2 detected at the appropriates values of *m/z*.


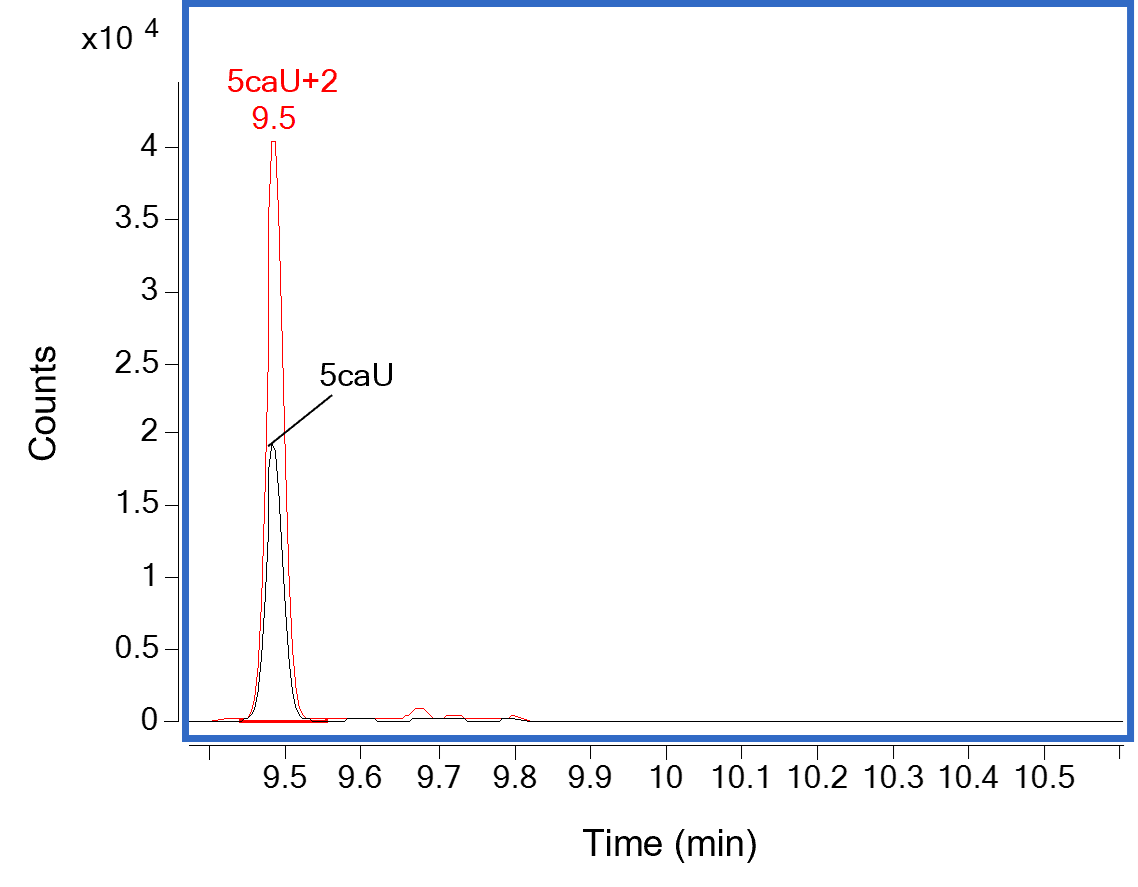


**Figure S23. hTDG excises 5caU from a 5caU:G-containing oligonucleotide as verified by GC-MS.** A duplex oligonucleotide containing a 5caU:G mispair (110 pmol) was prepared in buffer containing 10 mM potassium phosphate pH 7.7, 30 mM sodium chloride and 40 mM potassium chloride and 55 pmol of a 5caU+2 stable isotope internal standard. The oligonucleotides and free base standard were incubated with hTDG (110 pmoles) at 37 ^o^C for 2 h in a 110 mL reaction volume. Free bases were separated from enzymes and oligonucleotides by spin filtration, 90% of sample was dried under reduced pressure and derivatized to TBDMS derivatives. Derivatized free bases were separated by GC and identified by MS with selected ion monitoring. The black chromatogram above (*m/z* 441) corresponds to the 5caU released by hTDG from the 5caU:G-containing oligonucleotide duplex and the red chromatogram (*m/z* 443) corresponds to the isotope enriched internal standard (5caU+2). Identification of 5caU release is confirmed by coelution of 5caU and 5caU+2 detected at the appropriates values of *m/z*.


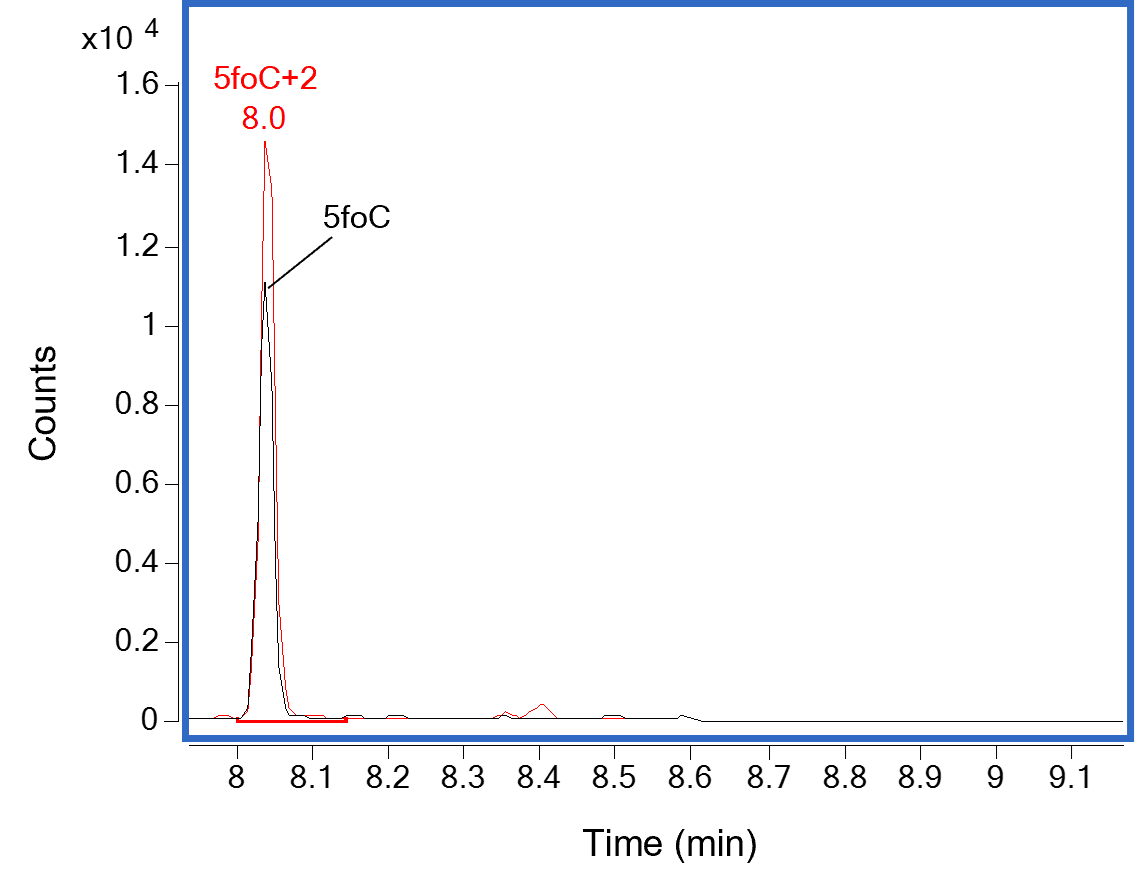


**Figure S24. hTDG excises 5foC from a 5foC:G-containing oligonucleotide as verified by GC-MS.** A duplex oligonucleotide containing a 5foC:G mispair (110 pmol) was prepared in buffer containing 10 mM potassium phosphate pH 7.7, 30 mM sodium chloride and 40 mM potassium chloride and 110 pmol of a 5foC+2 stable isotope internal standard. The oligonucleotides and free base standard were incubated with hTDG (110 pmoles) at 37 ^o^C for 2 h in a 110 mL reaction volume. Free bases were separated from enzymes and oligonucleotides by spin filtration, 90% of sample was dried under reduced pressure and derivatized to TBDMS derivatives. Derivatized free bases were separated by GC and identified by MS with selected ion monitoring. The black chromatogram above (*m/z* 310) corresponds to the 5foC released by hTDG from the 5foC:G-containing oligonucleotide duplex and the red chromatogram (*m/z* 312) corresponds to the isotope enriched internal standard (5foC+2). Identification of 5foC release is confirmed by coelution of 5foC and 5foC+2 detected at the appropriates values of *m/z*.


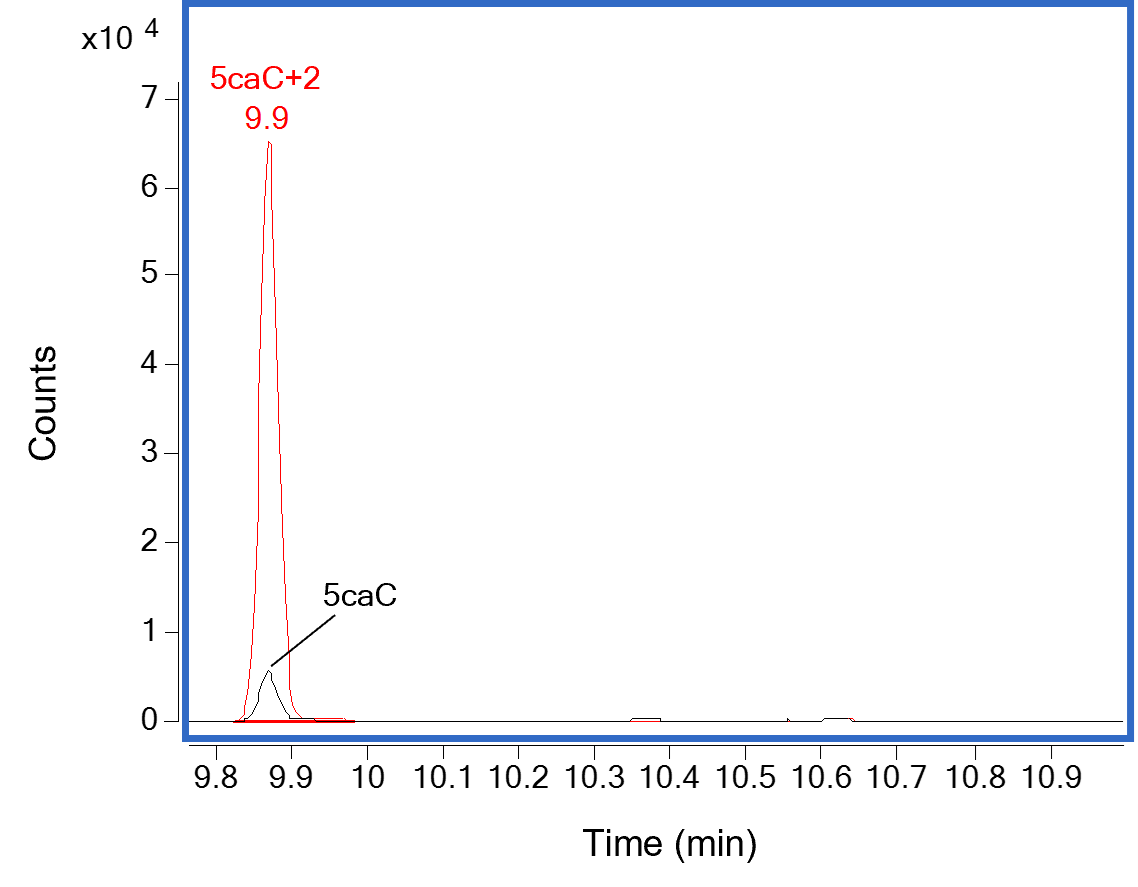


**Figure S25. hTDG excises 5caC from a 5caC:G-containing oligonucleotide as verified by GC-MS.** A duplex oligonucleotide containing a 5caC:G mispair (110 pmol) was prepared in buffer containing 10 mM potassium phosphate pH 7.7, 30 mM sodium chloride and 40 mM potassium chloride and 55 pmol of a 5caC+2 stable isotope internal standard. The oligonucleotides and free base standard were incubated with hTDG (110 pmoles) at 37 ^o^C for 2 h in a 110 mL reaction volume. Free bases were separated from enzymes and oligonucleotides by spin filtration, 90% of sample was dried under reduced pressure and derivatized to TBDMS derivatives. Derivatized free bases were separated by GC and identified by MS with selected ion monitoring. The black chromatogram above (*m/z* 440) corresponds to the 5caC released by hTDG from the 5caC:G-containing oligonucleotide duplex and the red chromatogram (*m/z* 442) corresponds to the isotope enriched internal standard (5caC+2). Identification of 5caC release is confirmed by coelution of 5caC and 5caC+2 detected at the appropriates values of *m/z*.

**
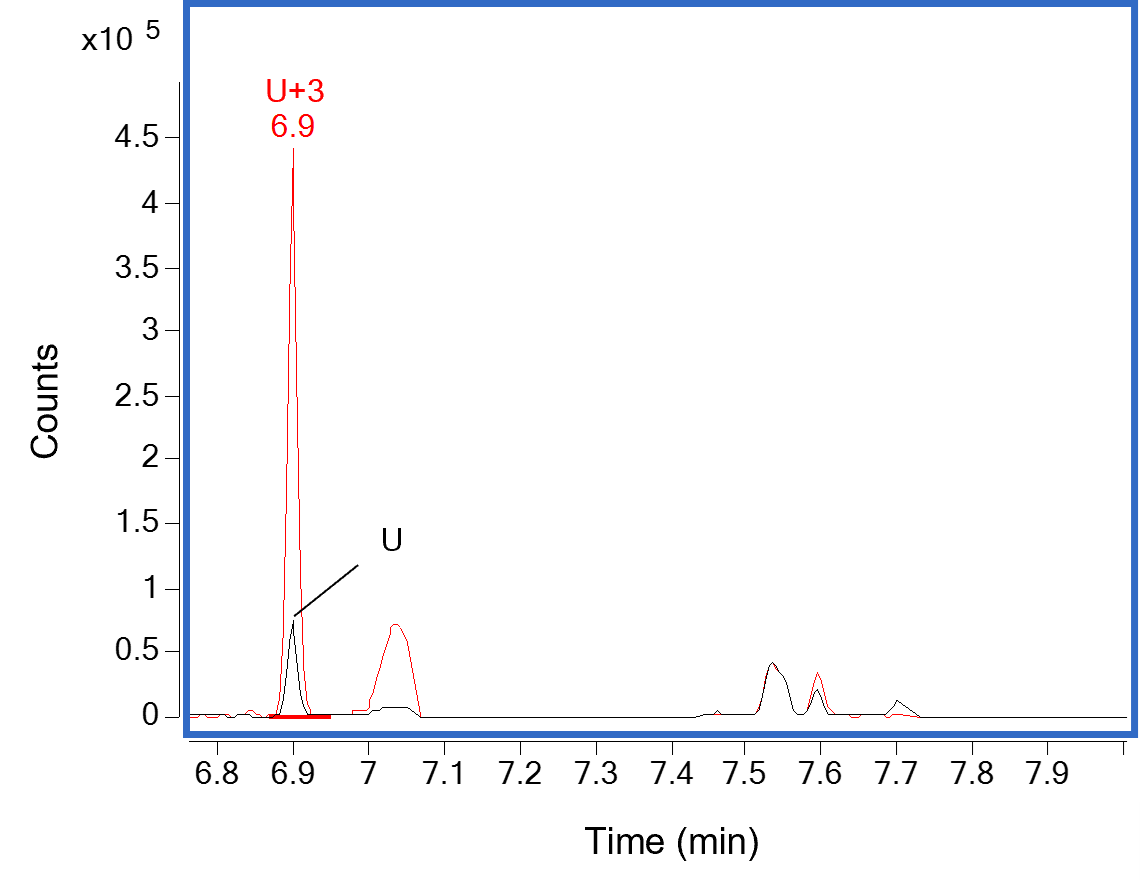
**

**Figure S26. hTDG excises U from a U:A-containing oligonucleotide as verified by GC-MS.** A duplex oligonucleotide containing a U:A mispair (110 pmol) was prepared in buffer containing 10 mM potassium phosphate pH 7.7, 30 mM sodium chloride and 40 mM potassium chloride and 110 pmol of a U+3 stable isotope internal standard. The oligonucleotides and free base standard were incubated with hTDG (110 pmoles) at 37 ^o^C for 2 h in a 110 mL reaction volume. Free bases were separated from enzymes and oligonucleotides by spin filtration, 90% of sample was dried under reduced pressure and derivatized to TBDMS derivatives. Derivatized free bases were separated by GC and identified by MS with selected ion monitoring. The black chromatogram above (*m/z* 283) corresponds to the U released by hTDG from the U:A-containing oligonucleotide duplex and the red chromatogram (*m/z* 286) corresponds to the isotope enriched internal standard (U+3). Identification of U release is confirmed by coelution of U and U+3 detected at the appropriates values of *m/z*.


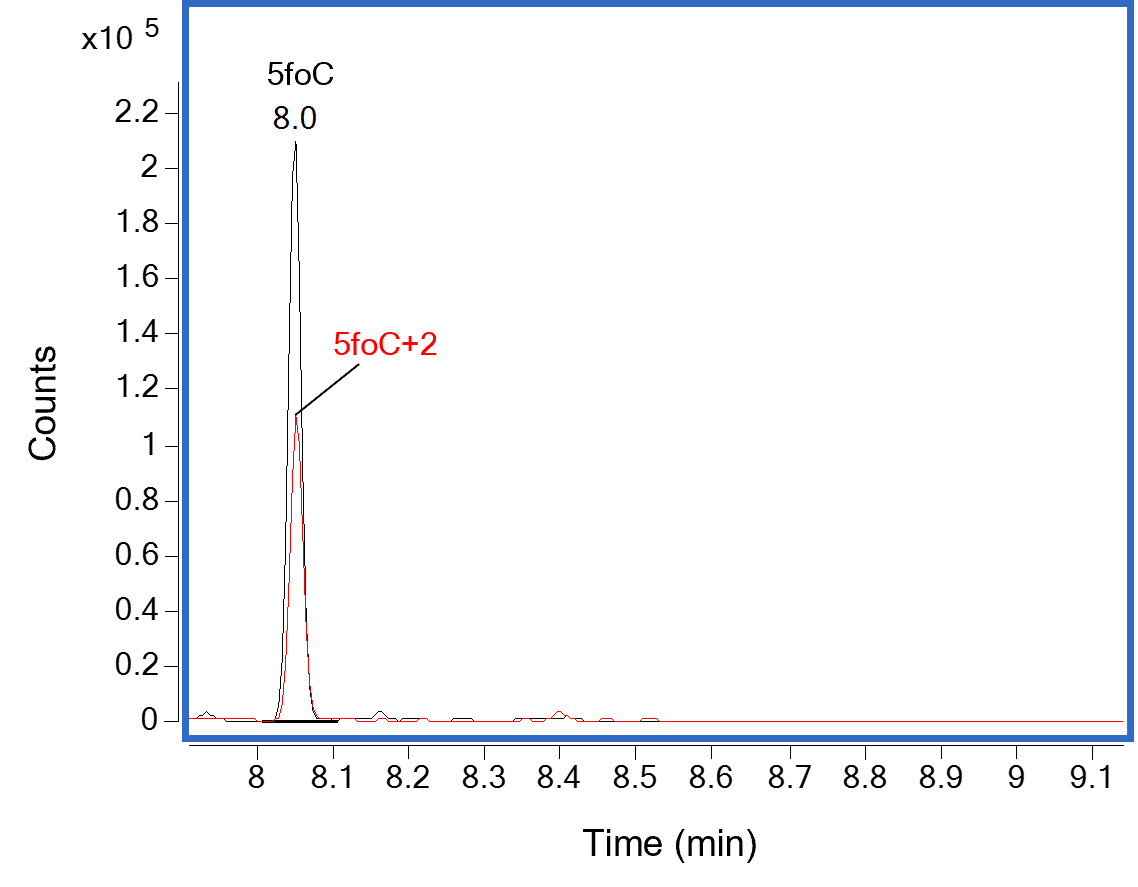


**Figure S27. hTDG excises 5foC from a 5foC:A-containing oligonucleotide as verified by GC-MS.** A duplex oligonucleotide containing a 5foC:A mispair (110 pmol) was prepared in buffer containing 10 mM potassium phosphate pH 7.7, 30 mM sodium chloride and 40 mM potassium chloride and 110 pmol of a 5foC+2 stable isotope internal standard. The oligonucleotides and free base standard were incubated with hTDG (110 pmoles) at 37 ^o^C for 2 h in a 110 mL reaction volume. Free bases were separated from enzymes and oligonucleotides by spin filtration, 90 % of sample was dried under reduced pressure and derivatized to TBDMS derivatives. Derivatized free bases were separated by GC and identified by MS with selected ion monitoring. The black chromatogram above (*m/z* 310) corresponds to the 5foC released by hTDG from the 5foC:A-containing oligonucleotide duplex and the red chromatogram (*m/z* 312) corresponds to the isotope enriched internal standard (5foC+2). Identification of 5foC release is confirmed by coelution of 5foC and 5foC+2 detected at the appropriates values of *m/z*.


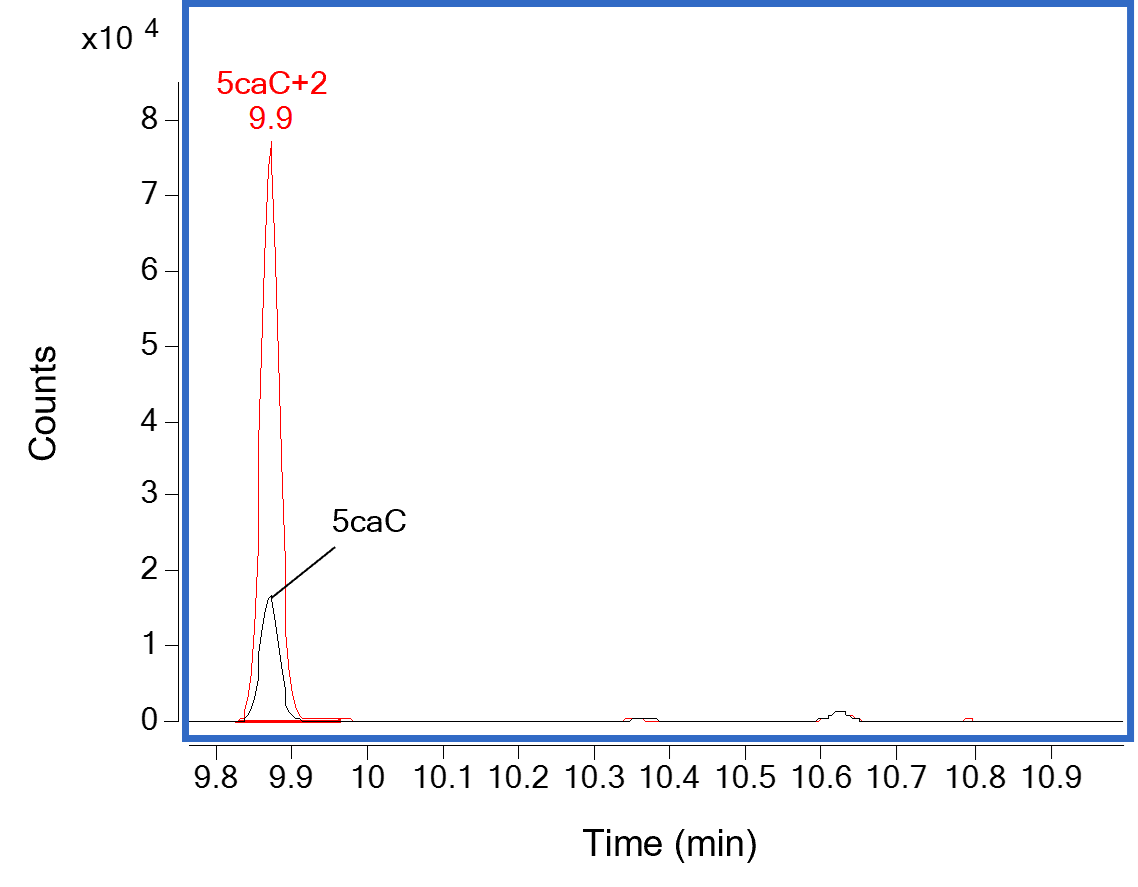


**Figure S28. hTDG excises 5caC from a 5caC:A-containing oligonucleotide as verified by GC-MS.** A duplex oligonucleotide containing a 5caC:A mispair (110 pmol) was prepared in buffer containing 10 mM potassium phosphate pH 7.7, 30 mM sodium chloride and 40 mM potassium chloride and 55 pmol of a 5caC+2 stable isotope internal standard. The oligonucleotides and free base standard were incubated with hTDG (110 pmoles) at 37 ^o^C for 2 h in a 110 mL reaction volume. Free bases were separated from enzymes and oligonucleotides by spin filtration, 90 % of sample was dried under reduced pressure and derivatized to TBDMS derivatives. Derivatized free bases were separated by GC and identified by MS with selected ion monitoring. The black chromatogram above (*m/z* 440) corresponds to the 5caC released by hTDG from the 5caC:A-containing oligonucleotide duplex and the red chromatogram (*m/z* 442) corresponds to the isotope enriched internal standard (5caC+2). Identification of 5caC release is confirmed by coelution of 5caC and 5caC+2 detected at the appropriates values of *m/z*.
