# Supplementary material for: Chemical and enzymatic modifications of 5-methylcytosine at the intersection of DNA damage, repair, and epigenetic reprogramming
Source: PLoS One. 2022 Aug 29;17(8):e0273509. doi: 10.1371/journal.pone.0273509 (PMC9423628; doi:10.1371/journal.pone.0273509)
Supplement: S1 Raw images — (PDF) [file pone.0273509.s002.pdf]

hUNG2

U:G	U:A

U:G

gel1:

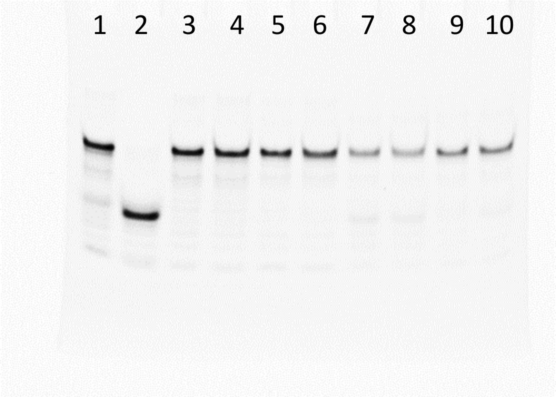

gel2:

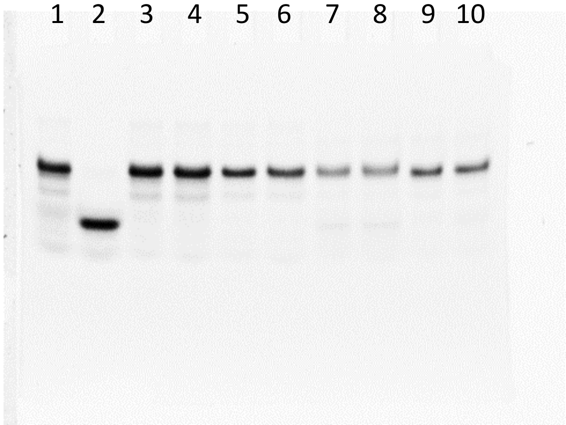

gel3:

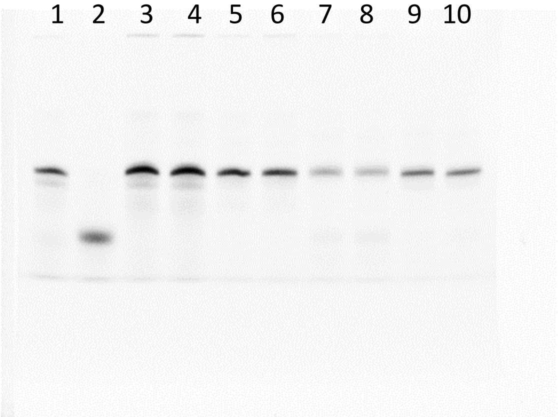

U:A

gel1:

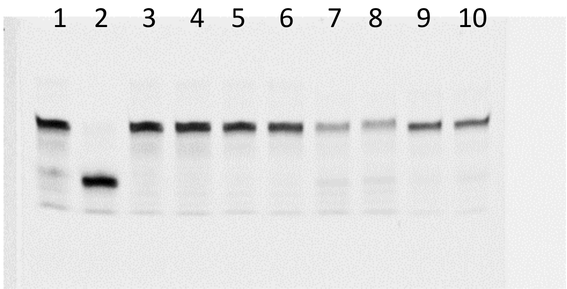

gel2:

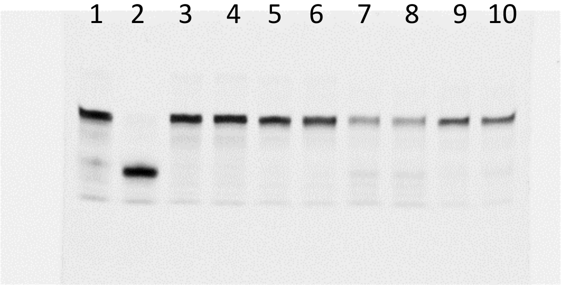

gel3:

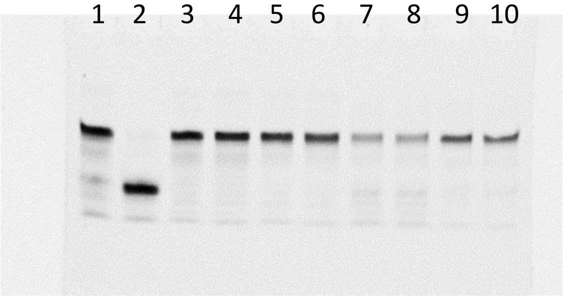

hUNG2

C:G

gel1:

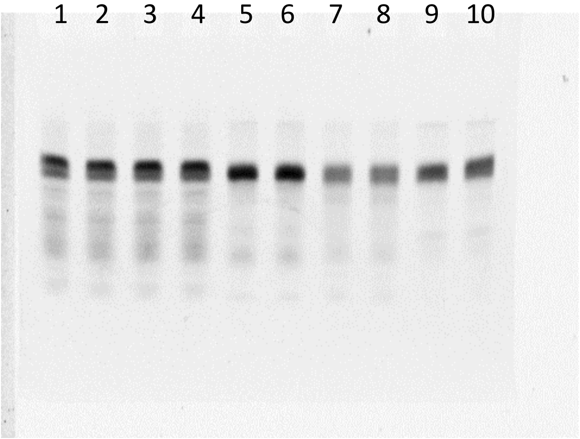

gel2:

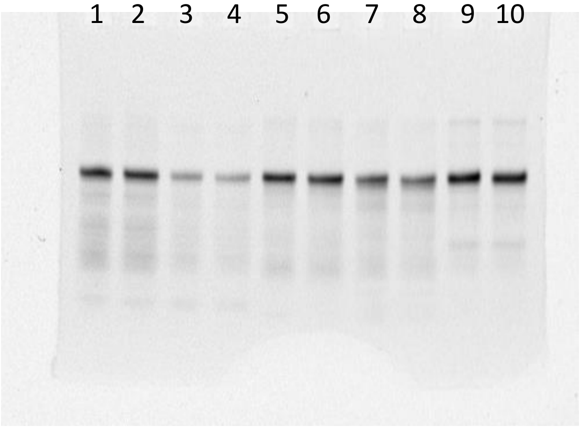

gel3:

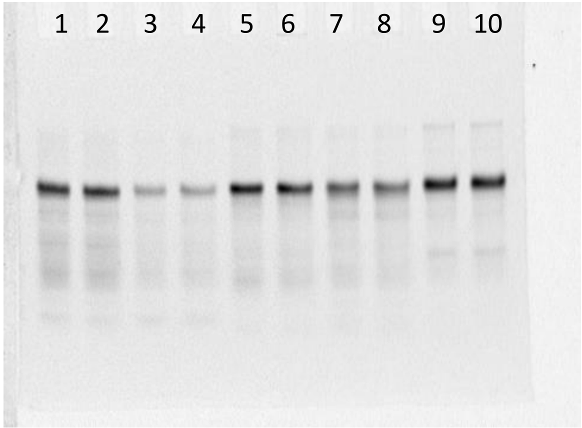

C:A

gel1:

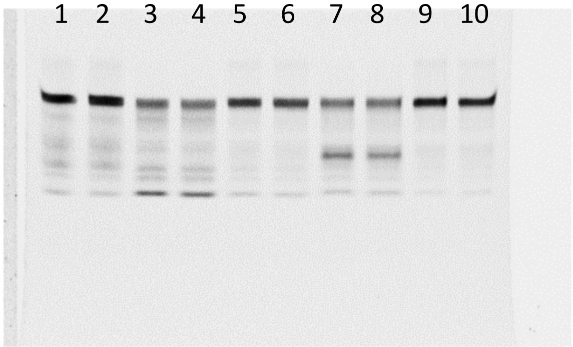

gel2:

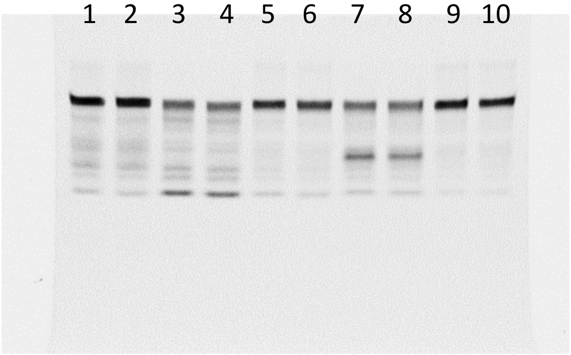

gel3:

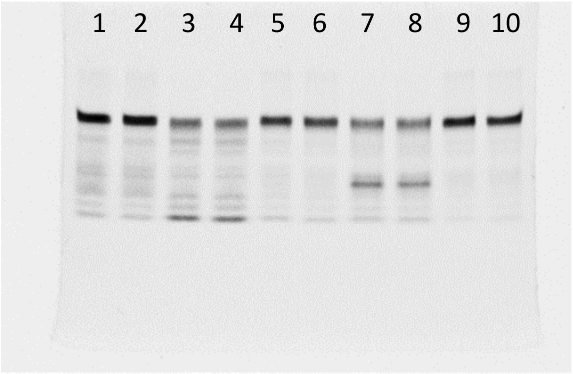

hSMUG1

UG

gel1:

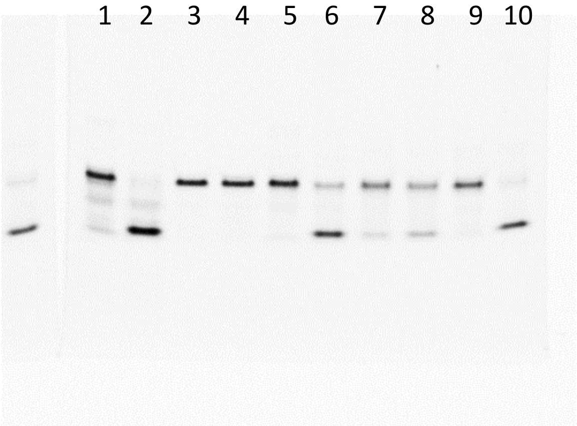

gel2:

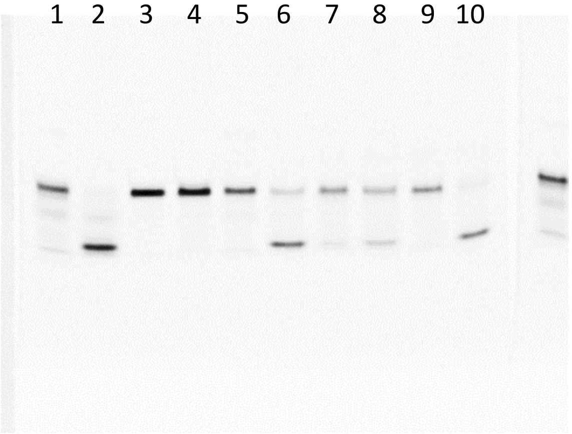

gel3:

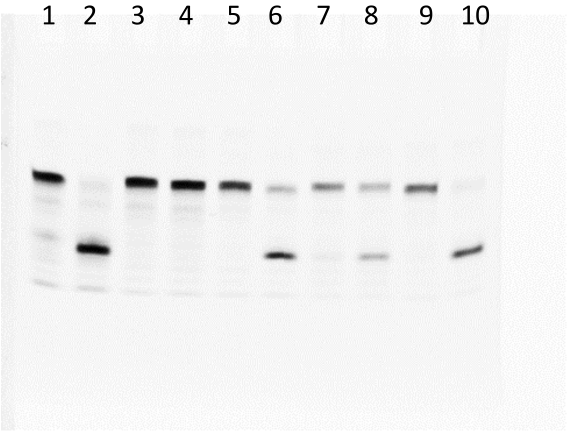

U:A

gel1:

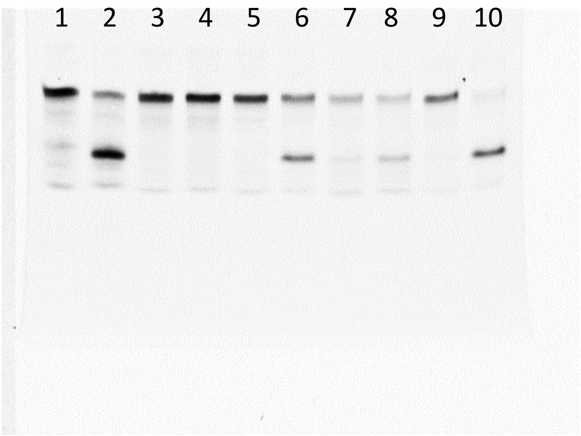

gel2:

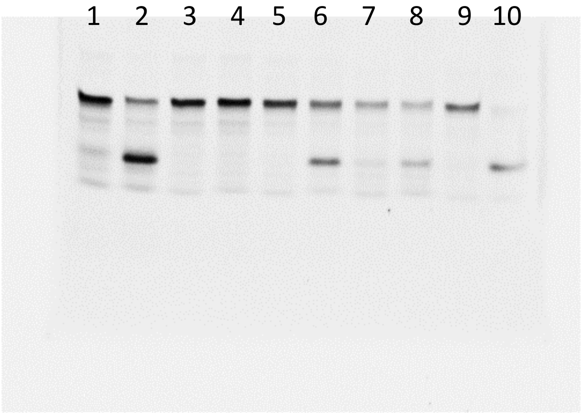

gel3:

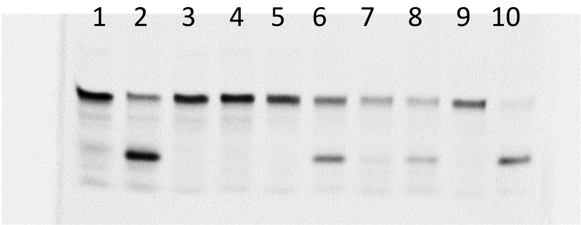

hSMUG1

C:G

gel1:

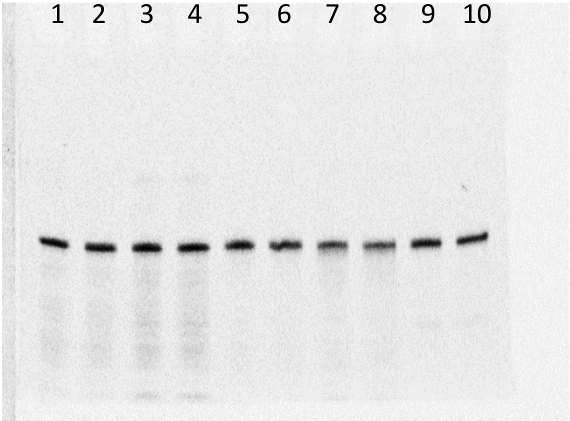

gel2:

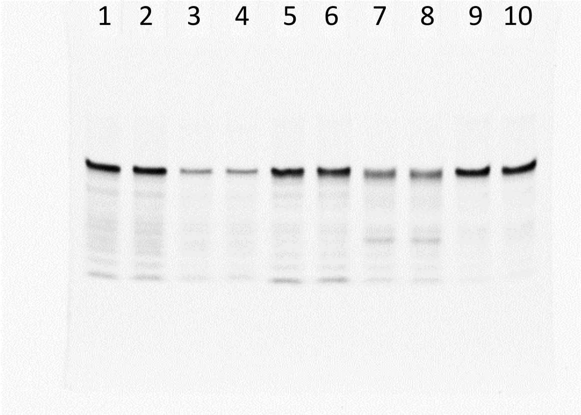

gel3:

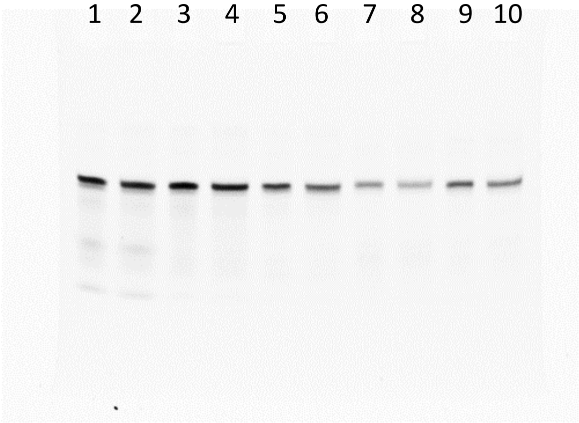

C:A

gel1:

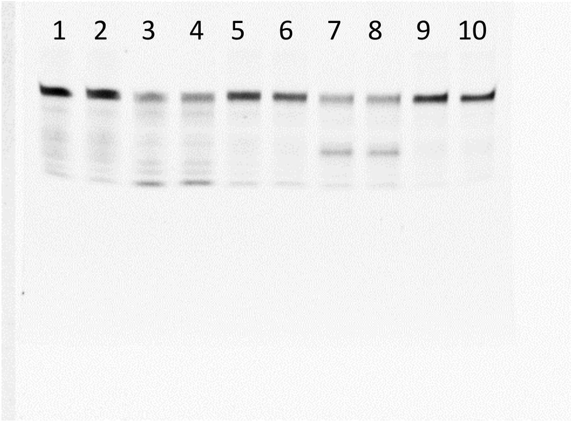

gel2:

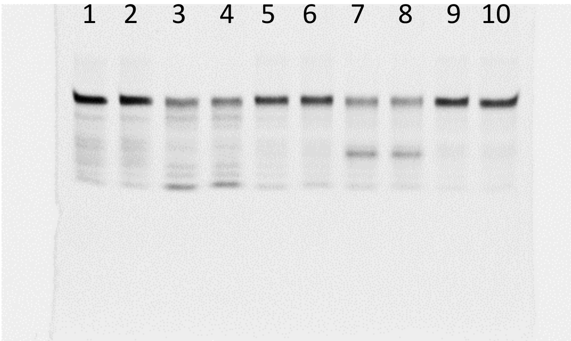

gel3:

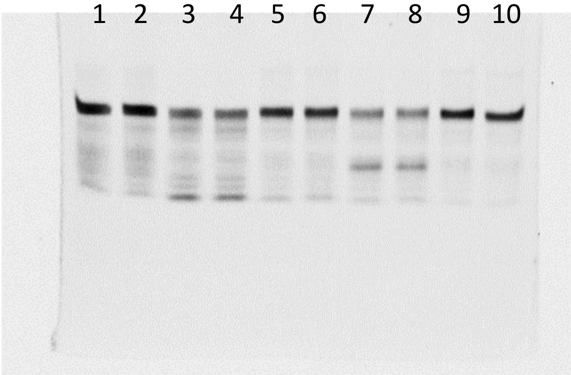

hTDG

UG

gel1:

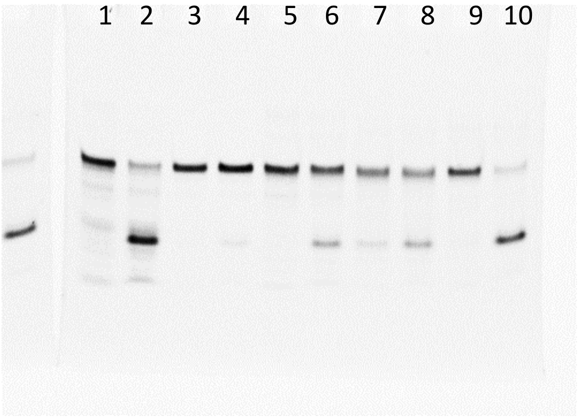

gel2:

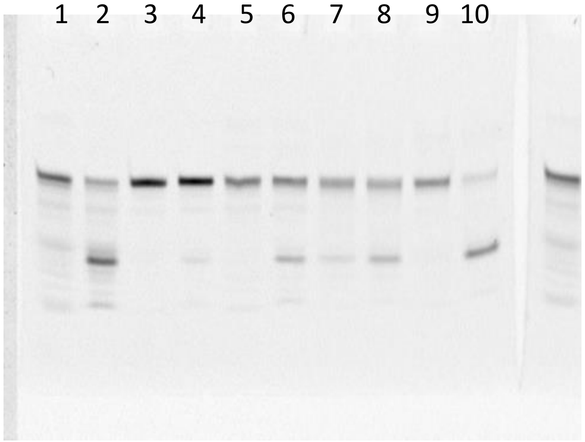

gel3:

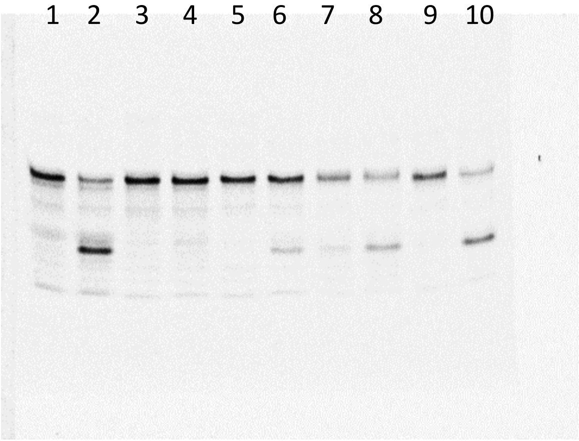

U:A

gel1:

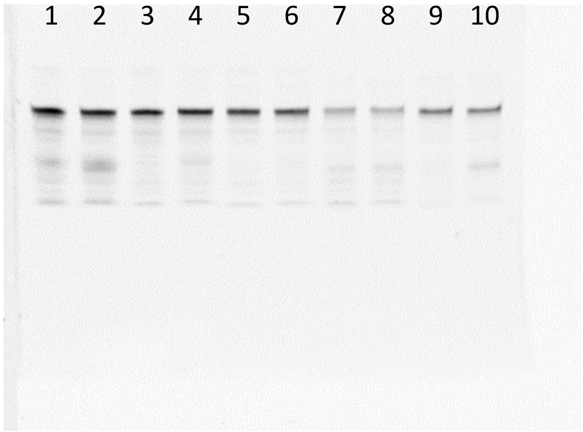

gel2:

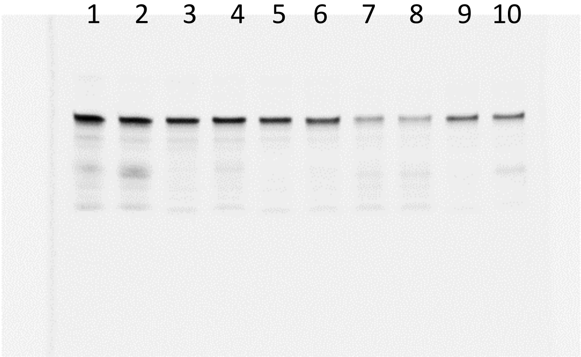

gel3:

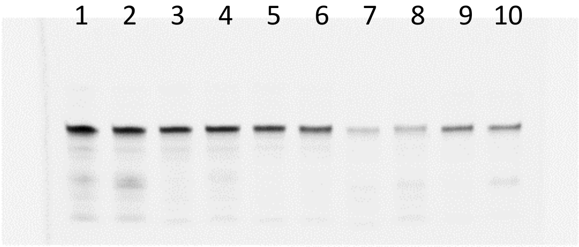

hTDG

C:G

gel1:

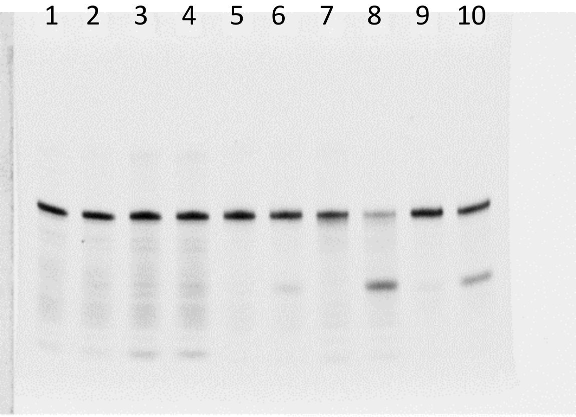

gel2:

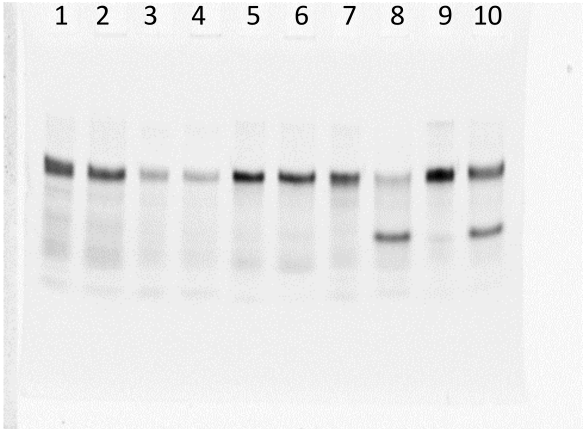

gel3:

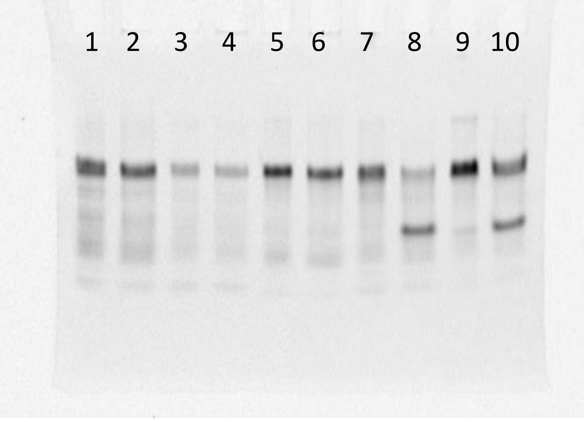

C:A

gel1:

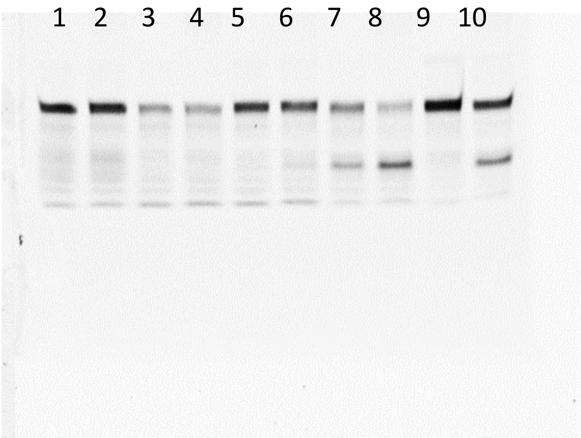

gel2:

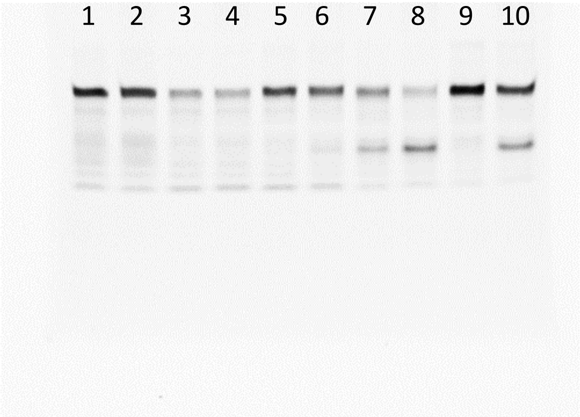

gel3:

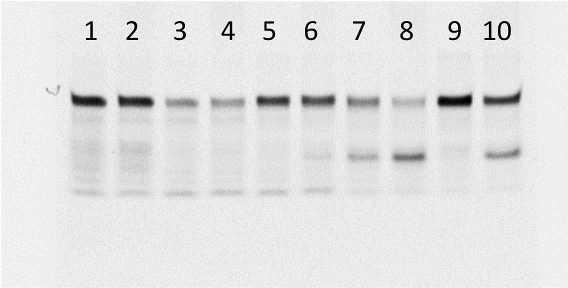

hMBD4

UG

gel1:

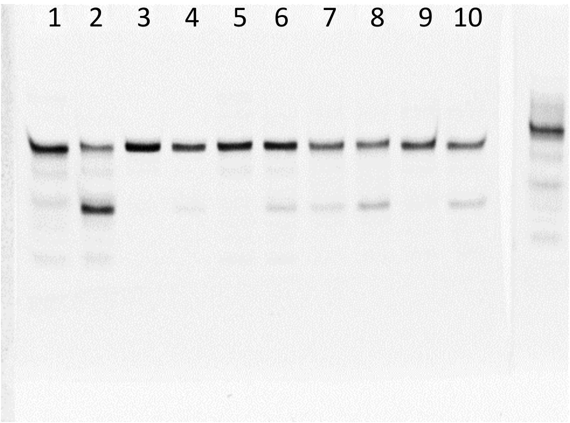

gel2:

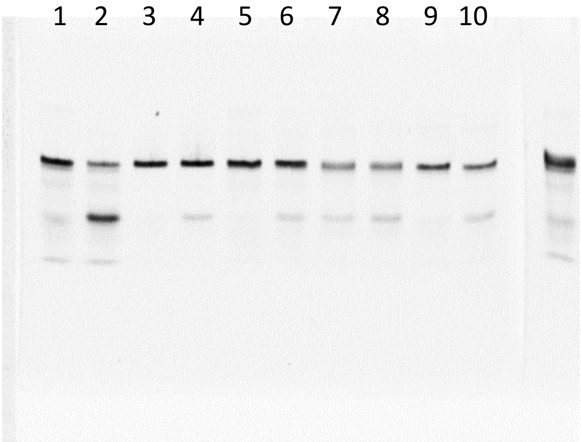

gel3:

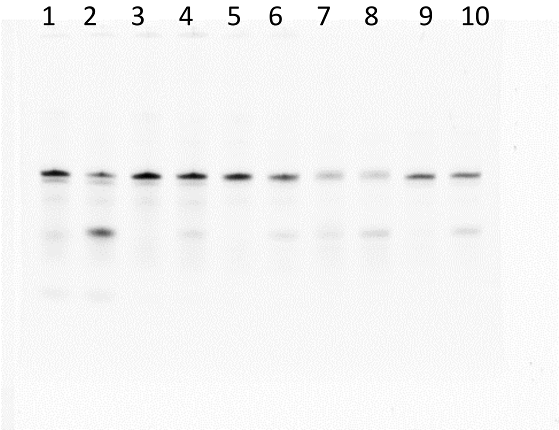

U:A

gel1:

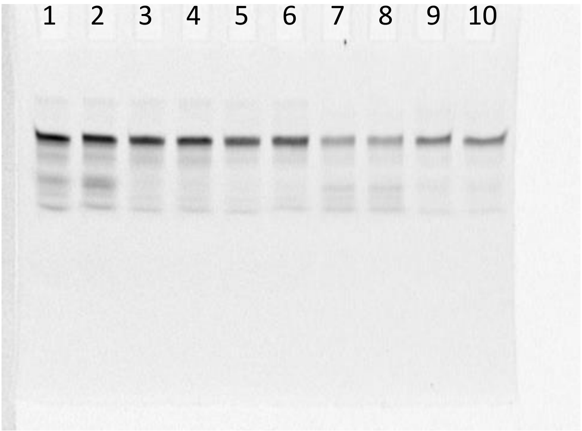

gel2:

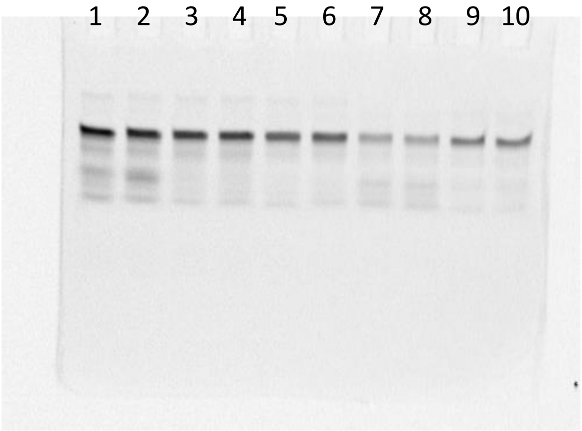

gel3:

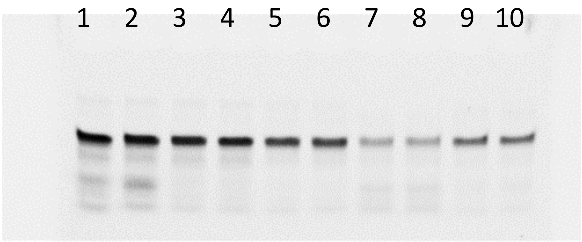

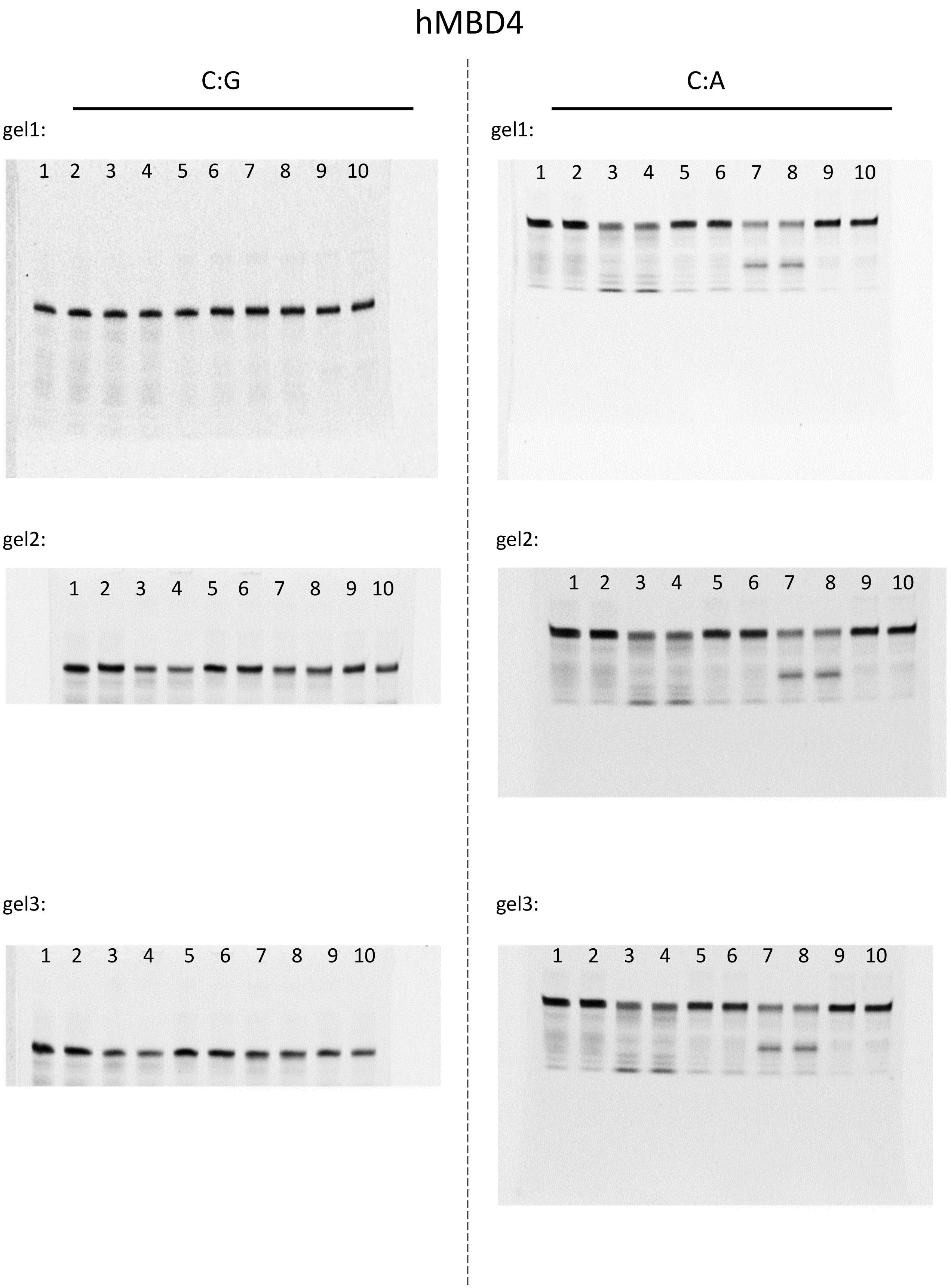
hMBD4
C:G
C:A
gel1:
1 2 3 4 5 6 7 8 9 10
gel1:
1 2 3 4 5 6 7 8 9 10
gel2:
1 2 3 4 5 6 7 8 9 10
gel2:
1 2 3 4 5 6 7 8 9 10
gel3:
1 2 3 4 5 6 7 8 9 10
gel3:
1 2 3 4 5 6 7 8 9 10

Figure legend:

Uncropped and unadjusted original gel pictures. Oligonucleotides containing uracil (U) analogs, or cytosine (C) analogs paired with guanine (G) and adenine (A) treated with human UNG2 (hUNG2) (**page 1, 2**), human SMUG1 (hSMUG1) (**page 3, 4**), human TDG (hTDG (**page 5, 6**), and human MBD4 (hMBD4) (**page 7, 8**). Sample order for uracil analogs (shown on pages 1, 3, 5, 7): lanes: 1, 2 (U:G or A), 3, 4: (T:G or A),: 5, 6 (hmU:G or A), 7, 8 (foU:G or A) and 9, 10 : caU:G or A. Sample order of cytosine analogs (shown on page 2, 4, 6, 8): lanes 1, 2 (C:G or A), 3, 4: (mC:G or A),: 5, 6 (hmC:G or A), 7, 8 (foC:G or A) and 9, 10 : caC:G or A.
